# Seasonal variations of *Triatoma dimidiata* demography and *Trypanosoma cruzi* transmission within its multi-host community in the Yucatan Peninsula, Mexico: Insights from an integrative SIR eco-epidemiological modelling

**DOI:** 10.1371/journal.pntd.0014500

**Published:** 2026-07-15

**Authors:** Jean-Loup Zitoun, Eric Dumonteil, Julio Vladimir Cruz-Chan, Etienne Waleckx, Sébastien Gourbière

**Affiliations:** 1 Laboratorio de Parasitología, Centro de Investigaciones Regionales “Dr. Hideyo Noguchi”, Universidad Autonoma de Yucatán, Mérida, Yucatán, México; 2 Department of Tropical Medicine and Infectious Disease, Celia Scott Weatherhead, School of Public Health and Tropical Medicine, and Vector-Borne and Infectious Disease Research Center, Tulane University, New Orleans, Louisiana, United States of America; 3 Institut de Recherche pour le Développement, UMR INTERTRYP IRD, CIRAD, Université de Montpellier, Montpellier, France; 4 UMR5096 ‘Laboratoire Génome et Développement des Plantes’, Université de Perpignan Via Domitia, Perpignan, France; 5 School of Life Sciences, University of Sussex, Falmer, Brighton, United Kingdom; Kenya Agricultural and Livestock Research Organization, KENYA

## Abstract

Chagas disease is a zoonotic disease affecting 7 million people worldwide, mostly in Latin America. The causative agent, *Trypanosoma cruzi*, is transmitted by triatomine vectors feeding on a high diversity of vertebrate species. To understand its complex transmission dynamics is critical to assess disease risk and potential control strategies. We expanded a previous SI model of *T. cruzi* transmission in a typical synanthropic and domesticated host community into a SIR model to account for the effect of seasonal variations in the stage-structured vector demography and for the within-host parasite dynamics. The model was parameterized by using data on *Triatoma dimidiata* and hosts populations accumulated over the past 20 years in villages of the Yucatan peninsula, Mexico. Our modelling successfully reproduced 86.5% of the seasonal variations in vector abundance typically observed in those villages, with a peak of abundance during the warm and dry season (March to May), and it provided the first predictions of seasonal variations in the local *T. cruzi* transmission. Along with their abundance, vector infection was anticipated to rise by 23% from March to June, which closely matches a 19% increase in the field and led to a higher risk of *T. cruzi* transmission to all competent hosts. During this 3-months period, we predicted a two-fold increase in the incidence of *T. cruzi*, so that 39% of the yearly new infections occurred. Finally, sensitivity analyses provided evidence that reducing by 50% the current abundance of dogs, rodents, and increasing the current abundance of avians by 50% could reduce *T. cruzi* yearly incidence by up to ~38%, ~ 95%, and ~83%, respectively, supporting a promising potential for zooprophylactic control strategies that remain to be validated by empirical field studies. These findings strongly support the implementation of educational and empowerment strategies embedded in One Health approach to sustainably interrupt Chagas disease intra-domiciliary transmission.

## Author summary

Chagas disease is caused by the parasite *Trypanosoma cruzi* that predominantly spreads through the faeces of infected triatomine hematophagous vectors. There currently are 7 millions people infected worldwide, mostly in Latin America, and the main strategy to prevent Chagas disease’ vectorial transmission remains to limit contacts with vectors through insecticide spraying, house and backyard management and/or the use of screens. Epidemiological models are essential tools to understand the complex dynamics of *T. cruzi* transmission through the large network of species which the bugs feed on, and to assess the potential of putative control strategies. By combining such models and data collected over the last 20 years in the Yucatan peninsula in Mexico, we show that there are seasonal variations in vector abundance and infection by the parasite that result in a 3-months period with twice as much parasite transmission as during the rest of the year. We further show that the size of avian, rodent and dog populations in villages can substantially modify the level of *T. cruzi* transmission to humans, which confirms that managing the host community offers a complementary way of controlling Chagas disease.

## Introduction

Vector-borne infectious diseases remain a critical public health challenge threatening over 80% of the world population [1] and accounting for 17% of the global estimated burden of all infectious diseases [2]. About half of the twenty Neglected Tropical Diseases (NTDs) are caused by vector-transmitted infectious agents [3] and, together with the directly- or environmentally-transmitted NTDs, are responsible for high levels of morbidity [4], affecting particularly the poorest populations living in the tropics and sub-tropics [5]. The lack of investments into NTDs research clearly impedes the development of an integrated knowledge of their biology, ecology and evolutionary potential, thereby limiting our ability to anticipate the efficacy of potential control strategies. Such a conundrum is especially difficult to overcome for vector-borne zoonotic NTDs whose spread, establishment and potential (re-)emergence entail complex interactions between pathogens, vectors, hosts, and their environments [6].

Chagas disease, also known as American trypanosomiasis, is a complex zoonotic NTD caused by the protozoan *Trypanosoma cruzi*. This NTD is transmitted to humans and to more than 150 species of domestic and wild mammals [7] through the contact with infected feces of hematophagous insects known as triatomines and/or by oral transmission [8,9]. Three key features of *T. cruzi* transmission are known to shape the disease distribution and the success of its control. First, while triatomines feed on a vast diversity of vertebrate species, the susceptibility of these species to *T. cruzi* infection varies greatly. Among competent hosts, some reservoir species, most notably dogs and rodents, increase the risk of human infections to such an extent that they can become the targets of control interventions [10–12]. On the other hand, avian species have been shown to be refractory to *T. cruzi* transmission [13,14]. The balance between competent and non-competent hosts can therefore play a role in regulating *T. cruzi* transmission. Second, the within-host dynamics of *T. cruzi* underlies individual heterogeneities in host infectiousness [15,16], with temporal variations as a result of infection timing among competent hosts. After a primary infection, the parasite load in host’s blood increases until the infected individual becomes infectious for susceptible vectors during bloodmeals. This acute stage is typically followed by a long-term chronic phase characterized by a low parasitemia, whereby a host becomes much less infectious [17,18]. Third, the abundance and population structure of triatomine vectors can exhibit strong seasonal variations (e.g., [19,20]) that could interact with density-dependence mechanisms influencing vectorial transmission. Indeed, individual vector feeding frequency has been shown to decrease with vector density as a result of intraspecific interference competition associated with an increase in triatomine biting perception that trigger defensive behavioral responses from their host [14,21–23] that can potentially lead to oral transmission through predation. Characterizing the interplay between the diversity of host species, the within-host dynamics of *T. cruzi*, and the density-dependent mechanisms influencing vector seasonal demography and transmission remains a significant challenge. The integration of these key elements into well empirically parameterized eco-epidemiological models of *T. cruzi* transmission can undoubtedly provide valuable insights into their impacts on the temporal dynamics of the risk of human infection, and the efficiency of potential control interventions.

The Yucatan peninsula has experienced an increasing transmission of vector-borne and zoonotic diseases since the 1960s [24] under the influence of anthropogenic habitat modification and land use changes [25]. The transmission of *T. cruzi* has been extensively studied in this area, where it is known to cause a 1–5% prevalence of Chagas disease in local populations [26], and to display the three main features described above. A recent local comparative analysis indeed showed that *T. cruzi* is infecting the largest number of vertebrate species among all vector-borne pathogens circulating in the wild fauna [27], and diverse host species have been shown to affect vector house infestation [28] and/or to be able to amplify or dilute *T. cruzi* transmission within the host community typically found in a village [14]. Meanwhile, ecological field studies focused on the demography of the local vector species, i.e., *Triatoma dimidiata*, have documented strong seasonal variations in vector abundance with a marked peak during the warm and dry season (March to May) and a typically low intra-domiciliary presence during the rest of the year [19,29–31]. Those empirical studies allowed to design *T. dimidiata* population dynamic models at the house [32] and village [33] scales, which provided key insights into the strategies to be implemented to control the corresponding entomological risks [34,35]. More recently, Flores-Ferrer et al. [14] designed the first eco-epidemiological model of *T. cruzi* transmission through its local host community that explicitly accounted for the impact of triatomines intraspecific competition on both vector demography and parasite transmission. While this model provided key quantitative assessments of the local role of various host species in *T. cruzi* transmission, and of the potential efficacy of zooprophylaxis strategies, it did not take into account the chronic infection status of the hosts and the seasonal variations in vector demography.

In this contribution, we strengthened this modelling of *T. cruzi* transmission in a multi-host community by relaxing those two simplifying assumptions to build an eco-epidemiological model accounting for the infectious status of the hosts and for the interaction between density-dependence mechanisms and seasonality throughout the stage-structured vector life cycle. Such an improved modelling is intended to i) provide the first quantitative description of the local seasonal patterns in *T. cruzi* transmission and its incidence in humans, and to ii) refine our assessment of the role of the various host species in the transmission of *T. cruzi* and of the potential of zooprophylaxis to mitigate disease risk.

## Materials and methods

### General approach

We extended the compartmental Susceptible (S) - Infectious (I) model developed by Flores-Ferrer et al. [14] into an eco-epidemiological model allowing to predict the seasonal variations in i) *T. dimidiata* stage-structured demography and ii) *T. cruzi* transmission through the host community typically encountered in villages of the Yucatan peninsula, Mexico (See ‘Modelling *T. cruzi* transmission in structured hosts and vector populations of the Yucatan peninsula’ below). We adapted our previous modelling by accounting for two additional structures of the vector and hosts populations. First, each infected host population was partitioned into ‘infectious’ and ‘recovered’ (R) individuals corresponding to hosts in the acute and chronic phases of the infection, in order to take into account that the low parasitemia during chronic infection typically reduces *T. cruzi* transmission to susceptible vectors [17,18,36]. Second, the vector population was structured into 7 stages (eggs, nymphs 1–5 and adults) to provide a description of the relationship between *T. dimidiata* demography and its stage-dependent blood-feeding behaviour. We parameterized this SIR eco-epidemiological model by taking advantage of long-term field and experimental studies of the local host-vector-parasite system that included estimates of vector and hosts demographic rates and of their prevalence of infection by *T. cruzi*, along with a detailed metabarcoding description of the triatomine blood-feeding behavior (See ‘Vector feeding preferences’ below). We then used this parameterized modelling to make predictions about the seasonal variations in vector abundance and *T. cruzi* transmission within its local synanthropic and domesticated hosts community, and to assess the potential efficacy of zooprophylactic interventions based on the manipulation of this local host community.

### Modelling *T. cruzi* transmission in structured hosts and vector populations

**Fig 1:**
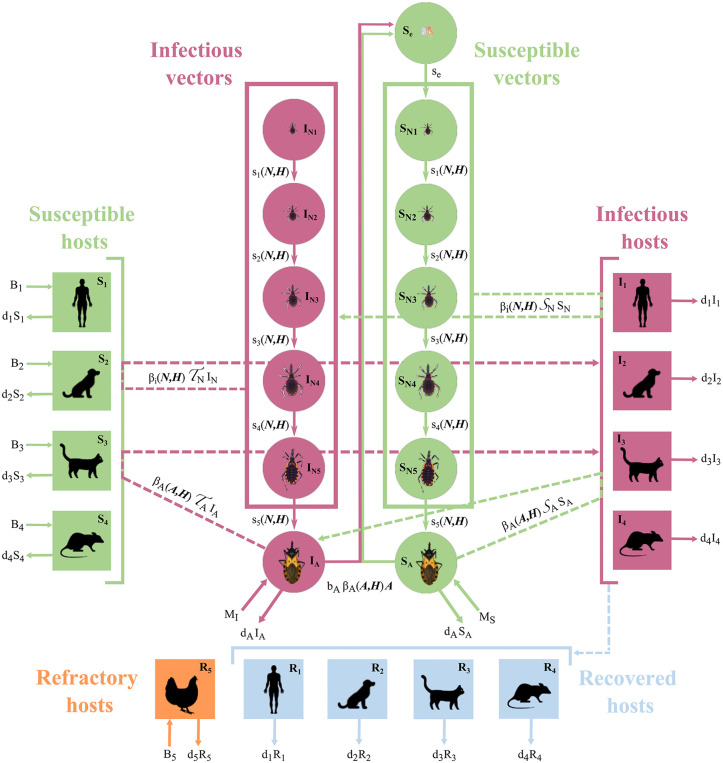
Schematic representation of the SIR model for *T. cruzi* transmission by *T. dimidiata* vectors in a multi-host community. Vectors infected (red) or not (green) by *T. cruzi* can consume blood from humans, dogs, cats, rodents and avian hosts that are susceptible (green), infectious (red), recovered (blue) or refractory (orange) to the parasite infection, with a transition from the infected to recovered status in hosts that takes 5 months. Full arrows then represent the demographic processes of hosts and vectors while dashed arrows correspond to the infection processes. Links to the sources of the black icons used to represent hosts species are available at the end of the reference section.

Following Flores-Ferrer et al. [14], our SIR model of *T. cruzi* transmission (Fig 1) includes four competent hosts; humans (h = 1), dogs (h = 2), cats (h = 3), rodents (h = 4) and non-competent avian hosts (h = 5) that, altogether, account for up to 89% of all bloodmeals typically made by *T. dimidiata* within villages of the Yucatan peninsula [37,38]. For each competent host species, individuals are either susceptible (${\text{S}}_{\text{h}}$), infectious (${\text{I}}_{\text{h}}$) or ‘recovered’ (${\text{R}}_{\text{h}}$), where the latter two categories correspond to the acute and chronic phases of the infection (as in [17]). Refractory hosts (avians) (${\text{R}}_{\text{5}})$ are then classified as recovered in the model as they are not competent to transmit *T. cruzi* to susceptible vectors. All hosts individuals are considered to die at a species-specific per-capita mortality rate d_h_, and such deaths are balanced by the recruitment of susceptible individuals at a constant rate B_h_. The adult vector population is made of susceptible (${\text{S}}_{\text{A}}$) and infectious (${\text{I}}_{\text{A}}$) individuals that die at a natural per-capita mortality rate d_A_, and their death is balanced by the immigration of M_S_ susceptible and M_I_ infected individuals dispersing from the sylvatic habitat and by their reproduction within the village. As in the original modelling [14], adult females produce susceptible eggs according to the amount of ingested blood (e.g., [29]). The fertility of an adult female was then modelled as the product of the fertility per blood meal b_A_ and the per adult vector blood-feeding rate $\beta_{A}\left(A,H\right)$ that accounts for the intraspecific competition between the adult vectors (***A***) aiming to feed on host individuals of all species (***H***). After eggs hatch into first stage nymphs with a constant probability s_e_, each nymphal stage develops into the next-one, and ultimately into adults, according to probabilities s_i_(***N***,***H***) (i = 1…5) that decrease with respect to the competition between nymphs of all stages (***N***) for blood-feeding onto all host species (***H***). In such a stage-structured vector population, both nymphs and adults of *T. dimidiata* can get infected by and transmit *T. cruzi* to any of the competent host species. The *T. cruzi* transmission rates between nymphal and adult vectors and hosts were then set according to stage-specific (i.e., nymphal and adult) vector’s i) blood-feeding rates $\beta_{A}\left(A,H\right)$ and $\beta_{i}\left(N,H\right)$ (i = 1…5), and ii) proportions of bloodmeals made on the different competent host species $\varphi_{Ah\;}\;$and $\varphi_{Nh\;}$ (h = 1…5), while considering distinct (per potentially infectious contact) probabilities of *T. cruzi* transmission from any infectious host to a susceptible vector (*p*_*v*_) and from an infectious vector to a susceptible host h (*p*_*h*_). Following initial infection by *T. cruzi*, susceptible hosts develop an infectious acute phase which typically lasts for about 5 months, as observed from xenodiagnostic data collected on dogs and rodents [18,36] until the decrease of parasitemia to undetectable levels, characterizing the non-infectious chronic phase.

Overall, the above model expands the SI model of *T. cruzi* transmission into a more detailed SIR framework that still obviously relies on simplifying assumptions. Those assumptions typically allow discarding some sources of heterogeneity (within human or other host species, vectors and parasites) to focus on the description of those that are being investigated. In our village scale modelling, we aimed to look at the transmission of *T. cruzi* by stage structured vector *T. dimidata* populations to typical host species while distinguishing between susceptible individuals and those in the acute and chronic phases of infection. Meanwhile, our model does not explicitly account for differences between i) habitats and households (that could potentially lead to heterogeneous infection rates at a smaller scales), ii) vectors and human or non-human hosts behaviour (that can induce heterogeneous rates of encounters) and between iii) strains of parasites (that might exhibit various levels of infectivity), that undoubtedly provide additional source of complexity to *T. cruzi* dynamics at various spatial and temporal scales [39]. In addition, such heterogeneities are usually associated with stochastic variations generating localized microvariations in transmission dynamics that can potentially spread and affect the broader scale epidemiological patterns. It is therefore worth mentioning that outcomes of our deterministic SIR model typically represent average dynamics that do not allow capturing the entire range of trajectories arising from such fine scale stochastic variations in *T. cruzi* transmission and their potential spread through the village transmission community. Finally, the model assumes an infectious phase (I) while it neglects any potential transmission during the chronic phase (R), which is a very standard implicit assumption of most others SI Chagas disease modelling [39]. In a rare attempt at including transmission during a chronic stage of CD, the per contact probability of transmission from hosts in chronic phase to susceptible vectors was estimated to be as low as 4.10^-3^ to 6.10^-3^ [17]. This represents one successful *T. cruzi* transmission per ~200 contacts, which indeed was predicted to be one the parameter with the lowest impact on the spread of *T. cruzi* [17] and supported the idea that such very low potential of transmission could be neglected.

The details of mathematical equations describing all the demographic and transmission processes represented in Fig 1 are provided in S1 Text. The model parameters describing those processes are listed in Table 1 along with their estimates that were derived according to the methodology and data described below.

**Table 1:** Definitions and estimates of the model parameters.

Symbol	Name	Units	Value	References
*Hosts demography*				
*N*_*h*_	Number of host type h	ind		
	*Humans, Dogs, Cats, Rodents, Avians*		2011, 727, 601, 3481, 3471	[40,41]
*B*_*h*_	Recruitment rate of host type h	ind.month^-1^		
	*Humans, Dogs, Cats, Rodents, Avians*		2.394, 20.194, 12.521, 870.25, 578.5	[14,42], This study
*d*_*h*_	Death rate of host type h (10–2)	month^-1^		
	*Humans, Dogs, Cats, Rodents, Avians*		0.119, 2.777, 2.083, 25, 16.666	[14,42], This study
*Vectors demography*				
*M*_*S,*_ *M*_*I*_	Migrating adult vectors	ind.month^-1^		
	*Susceptible, Infected*		45060, 2876	[31,33,37,38]
*p*_*f*_ *(t)*	Proportion of female *T. dimidiata* adults	month^-1^		
	*January, February, March, April, May, June*		0.48, 0.54, 0.62, 0.69, 0.70, 0.58	[38]
	*July, August, September, October, November, December*		0.77, 0.73, 0.70, 0.71, 0.58, 0.55	[38]
*β*_*Amax*_	Maximal vector feeding rate of adults	month^-1^		
	*March, April, May, June, July, August, September - February*		2.705, 5, 5, 3.853, 2.705, 1.558, 0.410	[37,38,43,44], This study
*γ*_*A*_	Density-dependent regulation of adult vectors feeding		0.02	[23]
*b*_*A*_	Vector fertility per bloodmeal		5.399	[29,45]
*s*_*i*_	Survival rate of nymphs type i	month^-1^		
	*Nymph 1, Nymph 2, Nymph 3, Nymph 4, Nymph 5*		0.863, 0.875, 0.869, 0.924, 0.957	[46]
*β*_*imax*_	Maximal vector feeding rates of nymphs type i	month^-1^		
	*Nymph 1, Nymph 2, Nymph 3, Nymph 4, Nymph 5*		1.525, 3.051, 3.313, 2.345, 1.885	[46]
*γ*_*N*_	Density-dependent regulation of nymph vectors feeding		0.0055	This study
*d*_*A*_	Adult vector death rate	month^-1^		
	*March - August, September - February,*		0.65, 0.372	[37,38], This study
*Vectors feeding preferences*				
*ϕ*_*Ah*_	Proportions of adult bloodmeals on host type h			
	*Humans, Dogs, Cats, Rodents, Avians*		0.537, 0.18, 0.029, 0.214, 0.04	[37,38]
*ϕ*_*Nh*_	Proportions of nymph bloodmeals on host type h			
	*Humans, Dogs, Cats, Rodents, Avians*		0.372, 0.163, 0.023, 0.14, 0.302	[37,38]
*α*_*Ah*_	Adult vector feeding rates on host type h			
	*Humans, Dogs, Cats, Rodents, Avians*		2.100, 1.947, 0.379, 0.483, 0.091	[14], This study
*α*_*Nh*_	Nymph vector feeding rates on host type h			
	*Humans, Dogs, Cats, Rodents, Avians*		1.609, 1.951, 0.333, 0.350, 0.757	[14], This study
*Transmission probabilities of T. cruzi*				
*p*_*v*_	Probability of vector infection from infected host		0.95	[17]
*p*_*h*_	Probability of host h infection from vector (10–4)			
	*Humans, Dogs, Cats, Rodents, Avians*		0.00608, 0.595, 2.15, 35.7, 0	This study

### Model parameterization to the *T*. *dimidiata* - *T*. *cruzi* - domesticated and synanthropic host community

#### Hosts demography.

The average size of the human population (N_1_) and number of households encountered in the villages of the Yucatan peninsula, Mexico, i.e., Sudzal, Teya and Bokoba, where the transmission of *T. cruzi* through its local vector and host species has been investigated over the last 20 years, were set to 2011 and 594 according to the last regional census [40]. The abundance of dogs (N_2_), cats (N_3_), rodents (N_4_) and avian (N_5_) hosts were estimated as the product of i) their average per household, that were previously found to be equal to 1.22, 1.01, 5.86 and 5.84 from a field survey performed on 308 houses [41] (Dumonteil, personal communication), and ii) the average number of households. The death rate of each host species h (*d*_*h*_) was calculated as the inverse of its average lifetime expectancy [17], with values for human, dogs, cats, rodents and avian hosts set to 70 years, 3 years, 4 years, 4 months and 6 months, respectively. Such lifetime expectancies were all derived from Flores-Ferrer et al. [14], but for rodents as it was then tailored according to the study by Panti-May et al. [42] on mouse demography. The recruitment rate of host species h (*B*_*h*_) was then estimated by assuming that the number of hosts of each species (N_h_) correspond to its population dynamic equilibrium between births and deaths, which can be shown to be $N_{h}^{*}=\;\frac{B_{h}\;}{d_{h}\;}$, so that B_h_ is merely equals to d_h_*N_h_. All the N_h_, B_h_, and d_h_ estimated values are provided in the sub-section ‘Host demography’ of Table 1.

#### Vectors demography.

The population dynamics of non-domiciliated *T. dimidiata* within the villages of the Yucatan peninsula is shaped by adult dispersal and stage-structured demography typical of hemimetabolous insects. We used its long-standing field and modelling studies to estimate the corresponding parameters as follow.

*Adult dispersal and reproduction*. The abundance of adult vectors dispersing into the village of the Yucatan peninsula shows a strong seasonal pattern with a peak from March to May [47] that has already been quantified [33] along with their *T. cruzi* prevalence [31,37,38], which provided estimates for the number of susceptible (M_S_) and infected (M_I_) migrating adults. To model the reproduction of (migrating and non-immigrating) adults within the village, we accounted for their sex ratio and its monthly variations that were observed from March 2017 to July 2019 in the villages of Teya, Sudzal and Chumbec, by Moo-Millan [38]. Since the monthly proportions of females did not vary significantly from one year to another (χ^2^ = 304.5, df = 297, p = 0.3698) (Fig A in S1 Text), we used their average estimates that confirmed the previously identified female-biased sex-ratio [29], with a proportion of females in the population varying from 48% (± 20%) in January to a maximum of 77% (± 8%) in July. The egg production by *T. dimidiata* females was set as the product of the per adult blood-feeding rate $\beta_{A}(A,H)$ and the fertility per blood meal (b_A_). The former was modelled as in Flores-Ferrer et al. [14] to allow for the maximum per adult blood feeding rate (*β*_*Amax*_*)* to decrease according to the vector to host abundance ratio (***A***/***H***) and the intensity ($\gamma_{A}$) of the vector intraspecific competition for bloodmeals (See Appendix 1). The maximum per adult vector blood-feeding rate (*β*_*Amax*_) was considered to correspond to the maximum number of different host DNA observed in metabarcoding studies of the adult vector gut content [37,38]. As a matter of fact, host DNA are known to remain detectable for 30 days in the digestive system of triatomine vectors [43], a duration which correspond to the time step of our model, so that the maximum number of detected meals provided a sensible estimate of the *β*_*Amax*_ equals to 5. This estimate was then adjusted to account for its expected monthly variations with respect to the seasonal patterns previously observed by Catalá [44]. Meanwhile, the intensity of intraspecific competition ${(\gamma }_{A}$) was set to 0.02 so that the adult blood feeding rate is halved for a vector to host abundance ratio (***A***/***H***) of 50:1, as previously shown by Pelosse et al. [23]. The fertility per blood meal (b_A_) was calculated using data from Zeledón et al. [45] showing that *T. dimidiata* females lay on average 1088 eggs over a 726 days period. Considering the experimentally observed *T. dimidiata* maximum blood-feeding rate of ~5 meals per month [37,38], the fertility per blood meal can be estimated to be 1088/(5*(726/30)) = 9 eggs per meal under laboratory conditions, in lines with estimates from Montes de Oca-Aguilar et al. [48] for various *T. dimidiata* populations in Yucatán. This value was further reduced according to a previous estimate of a 40% reduction in *T. dimidiata* fertility in the field as compared to its value observed in laboratory colonies, which was found to be highly consistent with a 41% reduction in blood volume consumed by insects collected in villages as compared to laboratory-raised females [29].

*Stage-specific development and mortality rates*. The duration of eggs and nymphal stages were set according to *T. dimidiata* cohort studies ([49], p.65), where the egg and first nymphal stages were found to last for ~1 month, while the second to fourth nymphal stages lasted ~2 months and the last stage took ~3 months before individuals become adults. The probability of successful development for each nymphal stage (s_i_) were set to their maximum values derived in laboratory conditions by Nogueda-Torres et al. [46], which are reported in Table 1, and adjusted with respect to their stage specific blood-feeding rate $\beta_{i}\left(N,H\right)$ as those are known to influence survival and development of nymphs [49,50]. The stage specific blood-feeding rates were then modeled using the same density-dependent function as we used for the adults. The value of the intensity of competitive interactions between nymphs for access to hosts ${(\gamma }_{N}$) was indirectly estimated for the modelled adult vector population size to reach 800000 individuals, as predicted by Barbu et al. [33] for the village of Teya. This led to an estimate of $\gamma_{N}$ = 0.0055 corresponding to a ~ 4 times lower intensity of competition than for adults, with a reduction by half of the maximal feeding rate for a vector to host abundance ratio of 182:1. The adult mortality was assessed by using insect field collection conducted over a 3-year study [37,38] that showed their monthly death rate to be higher from March to August, i.e. d_A_ = 0.65, than from September to February, i.e. d_A_ = 0.372. Such estimates correspond to an adult life expectancy of ~1.5 and ~3 months, respectively, which are very close to the previous 2.5 month’s estimate derived from the same vector populations of the Yucatan peninsula [32].

#### Vectors feeding preferences.

We estimated the blood-feeding rates made by adults (α_Ah_) and nymphs (α_Nh_) on all host types h (h = 1…5) using a meta-analysis of *T. dimidiata* bloodmeal metabarcoding studies achieved in villages of the Yucatan peninsula over the last 10 years [37,38], and the procedure developed and implemented in Flores-Ferrer et al. [14]. The meta-analysis provided an estimate of the proportion of bloodmeals taken by infected and non-infected adults and nymphs in both domiciles and backyards (Table A in S1 Text). Since ~95% of the 243 collected adults were found in domiciles where no significant feeding differences between infected and non-infected individuals were observed (χ^2^ = 3.443, df = 4, p = 0.487), we ultimately pooled those data to estimate the average proportions φ_Ah_ of adult bloodmeals made on host types h (h = 1…5). Adults vectors typically fed on humans, rodents and dogs, representing 54%, 21% and 18% of bloodmeals, while avian hosts and cats accounted for 4% and 3% of bloodmeals, respectively. The total number of collected nymphs was much lower, i.e., 27, with ~82% of them found in the backyards and none being infected. Therefore, we again pooled the data to estimate the average proportions φ_Nh_ of nymphal bloodmeals made on host types h (h = 1…5). Nymphs fed less on humans, rodents, dogs and cats, representing only 37%, 16%, 14% and 2% of bloodmeals, while more than 30% of their bloodmeals were taken on avian hosts. Those differences between the composition of adults and nymphs bloodmeals (χ^2^ = 43.217, df = 4, p = 9e-09) are clearly associated with their spatial partition between houses and backyards. From those estimates of the proportions of bloodmeals taken by adult ($\varphi_{Ah\;}$) and nymphs ($\varphi_{Nh\;}$) on host types h (h = 1…5), we derived the vector blood-feeding rates of adults (α_Ah_) and nymphs (α_Nh_) following Flores-Ferrer et al. [14] to account for the impact of the host populations sizes (N_h_) on the observed composition of bloodmeals.

#### Transmission probabilities of *T*. *cruzi.*

The probability of *T*. *cruzi* transmission from an infected host to a susceptible vector (*p*_*v*_) was set according to its previous estimate by Rascalou et al. [17]. The probabilities of transmission from infectious vectors to each of the competent host species were estimated to match the mean prevalences observed in those species in the Yucatan peninsula. To do so, these per (infectious vector) contact transmission probabilities of *T. cruzi* to each of the 4 competent hosts were varied systematically (from 0 to 1). For each tested set of 4 probability values (p_1_, p_2_, p_3_, p_4_), the mean absolute deviation between the observed and predicted prevalences was calculated over a yearly time window after the asymptotic dynamics has been reached. The quadruplet of values minimizing the mean absolute deviation provided the estimates of the per contact probabilities of transmission of *T. cruzi* from an infectious vector to each of the 4 competent host species. The observed prevalences of infection were taken from previous field studies located in villages of the Yucatan peninsula, where they were found to be 2.3% in humans (9/390) [51], 9.8% in dogs (10/102) [52], 8.6% in cats (19/220) [53] and 9.9% in rodents (57/575) [54]. The corresponding values of the host to vector probabilities of transmission (*p*_*h*_) were found to be very similar with their previous estimates [14] and allowed to complete Table 1 and the parameterization of our model.

### Analysis of the seasonal vector demography and *T*. *cruzi* transmission dynamics

**Fig 2:**
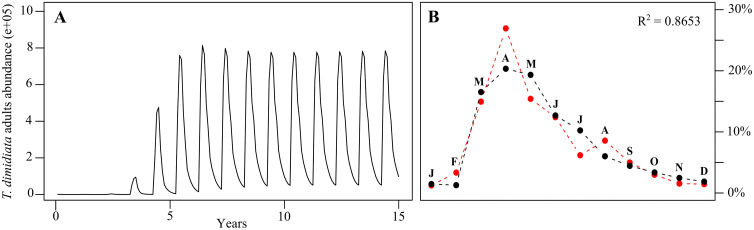
Seasonal variations of *T. dimidiata* abundance in a village of the Yucatan peninsula. The predicted temporal variations of the number of *T. dimidiata* adults are represented in black for a 15-years period **(A)**. The monthly abundance of adults predicted by our modelling (black) and observed by Moo-Millan et al. [31] (red) were found to be highly correlated **(B)**.

**Fig 3:**
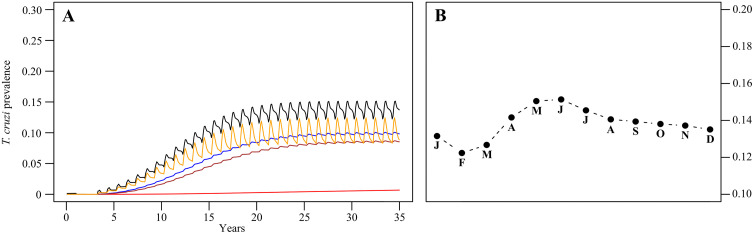
Seasonal variations of *T. cruzi* host and vector prevalences in a village of the Yucatan peninsula. The seasonal variations in the prevalence of infection by *T. cruzi* are represented for humans (red), dogs (blue), cats (brown) and rodents (orange) during a 35-year period **(A)**, and for *T. dimidiata* adults (black) during a 35-year (A) and a 1-year period **(B)**.

**Fig 4:**
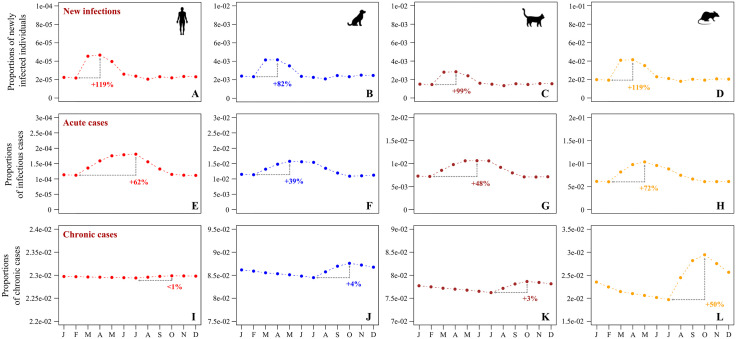
Seasonal variations of *T. cruzi* incidence and acute-chronic cases in a village of the Yucatan peninsula. The monthly variations in the proportions of newly infected individuals **(A-D)**, infectious (E-H) and chronic (I-L) cases measured among the entire population of humans (red), dogs (blue), cats (brown), rodents (orange) are represented for a 1-year period. Links to the sources of the black icons used to represent hosts species are available in S1 File.

**Fig 5:**
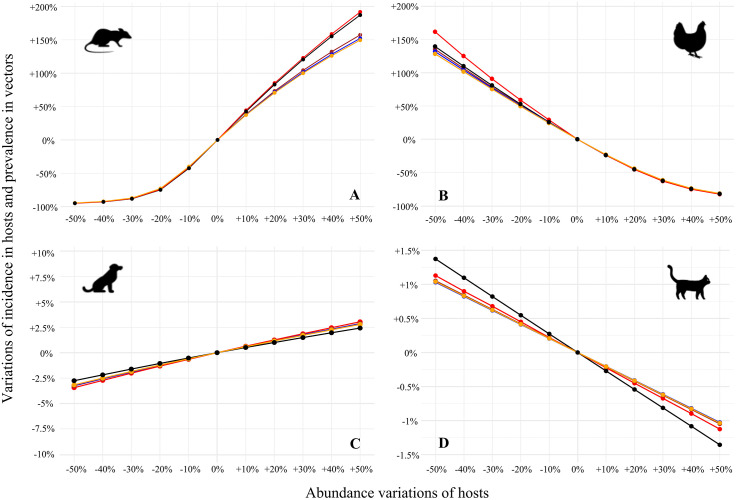
Predicted impact of host community composition on *T. cruzi* transmission. Variations of annual incidence in humans (red), dogs (blue), cats (brown), rodents (orange) and prevalence of infection in *T. dimidiata* (black) were predicted while varying the abundance of rodents **(A)**, avian species **(B)**, dogs (C) and cats (D) from -50% to +50%. Links to the sources of the black icons used to represent hosts species are available in S1 File.

**Fig 6:**
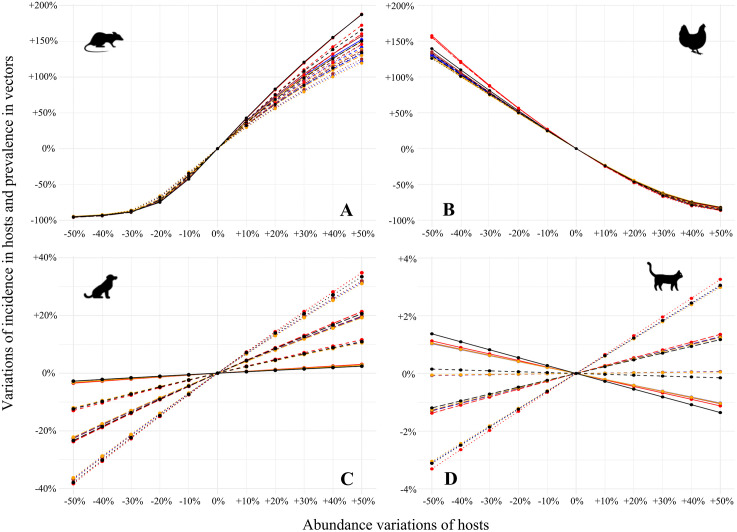
Predicted effect of the host community composition and the duration of *T. cruzi* acute infections on its transmission. Variations of annual incidence in humans (red), dogs (blue), cats (brown), rodents (orange) and in the prevalence of infection in *T. dimidiata* (black) were predicted by varying the abundance of rodents **(A)**, avian species **(B)**, dogs (C) and cats (D) from -50% to +50% and by increasing the duration of the acute phase from 5 (solid) to 8 (dashed), 12 (long-dash) and 18 (dotted) months. Links to the sources of the black icons used to represent hosts species are available in S1 File.

We first used our well empirically parameterized model to predict the monthly variations in *T. dimidiata* abundance (Fig 2) and *T. cruzi* transmission (Fig 3) within the modelled village. Those former predictions were compared to the monthly variations in vector abundance observed in Teya and Sudzal [31] by calculating the coefficient of determination (R^2^) between predicted and observed within year temporal distribution of vectors. We then investigated the impact of these monthly variations of *T. dimidiata* abundance on parasite transmission by predicting, for each host, the typical seasonal changes in the number of new infections, infected and recovered individuals (Fig 4). We further used our parameterized modelling to simulate variations in the abundance of dogs, cats, rodents and avian hosts in the modelled village within a range of -50% to +50% around the estimated values shown in Table 1, and we assessed the resulting changes in *T*. *cruzi* prevalence of infection among the vector and competent hosts (Fig 5). Finally, we tested the sensitivity of our predictions about the effects of the host community structure to the duration of the host infectious stage as it has been shown that, depending on *T. cruzi* strain, the parasite load can stay high in host blood for a duration exceeding 5 months [18]. We therefore increased the duration of the acute phase from 5 to 8, 12 and 18 months and re-assessed the role of each host species on *T. cruzi* transmission in all these putative epidemiological conditions (Fig 6).

## Results

### Seasonal dynamics of *T. dimidiata* in a village of the Yucatan peninsula

Our parameterized model integrating the observed within-year variations in adult reproduction and survival predicted a strong and stable seasonal pattern of *T. dimidiata* abundance (Fig 2A), with a peak of adult vectors in the March-May period.

The predicted seasonal pattern consistently matched the observed monthly distribution of *T. dimidiata* adult captures in the villages of Teya and Sudzal (Fig 2B) as it properly reproduced the peak of abundance associated with the warm and dry season (from March to May) as well as the observed decrease in vector abundance during the rainy and northerlies season (from June to February). Interestingly, these variations in vector abundance are expected to translate into changes in their (density-dependent) feeding frequency, which our model was also able to predict. The adult feeding frequency was shown to vary from 1.59 to 2.17 bloodmeals per month during the dry season (March to May) and then to slowly decrease until they consume only 0.25 to 0.33 bloodmeals per month during the rainy and northerlies season. These temporal variations in the number of *T. dimidiata* adults and their frequency of bloodmeals are likely to affect *T. cruzi* transmission to competent hosts. The SIR model was then used to look at the potential seasonal variations in *T. cruzi* prevalence in its vector and host community.

### Seasonal dynamics of *T*. *cruzi* transmission in a village of the Yucatan peninsula

The SIR model, parameterized to fit the average prevalence of *T. cruzi* infection observed in the *T. dimidiata* population and the host community, then provided quantitative predictions of the existence of seasonal variations in the infection of vectors and competent hosts (Fig 3A).

Interestingly, the *T. cruzi* infection prevalence in vectors was predicted to vary substantially throughout the year (Fig 3A, 3B), with a 23% increase from March to June followed by a slow return to its initial level during the rainy and northerlies season. Although such variations of *T. cruzi* prevalence were also predicted in the different competent hosts (Fig 3A), as expected, the corresponding oscillations were of smaller amplitude for hosts with typically long-life expectancies. In fact, rodent prevalence rose by 50% between February and May, while, during the same period, prevalence of infection only increased by 4%, 3% and <1% in dogs, cats and humans, respectively. The increase in triatomine abondance and the resulting variations in *T. cruzi* prevalences throughout the year, clearly called for additional predictions to identify periods with a high risk of transmission, i.e., months of the year when the incidence (number of new cases) is expected to be higher.

### Seasonal variations in the incidence of *T. cruzi* infection, and in the number of acute and chronic cases

Our SIR model predicted that the warm and dry season (March to May) represents a crucial period for *T. cruzi* transmission with a high risk of infection for all the competent hosts (Fig 4A-4D) since 39% of the yearly new infections (across all hosts) occur during these 3 months.

The number of new infections varied importantly between hosts, with 0.68, 24, 13, 1042 cases per year for humans, dogs, cats and rodents, respectively, and their seasonal increase during the warm and dry season was predicted to be more pronounced for humans and rodents (+119%) than for cats (+99%) and dogs (+82%) (Fig 4A-4D). Following the transmission of *T. cruzi*, these new infected individuals enter an acute phase where the parasite load quickly increases in the host blood. As expected, given the 5-months duration of this acute phase, the peak of acute cases in each host type was broader and lasted until October, when the proportion of such cases returns to initials levels (Fig 4E-4H). The rise in the proportion of acute cases among hosts mimicked the seasonal pattern observed for new infections in humans (+62%), rodents (+72%), dogs (+39%) and cats (+48%) (Fig 4E-4H), albeit at slightly lower rates according to their broader seasonal distribution. Finally, as the density of *T. cruzi* in their blood decreases, the hosts enter a non-infectious chronic phase. The monthly variations in the proportion of individuals in this chronic stage were even lower as they included infections of the previous years, but in the short-lived rodents where an increase of 50% of chronic cases was predicted between July and October (Fig 4I-4L).

The between-hosts differences in the incidence rate and in the seasonal variations of acute cases, which represents the main sources of infection for susceptible vectors, led us to investigate how variations in the abundance of each host type affect *T. cruzi* transmission.

### Impact of the host community composition on *T. cruzi* transmission

The analysis of the host community composition clearly shows that the abundance of the highly competent rodent hosts has a strong impact on the transmission of *T. cruzi* to the entire community (Fig 5A). Increasing the rodent population size by 50%, made *T. cruzi* prevalence in *T. dimidiata* vector and the number of new infections in human to increase by 187%, while its incidence in rodents, dogs and cats were predicted to increase by 150%, 152% and 157%, respectively. Meanwhile, reducing the abundance of rodents present in the village by 50%, led the parasite prevalence in vectors and its incidence in all competent hosts to decreased by ~95%.

The variations in the abundance of non-competent avian hosts also had a significant, albeit opposite effect on *T. cruzi* transmission (Fig 5B). An increase in the avian host population size of 50% induced a reduction in the *T. cruzi* prevalence in vectors of 82% and diminutions of its incidence in all hosts of ~83%. Meanwhile, a 50% reduction in the avian host population size led to a 140% larger *T. cruzi* prevalence in vectors and to increases of its incidence in humans, dogs, cats and rodents of 158%, 132%, 135% and 129%, respectively. Finally, the abundance of dogs and cats were shown to have much lower effects on *T. cruzi* transmission since varying their abundance by 50% only made its prevalence in vectors and incidence in hosts to change by ± 2.5-3% and ± 1-1.4%, respectively (Fig 5C,5D).

While the significant effects of rodent and avian populations on *T. cruzi* transmission indicate that strategies based on their control could be efficient, we aimed at testing the sensitivity of those predictions to the duration of the acute phase in hosts as variations have been observed between *T. cruzi* strains [18].

### Sensitivity analysis to the duration of *T. cruzi* acute infection in hosts

The sensitivity analysis on the duration of the acute *T. cruzi* infection phase in competent hosts had little effect on the contribution of rodent and avian hosts on the overall transmission of the parasite (Fig 6A,6B). The changes in the population size of rodent and avian hosts were indeed predicted to have similarly important effects on *T. cruzi* vector prevalence and annual incidence in hosts when the duration of the acute phase was progressively increased from 5 to 18 months (Fig 6A,6B).

Meanwhile, a longer acute and infectious phase in dogs significantly increased their impact on *T. cruzi* transmission as the effects of changing the dog population size on *T. cruzi* prevalence in vectors and incidence in hosts went up from ± 2.5-3% (for a 5-months acute phase) to variations in human incidence that ranged from -38% to +35% (for a 18-months acute phase) (Fig 6C). The impact of the duration of the acute phase in cats was much lower, although it had an unanticipated effect: increasing the duration of the infectious acute phase made this competent host contributing more efficiently to transmission to an extent that increasing its density went from having a diluting to an amplifying effect on *T. cruzi* transmission, which nonetheless remains lower than 3% in all hosts, even for an 18-months acute phase (Fig 6D).

## Discussion

In this contribution, we took advantage of the long-standing field and modelling studies of the transmission of *T. cruzi* in the Yucatan peninsula, Mexico, to build and parameterize an original SIR model of vector-borne transmission in a multi-host community that account for the within-year variations in *T. dimidiata* stage-structured demography and feeding behaviour. This integrative eco-epidemiological modelling provides the first quantitative insights into the seasonal dynamics of *T. dimidiata* abundance and blood-feeding frequency, and their impact on the seasonal pattern of *T. cruzi* incidence in humans and in its domesticated and synanthropic hosts, thereby improving the key understanding required for the design of efficient control strategies.

The first key outcome of our study is that by accounting for the *T. dimidiata* stage-specific demography, feeding preferences and competitive interactions and by considering their within-year variations, our modelling successfully reproduced 86.5% of the seasonal variations in vector abundance typically observed in villages of the Yucatan peninsula. These predictions were derived under demographic and feeding conditions corresponding to our current understanding and estimates of the determinants of this non-domiciliated vector population dynamics. The model, parameterized from locally acquired demographic [31] and blood-feeding [37,38] data, showed that the percentage of adult insects in the village is expected to vary from 2% to 37% during the year with a peak of abundance in April, as already anticipated for the same villages of the Yucatan peninsula [32–35]. The percentage of adult triatomines found in the village after that they migrated from the sylvatic habitat was then calculated to be on average 6.1% of insects, which is consistent with the outcomes of previous genetic and modeling studies where this proportion lied between 5% and 16% [14,55]. The model concomitantly predicted the triatomine (density-dependent) blood-feeding frequency to range from 1.58 to 2.16 meals per month during the warm and dry season, which was also found to be highly consistent with its observed value estimated to range between 1.27 and 1.67 bloodmeals from metabarcoding studies [37,38]. Triatomine blood-feeding frequency is then predicted to slowly decrease until they consume only 0.25 to 0.33 bloodmeals per month during the rainy and northerlies season, which roughly corresponds to the maximal amount of time between bloodmeals, i.e., 3–4 months, that is required for survival [49]. Although our estimate of the maximum per adult vector blood-feeding rate (*βAmax*) is consistent with previous studies [23], the metabarcoding assessment allows to discriminate distinct genotypes (from the same or from different host species) but not to quantify the number of blood meals taken on the same host individual over a 30-day period. Although this limitation could potentially lead to underestimate the maximal feeding frequency of highly domesticated vector species, it is much less likely to bias such an estimate for non-domiciliated *T. dimidata* populations that consistently tend to disperse from one house to another in the Yucatan peninsula [56]. Still, a larger estimate of *βAmax* would not affect our model predictions as other parameters ($\gamma_{N},p_{h}$) were concomitantly set to allow for vector abundance and *T. cruzi* prevalence in hosts to reach their observed values. Overall, even if we relied on fixed point estimates for parameters whose seasonal variations were not documented in the field, which could potentially impact the precision of the model predictions, our vector stage-structured model was still able to predict the local seasonal variations in their abundance according to their (density-dependent) blood-feeding mediated development and reproduction, which allowed us to further look at their impact on *T. cruzi* transmission. Our stage-structured model parameterized by integrating the available data on *T. dimidiata* was therefore able to predict the local seasonal variations in vector abundance according to their (density-dependent) blood-feeding mediated development and reproduction, which allowed to look at their impact on *T. cruzi* transmission.

The second main outcome of our integrative modelling is that it allowed for the first quantitative predictions about the seasonal variations in *T. cruzi* transmission. The prevalence of *T. dimidiata* infection by *T. cruzi* was predicted to increase by 23% from March to June in the Yucatan peninsula, followed by a slow return to its initial level during the rainy and northerlies season. This predicted pattern was found to be robust to changes in both transmission and demographic parameters (Figs B-F in S1 Text), and it was further confirmed by re-analyzing the field estimates of *T. dimidiata* infection prevalence that indeed show a 19% increase during the warm and dry season (March to May) [31]. Meanwhile, the prevalence of *T. dimidiata* infection by *T. cruzi* infection was predicted to vary between 12.5% and 15.4%, which belong to the 95% interval estimate derived from local field studies [31]. While these seasonal variations led to a similar pattern in rodents, with a 50% increase in their prevalence of infections by *T. cruzi*, such changes remained lower than 5% in dogs, cats, humans and rodents, which is merely explained by their longer lifetime. This, however, can be deceptive as we actually predicted a peak of new infections spread across a three-months period, from March to May, when *T. cruzi* incidence increased by 82% and 99% in dogs and cats, and by 119% in humans and rodents. This first quantification of a period of higher risk of *T. cruzi* transmission in the Yucatan peninsula, which led to 39% of all the annual new infections by *T. cruzi*, closely matched the warm and dry season, as already observed elsewhere from human’s incidence epidemiological data [57,58]. Interestingly, this 3-months increase in the incidence of new cases turned into a 7-months period, from March to September, characterized by a significant increase in the proportion of hosts in the 5-months long acute phase of the disease. Overall, the number of new infections predicted for our typical village varied significantly between hosts with 0.68, 24, 13 and 1042 annual new cases among ~2000 humans, ~ 730 dogs, ~ 600 cats and ~3480 rodents, respectively, with the predicted incidence in humans being relatively closed to the epidemiological records of 0.7 new infection per year in humans ([51], Cruz-Chan, personal communication). Interestingly, while the model predicted significant seasonal variations in the incidence of *T. cruzi* and in the number of acute cases in every host species, the number of chronic cases remained roughly constant, except in short-lived rodents. This lack of temporal changes then does not allow the current epidemiological monitoring, classically implemented through the detection of antibodies, to detect the predicted seasonal pattern of infection in humans, domestic and synanthropic hosts. These new results clearly states the interest of focusing the prevention and detection of acute cases on risky warm and dry season, which could help both significantly reducing *T. cruzi* transmission and providing a better patient care through adapted treatment that are thought to considerably reduce the subsequent development of chronic disease [59].

The third key outcome of our SIR modeling is to provide further evidence that avian, rodents and dogs all play a significant role in *T. cruzi* transmission through the host community typically encountered in villages of the Yucatan peninsula, so that their management could be part of zooprophylactic control strategies. Under the model settings, an increase in avian species abundance indeed led to a nearly linear reduction of the infection risk in the village, with an about ~80% reduction of *T. cruzi* prevalence in vectors and incidence in all competent host species when the abundance of avians was increased by up to 50%. Furthermore, any 1% increase in the number of avian hosts could induce a ~ 1.6% reduction in incidence which is slightly higher but remains consistent with the 0.45% reduction in prevalence predicted in our previous modelling [14]. Such predictions support that, while avian hosts are important blood source for triatomines, and therefore constitute a key risk factor of house infestation by the studied *T. dimidiata* populations, they actually are refractory hosts that overall contribute to dilute the transmission of *T. cruzi* in the Yucatan peninsula [14]. Interestingly, a similar dilution effect was also observed for *T. infestans* as avian hosts were shown to lower *T. cruzi* prevalence in humans [60] while increasing household’s infestation [61,62]. Finally, it is worth pointing out that our model has been calibrated on a local community and that the generality of its outcomes obviously remains to be tested. Meanwhile, the support that it provides to a promising potential for the management of (peri-)domestic abundance of avian species as part of a cost-effective vector control in the Yucatan peninsula is an encouraging perspective that will need to be validated by empirical field studies before the implementation of such zooprophylactic strategies. Meanwhile, rodent species were also predicted to play a significant role in the local transmission of *T. cruzi*, with a decrease of 50% of their abundance in the village reducing the parasite prevalence in vectors and its incidence in competent hosts by ~95%. While such effects are consistent with empirical studies that have provided evidence of a correlation between the presence of rodents and the transmission of the parasite [12,63,64], they turned out to be higher than previously anticipated [14]. Such an increase in the predicted effect of rodents is readily linked to the outcomes of recent metabarcoding analyses that provided a better knowledge about the host species used by *T. dimidiata* as bloodmeal sources in the studied villages [38]. The latter studies ran on over 243 vector individuals indeed showed that, among bloodmeals made on rodents, most were taken on *Mus musculus*, that represented 88% of adult and 80% of nymph bloodmeals, and on *Rattus rattus*, that were found to account for 20% of the nymph bloodmeals. The tailoring of our model to these two important rodent species, led to consider a shorter rodent life-expectancy and a higher feeding preference by *T. dimidiata* (4.vs. 24 months, 0.21.vs. 0.07 in [14,17]). While the former reduced the prevalence of *T. cruzi* in those hosts and their role in transmission, the latter had an opposite and prominent effect (See Figs B, C in S1 Text), which led to the overall increase in the predicted role of rodents. The prevalence of infection in rodents was indeed estimated to be ~ 9.9% [54] and the effects of those hosts were shown to increase as compared to previous local modelling, which actually made them more consistent with alternative assessment led on places where *T. dimidiata* is the main vector [12,63] or not [64,65]. Interestingly, in such a context, a community-based intervention conducted in Guatemala showed that a 20% reduction in rodent abundance could be achieved at a village scale and provided a 78% reduction in vector infection prevalence [12], which closely matches the 75% reduction that our model predicted when a similar control of the host community was intended. The metabarcoding data used to parameterize the model showed no significant within-year variation in the composition of *T. dimidiata* bloodmeals despite of rodent populations of the Yucatan peninsula being shown to fluctuate seasonally with higher abundance observed during the dry season [42]. While, accordingly, we did not account for temporal changes in the vector rate of *T. cruzi* transmission to each host species, additional assessments of the seasonality in host abundances and related vector feeding patterns would undoubtedly benefit our understanding of Chagas disease transmission risk. We cannot indeed rule out that an increase in the abundance of rodents, and therefore of their presence in *T. dimidiata* blood meals during the dry season in Yucatan, would result in a higher incidence of *T. cruzi* infection. Lastly, the role of dog abundance was found to be strongly dependent upon the duration of the acute and infectious stage of the disease. Remarkably, the effect of dogs on *T. cruzi* prevalence in vectors and incidence in other hosts went from ± 2.5-3% (for a 5-months acute phase) to ± 35–38% (for a 18-months acute phase) when their abundance was increased by 50%. Although most of our predictions are consistent with previous models that have been designed to investigate the role of these hosts in *T. cruzi* transmission, those typically rely on a simpler partition of their population into two categories; susceptible and infectious individuals. However, such SI models predict that changing the abundance of dogs is likely to have a significant impact on *T. cruzi* prevalence in vectors and hosts [14,66]. By modelling both the acute and the chronic stage of the infection, we showed that the corresponding reduction of the duration of the infectious period can induce a significant reduction in the transmission dynamics of *T. cruzi*, to such an extent that when the duration of the acute stage is reduced to 1–2 months the *T. cruzi* prevalence in *T. dimidiata* is predicted to be 43% lower than observed in the Yucatan peninsula. Meanwhile, the sensitivity analysis that we conducted provided evidence that increasing the duration of the infectious period, effectively making our SIR model converges back to an SI model, allowed to reproduce the important role of dogs identified in our previous modeling [14] while it had not impact on the predictions of the significant contributions of avian and rodent hosts to the overall transmission dynamics of *T. cruzi* in the studied villages. The latter outcome clearly unravels that by artificially expanding the duration of the infectious stage to the host lifetime, SI models are likely to have over-estimated the contributions of hosts whose life expectancy significantly exceed the duration of the acute stage, typically dogs, while we expect less (or no) effects for short-lived hosts, such as rodents. Since it has been shown that various *T. cruzi* strains can exhibit different durations of the acute infectious phase in hosts [18,67], our results suggest that predicting the role of long-lived host species, and the outcome of managing their abundance through zooprophylactic strategies, could be sensitive to the circulating strains of parasites. Overall, the outcomes of this integrative study show that the size of avian, rodent and dog populations in villages can all have significant impact on *T. cruzi* transmission, thereby confirming that the management of the host community provides key opportunities to control Chagas disease in the Yucatan peninsula, Mexico. This strongly supports the implementation of educational and empowerment strategies embedded in One Health approach to sustainably interrupt the ongoing intra-domiciliary vector transmission of *T. cruzi* by *T. dimidiata*.

## Supporting information

S1 Text Mathematical modelling of *T. cruzi* transmission within its host community. **Fig A in S1 Text: Monthly variations of *T. dimidiata* female proportion in villages.** The proportions of female *T. dimidiata* vectors observed from insect captures conducted in 2017 (circle), 2018 (triangle) and 2019 (square), and their average (red) were estimated for each month. **Table A in S1 Text: Nymph and adult vectors feeding preferences.** The percentage of bloodmeals of *T. dimidiata* adults or nymphs, infected or not by *T. cruzi* and collected in houses or backyards are attributed to the 5 main hosts observed through metabarcoding analyses. **Fig B in S1 Text: Seasonal variations of *T. cruzi* vector prevalence and host prevalences (A), incidences and acute-chronic cases (B) for a rodent life-expectancy of 24 months.** The seasonal variations in the prevalence of infection by *T. cruzi*, in the incidence of infection (number of newly infected individuals), and the number of infectious and chronic cases are represented for humans (red), dogs (blue), cats (brown) and rodents (orange) and for *T. dimidiata* adults (black) for a 35 years-period. Links to the sources of the black icons used to represent hosts species are available in S1 File. **Fig C in S1 Text: Seasonal variations of *T. cruzi* vector prevalence and host prevalences (A), incidences and acute-chronic cases (B) for a proportion of 7% of *T. dimidiata* adult bloodmeals made on rodents.** The seasonal variations in the prevalence of infection by *T. cruzi*, in the incidence of infection (number of newly infected individuals), and the number of infectious and chronic cases are represented for humans (red), dogs (blue), cats (brown) and rodents (orange) and for *T. dimidiata* adults (black) for a 35 years-period. Links to the sources of the black icons used to represent hosts species are available in S1 File. **Fig D in S1 Text: Seasonal variations of *T. cruzi* vector prevalence and host prevalences (A), incidences and acute-chronic cases (B) for a duration of the acute (and infectious) phase of 8 months.** The seasonal variations in the prevalence of infection by *T. cruzi*, in the incidence of infection (number of newly infected individuals), and the number of infectious and chronic cases are represented for humans (red), dogs (blue), cats (brown) and rodents (orange) and for *T. dimidiata* adults (black) for a 35 years-period. Links to the sources of the black icons used to represent hosts species are available in S1 File. **Fig E in S1 Text: Seasonal variations of *T. cruzi* vector prevalence and host prevalences (A), incidences and acute-chronic cases (B) for a duration of the acute (and infectious) phase of 12 months.** The seasonal variations in the prevalence of infection by *T. cruzi*, in the incidence of infection (number of newly infected individuals), and the number of infectious and chronic cases are represented for humans (red), dogs (blue), cats (brown) and rodents (orange) and for *T. dimidiata* adults (black) for a 35 years-period. Links to the sources of the black icons used to represent hosts species are available in S1 File. **Fig F in S1 Text: Seasonal variations of *T. cruzi* vector prevalence and host prevalences (A), incidences and acute-chronic cases (B) for a duration of the acute (and infectious) phase of 18 months.** The seasonal variations in the prevalence of infection by *T. cruzi*, in the incidence of infection (number of newly infected individuals), and the number of infectious and chronic cases are represented for humans (red), dogs (blue), cats (brown) and rodents (orange) and for *T. dimidiata* adults (black) for a 35 years-period. Links to the sources of the black icons used to represent hosts species are available in S1 File.
(DOCX)

S1 File Icons. (DOCX)

## References

[1] Wilson AL, Courtenay O, Kelly-Hope LA, Scott TW, Takken W, Torr SJ, et al. The importance of vector control for the control and elimination of vector-borne diseases. PLoS Negl Trop Dis. 2020;14(1):e0007831. doi: 10.1371/journal.pntd.0007831 31945061 PMC6964823

[2] WHO. Vector-borne diseases. 2024. https://www.who.int/news-room/fact-sheets/detail/vector-borne-diseases

[3] WHO. Neglected tropical diseases. 2025. https://www.who.int/news-room/questions-and-answers/item/neglected-tropical-diseases

[4] Lin Y, Fang K, Zheng Y, Wang H-L, Wu J. Global burden and trends of neglected tropical diseases from 1990 to 2019. J Travel Med. 2022;29(3):taac031. doi: 10.1093/jtm/taac031 35238925

[5] WHO. Ending NTDs: Together towards 2030. New road map for neglected tropical diseases 2021-2030. 2025. https://www.who.int/teams/control-of-neglected-tropical-diseases/ending-ntds-together-towards-2030

[6] Chala B, Hamde F. Emerging and Re-emerging Vector-Borne Infectious Diseases and the Challenges for Control: A Review. Front Public Health. 2021;9:715759. doi: 10.3389/fpubh.2021.715759 34676194 PMC8524040

[7] Rassi A Jr, Rassi A, Marin-Neto JA. Chagas disease. Lancet. 2010;375(9723):1388–402. doi: 10.1016/S0140-6736(10)60061-X 20399979

[8] Coura JR. The main sceneries of Chagas disease transmission. The vectors, blood and oral transmissions--a comprehensive review. Mem Inst Oswaldo Cruz. 2015;110(3):277–82. doi: 10.1590/0074-0276140362 25466622 PMC4489464

[9] Prata A. Clinical and epidemiological aspects of Chagas disease. Lancet Infect Dis. 2001;1(2):92–100. doi: 10.1016/S1473-3099(01)00065-2 11871482

[10] Reithinger R, Ceballos L, Stariolo R, Davies CR, Gürtler RE. Chagas disease control: deltamethrin-treated collars reduce Triatoma infestans feeding success on dogs. Trans R Soc Trop Med Hyg. 2005;99(7):502–8. doi: 10.1016/j.trstmh.2004.11.013 15869774

[11] Arce-Fonseca M, Carbajal-Hernández AC, Lozano-Camacho M, Carrillo-Sánchez SDC, Roldán F-J, Aranda-Fraustro A, et al. DNA Vaccine Treatment in Dogs Experimentally Infected with Trypanosoma cruzi. J Immunol Res. 2020;2020:9794575. doi: 10.1155/2020/9794575 32455143 PMC7222601

[12] De Urioste-Stone SM, Pennington PM, Pellecer E, Aguilar TM, Samayoa G, Perdomo HD, et al. Development of a community-based intervention for the control of Chagas disease based on peridomestic animal management: an eco-bio-social perspective. Trans R Soc Trop Med Hyg. 2015;109(2):159–67. doi: 10.1093/trstmh/tru202 25604767 PMC4299527

[13] Minter-Goedbloed E, Croon JJ. The susceptibility of chickens to Trypanosoma (Schizotrypanum) cruzi. Trans R Soc Trop Med Hyg. 1981;75(3):350–3. doi: 10.1016/0035-9203(81)90090-0 7034309

[14] Flores-Ferrer A, Waleckx E, Rascalou G, Dumonteil E, Gourbière S. Trypanosoma cruzi transmission dynamics in a synanthropic and domesticated host community. PLoS Negl Trop Dis. 2019;13(12):e0007902. doi: 10.1371/journal.pntd.0007902 31834879 PMC6934322

[15] Saavedra M, Zulantay I, Apt W, Castillo J, Araya E, Martínez G, et al. Quantification by real-time PCR of Trypanosoma cruzi DNA in samples of Triatoma infestans used in xenodiagnosis of chronic Chagas disease patients. Parasit Vectors. 2016;9(1):382. doi: 10.1186/s13071-016-1664-5 27377063 PMC4932745

[16] Ramírez JC, Cura CI, da Cruz Moreira O, Lages-Silva E, Juiz N, Velázquez E, et al. Analytical Validation of Quantitative Real-Time PCR Methods for Quantification of Trypanosoma cruzi DNA in Blood Samples from Chagas Disease Patients. J Mol Diagn. 2015;17(5):605–15. doi: 10.1016/j.jmoldx.2015.04.010 26320872 PMC4698797

[17] Rascalou G, Pontier D, Menu F, Gourbière S. Emergence and prevalence of human vector-borne diseases in sink vector populations. PLoS One. 2012;7(5):e36858. doi: 10.1371/journal.pone.0036858 22629337 PMC3356347

[18] Machado EM, Fernandes AJ, Murta SM, Vitor RW, Camilo DJ Jr, Pinheiro SW, et al. A study of experimental reinfection by Trypanosoma cruzi in dogs. Am J Trop Med Hyg. 2001;65(6):958–65. doi: 10.4269/ajtmh.2001.65.958 11792006

[19] Dumonteil E, Gourbière S, Barrera-Pérez M, Rodriguez-Félix E, Ruiz-Pina H, Banos-Lopez O, et al. Geographic distribution of Triatoma dimidiata and transmission dynamics of Trypanosoma cruzi in the Yucatan peninsula of Mexico. The American Journal of Tropical Medicine and Hygiene. 2002;67(2):176–83. 12389944 10.4269/ajtmh.2002.67.176

[20] Péneau J, Nguyen A, Flores-Ferrer A, Blanchet D, Gourbière S. Amazonian Triatomine Biodiversity and the Transmission of Chagas Disease in French Guiana: In Medio Stat Sanitas. PLoS Negl Trop Dis. 2016;10(2):e0004427. doi: 10.1371/journal.pntd.0004427 26867025 PMC4750908

[21] Schofield CJ, Williams NG, Marshall TF. Density-dependent perception of triatomine bug bites. Ann Trop Med Parasitol. 1986;80(3):351–8. doi: 10.1080/00034983.1986.11812028 3541809

[22] Oscherov EB, Damborsky MP, Bar ME, Gorla DE. Competition between vectors of Chagas disease, Triatoma infestans and T. sordida: effects on fecundity and mortality. Med Vet Entomol. 2004;18(4):323–8. doi: 10.1111/j.0269-283X.2004.00526.x 15641997

[23] Pelosse P, Kribs-Zaleta CM, Ginoux M, Rabinovich JE, Gourbière S, Menu F. Influence of vectors’ risk-spreading strategies and environmental stochasticity on the epidemiology and evolution of vector-borne diseases: the example of Chagas’ disease. PLoS One. 2013;8(8):e70830. doi: 10.1371/journal.pone.0070830 23951018 PMC3738595

[24] Reyes-Novelo E, Ruíz-Piña H, Escobedo-Ortegón J, Rodríguez-Vivas I, Bolio-González M, Polanco-Rodríguez Á et al. Current status and perspectives for the study of emergent, re-emergent and neglected zoonotic diseases in the Yucatan peninsula, Mexico. Trop Subtrop Agroecosyst. 2011;14(1):35–54. https://www.revista.ccba.uady.mx/ojs/index.php/TSA/article/view/659?utm

[25] Ellis EA, Hernandez Gomez U, Romero-Montero JA. Los procesos y causas del cambio en la cobertura forestal de la Península Yucatán, México. ECOS. 2017;26(1):101–11. doi: 10.7818/ecos.2017.26-1.16

[26] Rosecrans K, Cruz-Martin G, King A, Dumonteil E. Opportunities for improved chagas disease vector control based on knowledge, attitudes and practices of communities in the yucatan peninsula, Mexico. PLoS Negl Trop Dis. 2014;8(3):e2763. doi: 10.1371/journal.pntd.0002763 24676038 PMC3967964

[27] Sánchez-Soto MF, Gaona O, Vigueras-Galván AL, Suzán G, Falcón LI, Vázquez-Domínguez E. Prevalence and transmission of the most relevant zoonotic and vector-borne pathogens in the Yucatan peninsula: A review. PLoS Negl Trop Dis. 2024;18(7):e0012286. doi: 10.1371/journal.pntd.0012286 38959260 PMC11251636

[28] Gürtler RE, del Pilar Fernández M, Cardinal MV. Eco-Epidemiology of Vector-Borne Transmission of Trypanosoma cruzi in Domestic Habitats. Triatominae - The Biology of Chagas Disease Vectors. 2021;5:447–89. 10.1007/978-3-030-64548-9_17

[29] Payet V, Ramirez-Sierra MJ, Rabinovich J, Menu F, Dumonteil E. Variations in sex ratio, feeding, and fecundity of Triatoma dimidiata (Hemiptera: Reduviidae) among habitats in the Yucatan Peninsula, Mexico. Vector Borne Zoonotic Dis. 2009;9(3):243–51. doi: 10.1089/vbz.2008.0078 19480605

[30] Reyes-Novelo E, Ruiz-Piña H, Escobedo-Ortegón J, Barrera-Pérez M, Manrique-Saide P, Rodríguez-Vivas RI. Triatoma dimidiata (Latreille) abundance and infection with Trypanosoma cruzi in a rural community of Yucatan, Mexico. Neotrop Entomol. 2013;42(3):317–24. doi: 10.1007/s13744-013-0120-x 23949816

[31] Moo-Millan JI, Hernández-Andrade A, May-Concha IJ, Montalvo-Balam T de J, Arnal A, Talavera-Escalante MJ, et al. Temporal variation of Triatoma dimidiata abundance and infection with Trypanosoma cruzi in domestic and sylvatic habitats of rural Yucatan, Mexico. Acta Trop. 2023;248:107038. doi: 10.1016/j.actatropica.2023.107038 37839668

[32] Gourbière S, Dumonteil E, Rabinovich JE, Minkoue R, Menu F. Demographic and dispersal constraints for domestic infestation by non-domicilated chagas disease vectors in the Yucatan Peninsula, Mexico. Am J Trop Med Hyg. 2008;78(1):133–9. doi: 10.4269/ajtmh.2008.78.133 18187796

[33] Barbu C, Dumonteil E, Gourbière S. Characterization of the dispersal of non-domiciliated Triatoma dimidiata through the selection of spatially explicit models. PLoS Negl Trop Dis. 2010;4(8):e777. doi: 10.1371/journal.pntd.0000777 20689823 PMC2914783

[34] Barbu C, Dumonteil E, Gourbière S. Optimization of control strategies for non-domiciliated Triatoma dimidiata, Chagas disease vector in the Yucatán Peninsula, Mexico. PLoS Negl Trop Dis. 2009;3(4):e416. doi: 10.1371/journal.pntd.0000416 19365542 PMC2664331

[35] Barbu C, Dumonteil E, Gourbière S. Evaluation of spatially targeted strategies to control non-domiciliated Triatoma dimidiata vector of Chagas disease. PLoS Negl Trop Dis. 2011;5(5):e1045. doi: 10.1371/journal.pntd.0001045 21610862 PMC3096612

[36] Castillo-Neyra R, Borrini Mayorí K, Salazar Sánchez R, Ancca Suarez J, Xie S, Náquira Velarde C, et al. Heterogeneous infectiousness in guinea pigs experimentally infected with Trypanosoma cruzi. Parasitol Int. 2016;65(1):50–4. doi: 10.1016/j.parint.2015.09.009 26432777 PMC4657135

[37] Hernandez-Andrade A. Descripción de los ciclos de transmisión de Trypanosoma cruzi mediante la identificación de las interacciones hospedero/vector/parásito/ecotopos en localidades rurales de Yucatán. Universidad Autonoma de Yucatán. 2020.

[38] Moo-Millan J. Estudio longitudinal de las interacciones hospederos/ vector/parásito/microbioma/habitat que definen los ciclos de transmision de Trypanosoma cruzi en Yucatán. Universidad Autonoma de Yucatán. 2024.

[39] Nouvellet P, Cucunubá ZM, Gourbière S. Ecology, evolution and control of Chagas disease: a century of neglected modelling and a promising future. Adv Parasitol. 2015;87:135–91. doi: 10.1016/bs.apar.2014.12.004 25765195

[40] Instituto Nacional de Estadıstica y Geografıa. Encuesta Intercensal 2015. 2015. https://www.inegi.org.mx

[41] Dumonteil E, Nouvellet P, Rosecrans K, Ramirez-Sierra MJ, Gamboa-León R, Cruz-Chan V, et al. Eco-bio-social determinants for house infestation by non-domiciliated Triatoma dimidiata in the Yucatan Peninsula, Mexico. PLoS Negl Trop Dis. 2013;7(9):e2466. doi: 10.1371/journal.pntd.0002466 24086790 PMC3784500

[42] Panti-May JA, Hernández-Betancourt S, Ruíz-Piña H, Medina-Peralta S. Abundance and population parameters of commensal rodents present in rural households in Yucatan, Mexico. International Biodeterioration & Biodegradation. 2012;66(1):77–81. doi: 10.1016/j.ibiod.2011.10.006

[43] Bosseno M-F, García LS, Baunaure F, Gastelúm EM, Gutierrez MS, Kasten FL, et al. Identification in triatomine vectors of feeding sources and Trypanosoma cruzi variants by heteroduplex assay and a multiplex miniexon polymerase chain reaction. Am J Trop Med Hyg. 2006;74(2):303–5. doi: 10.4269/ajtmh.2006.74.303 16474087

[44] Catalá S. The biting rate of Triatoma infestans in Argentina. Med Vet Entomol. 1991;5(3):325–33. doi: 10.1111/j.1365-2915.1991.tb00558.x 1768924

[45] Zeledón R, Guardia VM, Zúñiga A, Swartzwelder JC. Biology and ethology of Triatoma dimidiata (Latreille, 1811): I. Life cycle, amount of blood ingested, resistance to starvation, and size of adults. Journal of Medical Entomology. 1970;7(3):313–9. 4915977 10.1093/jmedent/7.3.313

[46] Nogueda-Torres B, Montañez-Valdez OD, Michel-Parra JG, Martínez-Grant DM, Martínez-Ibarra JA. Biological Parameters of Three Populations of Triatoma dimidiata s. s. (Hemiptera: Reduviidae) From Western Mexico. J Med Entomol. 2021;58(6):2114–23. doi: 10.1093/jme/tjab116 34224558

[47] Dumonteil E, Ruiz-Piña H, Rodriguez-Félix E, Barrera-Pérez M, Ramirez-Sierra MJ, Rabinovich JE, et al. Re-infestation of houses by Triatoma dimidiata after intra-domicile insecticide application in the Yucatán peninsula, Mexico. Mem Inst Oswaldo Cruz. 2004;99(3):253–6. doi: 10.1590/s0074-02762004000300002 15273795

[48] Montes de Oca-Aguilar AC, González-Martínez A, Chan-González R, Ibarra-López P, Smith-Ávila S, Córdoba-Aguilar A, et al. Signs of Urban Evolution? Morpho-Functional Traits Co-variation Along a Nature-Urban Gradient in a Chagas Disease Vector. Front Ecol Evol. 2022;10. doi: 10.3389/fevo.2022.805040

[49] Zeledon R. El triatoma dimidiata (Latreille, 1811) y su relacion con la enfermedad de Chagas. San Jose, Costa Rica: UNED. 1981.

[50] Marín-Ortiz JC, Parra-Henao G, Altamiranda-Saavedra M, Jaramillo-O N. Characterization of Feeding Behavior and its Relationship With the Longevity of Wild and Peridomestic Triatoma dimidiata, Latreille 1811 (Hemiptera, Reduviidae) Under Laboratory Conditions. Journal of Medical Entomology. 2022;59(6):1911–20. 35980342 10.1093/jme/tjac122

[51] Gamboa-León R, Ramirez-Gonzalez C, Pacheco-Tucuch FS, O’Shea M, Rosecrans K, Pippitt J, et al. Seroprevalence of Trypanosoma cruzi among mothers and children in rural Mayan communities and associated reproductive outcomes. Am J Trop Med Hyg. 2014;91(2):348–53. doi: 10.4269/ajtmh.13-0527 24935948 PMC4125261

[52] Jimenez-Coello M, Poot-Cob M, Ortega-Pacheco A, Guzman-Marin E, Ramos-Ligonio A, Sauri-Arceo CH, et al. American trypanosomiasis in dogs from an urban and rural area of Yucatan, Mexico. Vector Borne Zoonotic Dis. 2008;8(6):755–61. doi: 10.1089/vbz.2007.0224 18597661

[53] Jiménez‐Coello M, Acosta‐Viana KY, Guzman‐Marin E, Gomez‐Rios A, Ortega‐Pacheco A. Epidemiological survey of Trypanosoma cruzi infection in domestic owned cats from the tropical southeast of Mexico. Zoonoses and Public Health. 2012;59:102–9. 22958254 10.1111/j.1863-2378.2012.01463.x

[54] Izeta-Alberdi A, Pech-May A, Tun-Ku E, Mazariegos-Hidalgo CJ, López-Cancino SA, Gutiérrez S, et al. Trypanosoma cruzi in Mexican Neotropical vectors and mammals: wildlife, livestock, pets, and human population. Salud Publica Mex. 2023;65(2 mar-abr):114–26. doi: 10.21149/13801 38060864

[55] Dumonteil E, Tripet F, Ramirez-Sierra MJ, Payet V, Lanzaro G, Menu F. Assessment of Triatoma dimidiata dispersal in the Yucatan Peninsula of Mexico by morphometry and microsatellite markers. Am J Trop Med Hyg. 2007;76(5):930–7. doi: 10.4269/ajtmh.2007.76.930 17488918

[56] Ramirez-Sierra MJ, Herrera-Aguilar M, Gourbière S, Dumonteil E. Patterns of house infestation dynamics by non-domiciliated Triatoma dimidiata reveal a spatial gradient of infestation in rural villages and potential insect manipulation by Trypanosoma cruzi. Trop Med Int Health. 2010;15(1):77–86. doi: 10.1111/j.1365-3156.2009.02422.x 19912593

[57] Giojalas LC, Catalá SS, Asin SN, Gorla DE. Seasonal changes in infectivity of domestic populations of Triatoma infestans. Trans R Soc Trop Med Hyg. 1990;84(3):439–42. doi: 10.1016/0035-9203(90)90355-i 2124395

[58] Ibáñez-Cervantes G, León-García G, Castro-Escarpulli G, Mancilla-Ramírez J, Victoria-Acosta G, Cureño-Díaz MA, et al. Evolution of incidence and geographical distribution of Chagas disease in Mexico during a decade (2007-2016). Epidemiol Infect. 2018;147:e41. doi: 10.1017/S0950268818002984 30421698 PMC6518600

[59] Sartor P, Colaianni I, Cardinal MV, Bua J, Freilij H, Gürtler RE. Improving access to Chagas disease diagnosis and etiologic treatment in remote rural communities of the Argentine Chaco through strengthened primary health care and broad social participation. PLoS Negl Trop Dis. 2017;11(2):e0005336. doi: 10.1371/journal.pntd.0005336 28192425 PMC5325580

[60] Cohen JE, Gürtler RE. Modeling household transmission of American trypanosomiasis. Science. 2001;293(5530):694–8. doi: 10.1126/science.1060638 11474111

[61] Gurtler RE, Cohen JE, Cecere MC, Lauricella MA, Chuit R, Segura EL. Influence of humans and domestic animals on the household prevalence of Trypanosoma cruzi in Triatoma infestans populations in northwest Argentina. Am J Trop Med Hyg. 1998;58(6):748–58. doi: 10.4269/ajtmh.1998.58.748 9660458

[62] Gurevitz JM, Ceballos LA, Gaspe MS, Alvarado-Otegui JA, Enríquez GF, Kitron U, et al. Factors affecting infestation by Triatoma infestans in a rural area of the humid Chaco in Argentina: a multi-model inference approach. PLoS Negl Trop Dis. 2011;5(10):e1349. doi: 10.1371/journal.pntd.0001349 22028941 PMC3196485

[63] Bustamante DM, De Urioste-Stone SM, Juárez JG, Pennington PM. Ecological, social and biological risk factors for continued Trypanosoma cruzi transmission by Triatoma dimidiata in Guatemala. PLoS One. 2014;9(8):e104599. doi: 10.1371/journal.pone.0104599 25170955 PMC4149347

[64] Rosal GG, Nogueda-Torres B, Villagrán ME, de Diego-Cabrera JA, Montañez-Valdez OD, Martínez-Ibarra JA. Chagas disease: Importance of rats as reservoir hosts of Trypanosoma cruzi (Chagas, 1909) in western Mexico. J Infect Public Health. 2018;11(2):230–3. doi: 10.1016/j.jiph.2017.07.017 28774654

[65] Valença-Barbosa C, Lima MM, Sarquis O, Bezerra CM, Abad-Franch F. Modeling disease vector occurrence when detection is imperfect II: Drivers of site-occupancy by synanthropic Triatoma brasiliensis in the Brazilian northeast. PLoS Negl Trop Dis. 2014;8(5):e2861. doi: 10.1371/journal.pntd.0002861 24811125 PMC4014420

[66] Fiatsonu E, Deka A, Ndeffo-Mbah ML. Effectiveness of Systemic Insecticide Dog Treatment for the Control of Chagas Disease in the Tropics. Biology (Basel). 2023;12(9):1235. doi: 10.3390/biology12091235 37759635 PMC10525078

[67] da Oliveira MT, Branquinho RT, Alessio GD, Mello CGC, Nogueira-de-Paiva NC, Carneiro CM, et al. TcI, TcII and TcVI Trypanosoma cruzi samples from Chagas disease patients with distinct clinical forms and critical analysis of in vitro and in vivo behavior, response to treatment and infection evolution in murine model. Acta Trop. 2017;167:108–20. doi: 10.1016/j.actatropica.2016.11.033 27908747

