# Supplementary material for: Seasonal variations of Triatoma dimidiata demography and Trypanosoma cruzi transmission within its multi-host community in the Yucatan Peninsula, Mexico: Insights from an integrative SIR eco-epidemiological modelling
Source: PLoS Negl Trop Dis. 2026 Jul 15;20(7):e0014500. doi: 10.1371/journal.pntd.0014500 (PMC13384399; doi:10.1371/journal.pntd.0014500)
Supplement: S1 Text — Fig A in S1 Text: Monthly variations of T. dimidiata female proportion in villages. The proportions of female T. dimidiata vectors observed from insect captures conducted in 2017 (circle), 2018 (triangle) and 2019 (square), and their average (red) were estimated for each month. Table A in S1 Text: Nymph and adult vectors feeding preferences. The percentage of bloodmeals of T. dimidiata adults or nymphs, infected or not by T. cruzi and collected in houses or backyards are attributed to the 5 main hosts observed through metabarcoding analyses. Fig B in S1 Text: Seasonal variations of T. cruzi vector prevalence and host prevalences (A), incidences and acute-chronic cases (B) for a rodent life-expectancy of 24 months. The seasonal variations in the prevalence of infection by T. cruzi, in the incidence of infection (number of newly infected individuals), and the number of infectious and chronic cases are represented for humans (red), dogs (blue), cats (brown) and rodents (orange) and for T. dimidiata adults (black) for a 35 years-period. Links to the sources of the black icons used to represent hosts species are available in S1 File. Fig C in S1 Text: Seasonal variations of T. cruzi vector prevalence and host prevalences (A), incidences and acute-chronic cases (B) for a proportion of 7% of T. dimidiata adult bloodmeals made on rodents. The seasonal variations in the prevalence of infection by T. cruzi, in the incidence of infection (number of newly infected individuals), and the number of infectious and chronic cases are represented for humans (red), dogs (blue), cats (brown) and rodents (orange) and for T. dimidiata adults (black) for a 35 years-period. Links to the sources of the black icons used to represent hosts species are available in S1 File. Fig D in S1 Text: Seasonal variations of T. cruzi vector prevalence and host prevalences (A), incidences and acute-chronic cases (B) for a duration of the acute (and infectious) phase of 8 months. The seasonal variations in [file pntd.0014500.s001.docx]

**Supplementary Materials**

**S1 Text.** Mathematical modelling of *T. cruzi* transmission within its host community.

The mathematical modelling of the vector borne transmission of *T. cruzi* within its host community that was represented in Figure 1 relies on the formal representations of i) the impact of *T. dimidiata* intraspecific competition on the vector’s stage specific blood-feeding rates ($\beta_{i}$ and $\beta_{A}$), ii) the rates of *T. cruzi* transmission from hosts to vectors ($\mathcal{S}_{N}$ and $\mathcal{S}_{A}$) and from vectors to hosts ($\mathcal{T}_{N}$ and $\mathcal{T}_{A}$) iii) the probability of vector survival and development into the next life-history stage ($s_{i}$).

- The vector intraspecific competition for bloodmeals was described as in Flores-Ferrer et al. [14], using the well-established Maynard-Smith’s [68] function to describe the competitive interactions between nymphs (Equation 1) and between adults (Equation 2) vectors as :

$\beta_{i}\left( \boldsymbol{N},\boldsymbol{H} \right)\boldsymbol{=}\frac{\beta_{imax}}{1 + {}_{N} \frac{\boldsymbol{N}}{N_{HN}}}$ (1)

$\beta_{A}\left( \boldsymbol{A},\boldsymbol{H} \right)\boldsymbol{=}\frac{\beta_{Amax}}{1 + {}_{A} \frac{\boldsymbol{A}}{N_{HA}}}$ (2)

This allows the stage specific maximum per vector blood-feeding rate of nymphs ($\beta_{imax}$) and adults ($\beta_{Amax}$*)* to be downregulated as the ratio between the densities of nymphs (***N***) and adults (***A***) of *T. dimidiata* and hosts (***H***) increases, and according to the intensity of the density-dependent regulation of nymphs (*γ_N_*) and adults (*γ_A_*). The ‘effective’ size of the host communities $N_{HN}$ and $N_{HA}$ were then defined as the sum of all host species abundance weighted by nymphs and adults blood-feeding rates *αNh* and *αAh*, which led to the quantities$N_{HN}=\Sigma_{h}.\alpha_{Nh}.N_{h}$ and $N_{HA}=\Sigma_{h}.\alpha_{Ah}.N_{h}$.

- The rates of *T. cruzi* transmission from infectious hosts to susceptible nymph $\mathcal{(S}_{N})$ and adult $\mathcal{(S}_{A})$ vectors were also described as in Flores-Ferrer et al. [14] :

$\mathcal{S}_{N}{=\Sigma}_{h}. {}_{Nh}. \frac{I^{h}}{N^{h}} . p_{v}$ (3)

$\mathcal{S}_{A}{=\Sigma}_{h}. {}_{Ah}. \frac{I^{h}}{N^{h}} . p_{v}$ (4)

where ${}_{Nh}$ and ${}_{Ah}$ denote the proportions of nymphs and adults bloodmeals made on host h and $p_{v}$stands for the probability of vector infection from infected hosts. The ratio $\frac{I^{h}}{N^{h}}$ in those expression ensure that only bites made on infected hosts are potentially infectious.

Similarly, the transmission of *T. cruzi* from infectious nymph ${(\mathcal{T}}_{N})$ and adult ${(\mathcal{T}}_{A})$ vectors to susceptible hosts were modelled as it follows :

$\mathcal{T}_{N}={}_{Nh}. \frac{S^{h}}{N^{h}} . p_{h}$ (5)

$\mathcal{T}_{A}={}_{Ah}. \frac{S^{h}}{N^{h}} . p_{h}$ (6)

where $p_{h}$ stands for the probability of host h infection from infected vectors, and where the ratio $\frac{S^{h}}{N^{h}}$ ensure that only bites made on susceptible hosts potentially lead to a new host infection.

In order to account for the duration of each *T. dimidiata* developmental stages, we set the time step of the model to match the duration of the shortest stage, which was found to be the egg stage that typically lasts for a month ([48], p. 65). According to this standard modelling routine described in Caswell ([69], p160-161), all nymphal stages (i=1 to 5) where then subdivided into 1-month long substages to fit to their own average duration. Nymph 1, 2, 3, 4, 5 where then modelled as lasting for 1, 2, 2, 2 and 3 sub-stages, respectively. In order to ease the writing of the model, we used a common index j numbering all substages from 1 to 10, so that j takes on values 1 (for nymphs 1), 2-3 (for nymphs 2), 4-5 (for nymphs 3), 6-7 (for nymphs 4) and 8-9-10 (for nymphs 5), and defined the function of vector survival and development accordingly.

- The vector immatures were considered to develop through all the nymphal substages according to basic biological mechanisms that we intended to represent as simply as possible. To develop into the next substage, individuals must survive and eventually molt into the next life-history stage. For a nymph to molt typically requires at least one blood meal to be taken, and we therefore calculated the average amount of bloodmeals made by an individual about to molt during its current nymphal stage, which we denoted $\boldsymbol{P}_{\boldsymbol{j}}^{\boldsymbol{N}}$. This quantity was merely obtained by summing the bloodmeal frequencies defined above (by equation 1) over the current stage duration. The probability of molting into a new nymphal stage was then considered to be max($\boldsymbol{P}_{\boldsymbol{j}}^{\boldsymbol{N}}$,1) and the probability to go from substage j to the next was set to $s_{j}$ when the demographic transition did not involve molting (i.e. for j = 2, 4, 6, 8 and 9), and to

$s_{j}\left( \mathbf{N},\mathbf{H} \right)\mathbf{=}s_{j}\boldsymbol{*P}_{\boldsymbol{j}}^{\boldsymbol{N}}$

when it did (i.e. for j = 1, 3, 5, 7 and 10).

In both cases, the maximal surviving rate of the substage j was set to a common stage specific value given for i=1 to 5 in table 1.

The above specifications allowed to write down the set of dynamical equations describing the monthly variations in the number of susceptible and infectious *T. dimidiata* eggs, nymphs and adults (Equations 8-12), as well as the concomitant variations in the number of susceptible, infectious and recovered hosts (Equation 13-16).

First, susceptible eggs are produced by adult females according to the simple relationship :

$S_{e}\left( t+1 \right)={\boldsymbol{A}\left( t \right)\boldsymbol{.}p_{f}\left( t \right)}. \beta_{A}\left( \boldsymbol{A}\left( t \right),\boldsymbol{H} \right) {.b}_{A}$ (7)

where $\boldsymbol{A}(t)$ corresponds to the number of adult vectors at time t, $p_{f}(t)$ the proportion of females among adults at time t, which feed at rate $\beta_{A}\left( \boldsymbol{A}\left( t \right),\boldsymbol{H} \right)$ and produce $b_{A}$ eggs per bloodmeal.

Second, eggs that survive at rate s_e_ develop into first instar nymphs (j=1);

$S_{N_{j=1}}\left( t+1 \right)=S_{e}\left( t \right) .s_{e}$ (8)

Third, susceptible and infectious individuals in each sub-stage (j=2 to 10) are given by :

$S_{N_{j+1}}\left( t+1 \right)=(S_{N_{j}}\left( t \right) -\beta_{i}\left( \boldsymbol{N},\boldsymbol{H} \right) . \mathcal{S}_{N}. S_{N_{j}}\left( t \right)) . s_{i}(\boldsymbol{N},\boldsymbol{H)}$ (9)

$I_{N_{j+1}}\left( t+1 \right)=I_{N_{j}}\left( t \right) . s_{i}\left( \boldsymbol{N},\boldsymbol{H} \right)+\beta_{i}\left( \boldsymbol{N},\boldsymbol{H} \right) . \mathcal{S}_{N} .S_{N_{j}}\left( t \right). s_{i}$ (10)

Fourth, equations for susceptible and infectious adults were similarly derived as :

$S_{A}\left( t+1 \right)=M_{S}+\left( S_{A}\left( t \right)-\beta_{A}\left( \boldsymbol{A},\boldsymbol{H} \right).\mathcal{S}_{A}. S_{A}\left( t \right) \right). s_{A}+(S_{N_{10}}\left( t \right)\boldsymbol{-}\beta_{5}\left( \boldsymbol{N},\boldsymbol{H} \right).\mathcal{S}_{N}. S_{N_{10}}\left( t \right)){.s}_{5}(\boldsymbol{N},\boldsymbol{H)}$ (11)

$I_{A}\left( t+1 \right)=M_{I}+(I_{A}(t)+\beta_{A}\left( \boldsymbol{A},\boldsymbol{H} \right).\mathcal{S}_{A}. S_{A}\left( t \right)). s_{A}+I_{N_{10}}\left( t \right). s_{5}(\boldsymbol{N},\boldsymbol{H)}+\beta_{5}\left( \boldsymbol{N},\boldsymbol{H} \right).\mathcal{S}_{N}. S_{N_{10}}\left( t \right)). s_{5}$ (12)

Finaly, the following set of equations was defined to predict the number of susceptible (Equation 13), newly infected individuals (i.e. entering into the acute phasis of infection, Equation 14), infectious individuals (i.e. in the acute phasis of infection, Equation 15) or recovered individuals (i.e. in the chronic phasis of infection, Equation 16) individuals of host species *H.*

$S_{h}\left( t+1 \right)=B_{h}+(S_{h}\left( t \right)-\beta_{A}\left( \boldsymbol{A},\boldsymbol{H} \right).\mathcal{T}_{A}.I_{A}\left( t \right)- \beta_{i}\left( \boldsymbol{N},\boldsymbol{H} \right).\mathcal{T}_{N}.I_{N_{j}}\left( t \right)). s_{h}$ (13)

$I_{h_{x=1}}\left( t+1 \right)={(\beta}_{A}\left( \boldsymbol{A},\boldsymbol{H} \right).\mathcal{T}_{A}.I_{A}\left( t \right)+\beta_{i}\left( \boldsymbol{N},\boldsymbol{H} \right).\mathcal{T}_{N}.I_{N_{j}}\left( t \right)). s_{h}$ (14)

$I_{h_{x+1}}(t+1)=I_{h_{x}}(t). s_{h}$ (15)

$R_{h}(t+1)=R_{h}(t). s_{h}+ I_{h_{x=5}}(t). s_{h}$ (16)

where the infectious individuals where subdivided into x 1-month long substages in order to control the duration of the acute period. In the reference situation the duration of the acute phasis was set to 5 months, so that 5 substages were defined.

**Fig A : Monthly variations of *T. dimidiata* female proportion in villages.** The proportions of female *T. dimidiata* vectors observed from insect captures conducted in 2017 (circle), 2018 (triangle) and 2019 (square), and their average (red) were estimated for each month.

**
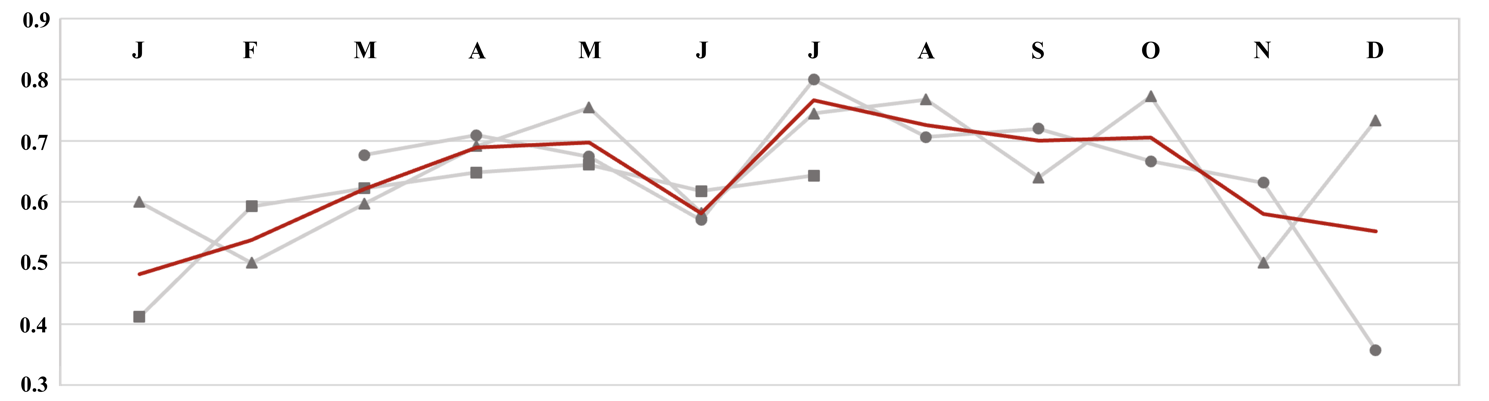
**

**Table A : Nymph and adult vectors feeding preferences.** The percentage of bloodmeals of *T. dimidiata* adults or nymphs, infected or not by *T. cruzi* and collected in houses or backyards are attributed to the 5 main hosts observed through metabarcoding analyses.

|  | Houses | | | | Backyards | | | | Total | |
| --- | --- | --- | --- | --- | --- | --- | --- | --- | --- | --- |
|  | *T. dimidiata* adults | | *T. dimidiata* nymphs | | *T. dimidiata* adults | | *T. dimidiata* nymphs | | *T. dimidiata* | |
|  | *T. cruzi* infected | Non-infected | *T. cruzi* infected | Non-infected | *T. cruzi* infected | Non-infected | *T. cruzi* infected | Non-infected | Adults | Nymphs |
| Human | 53.1% | 55.6% | 0% | 100% | X | 33.3% | X | 38.5% | 53.7% | 37.2% |
| Dog | 18.4% | 18.1% | 100% | 0% | X | 14.3% | X | 10.3% | 18% | 16.3% |
| Cat | 5.1% | 2.3% | 0% | 0% | X | 0% | X | 2.6% | 2.9% | 2.3% |
| Rodent | 21.4% | 22.4% | 0% | 0% | X | 9.5% | X | 15.4% | 21.4% | 14% |
| Avian | 2% | 1.5% | 0% | 0% | X | 42.9% | X | 33.3% | 4% | 30.2% |
|  | n = 98 | n = 259 | n = 3 | n = 1 | n = 0 | n = 21 | n = 0 | n = 39 | n = 378 | n = 43 |

**
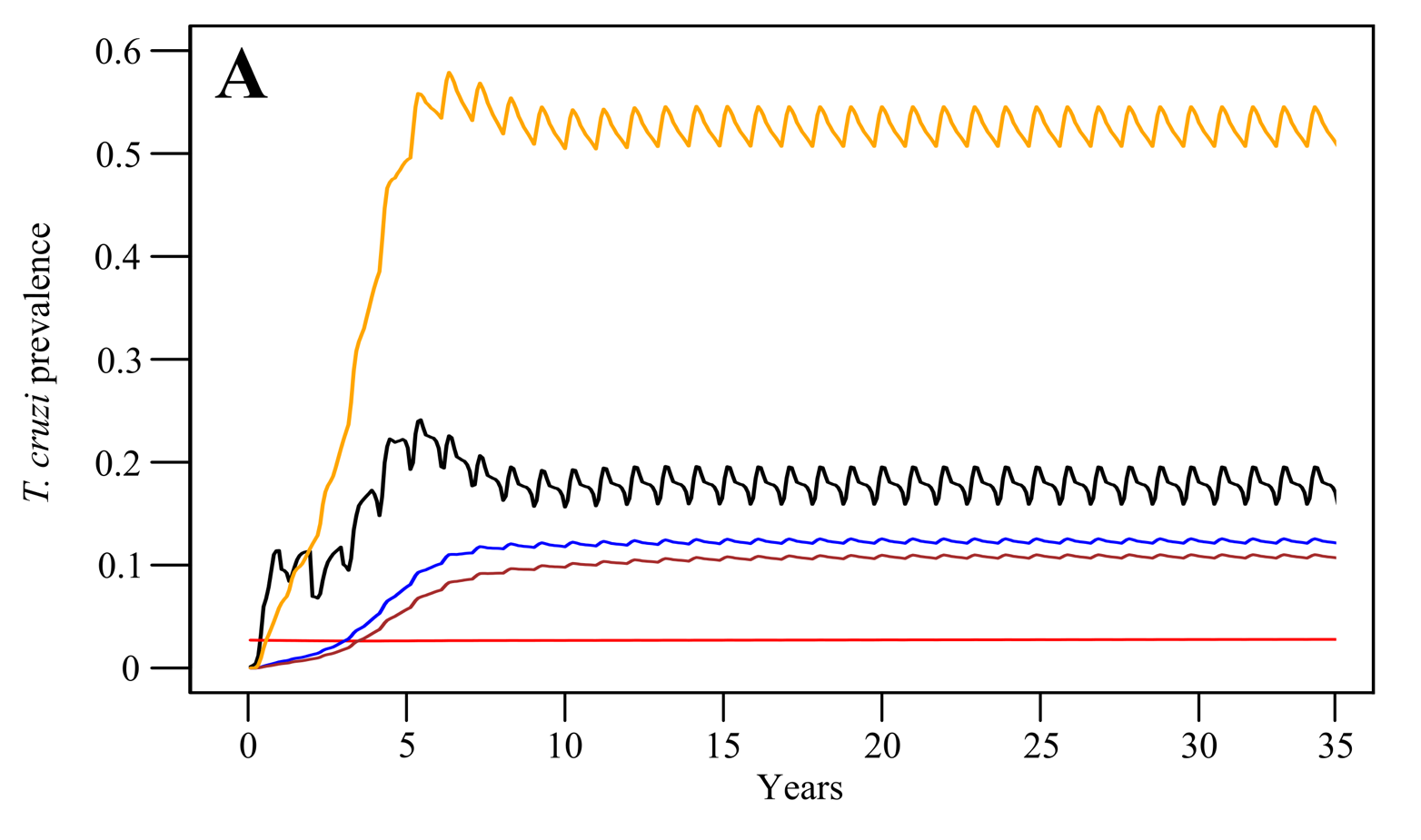
Fig B : Seasonal variations of *T. cruzi* vector prevalence and host prevalences (A), incidences and acute-chronic cases (B) for a rodent life-expectancy of 24 months.** The seasonal variations in the prevalence of infection by *T. cruzi*, in the incidence of infection (number of newly infected individuals), and the number of infectious and chronic cases are represented for humans (red), dogs (blue), cats (brown) and rodents (orange) and for *T. dimidiata* adults (black) for a 35 years-period. Links to the sources of the black icons used to represent hosts species are available in S1 File.

**
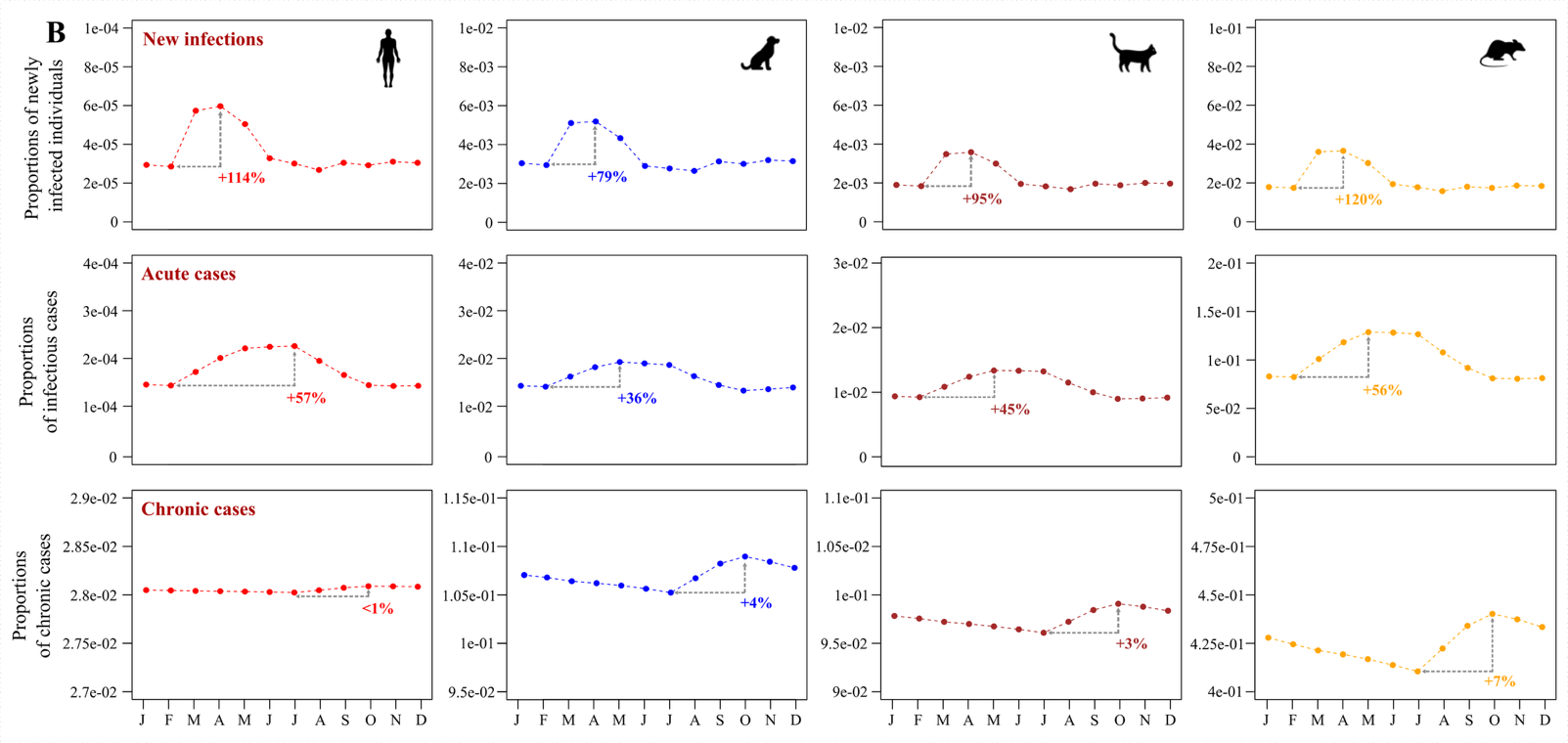
**

**
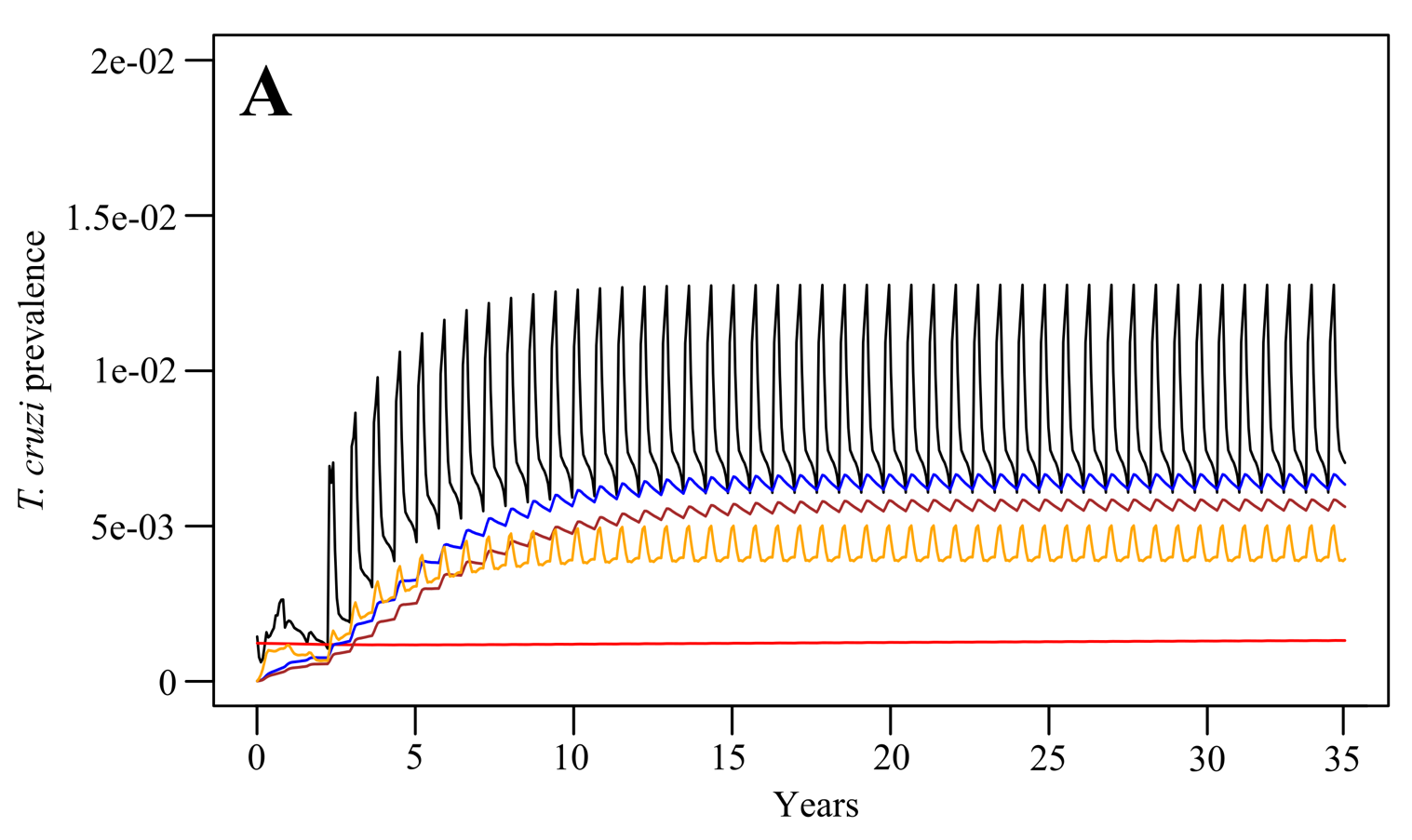
Fig C : Seasonal variations of *T. cruzi* vector prevalence and host prevalences (A), incidences and acute-chronic cases (B) for a proportion of 7% of *T. dimidiata* adult bloodmeals made on rodents.** The seasonal variations in the prevalence of infection by *T. cruzi*, in the incidence of infection (number of newly infected individuals), and the number of infectious and chronic cases are represented for humans (red), dogs (blue), cats (brown) and rodents (orange) and for *T. dimidiata* adults (black) for a 35 years-period. Links to the sources of the black icons used to represent hosts species are available in S1 File.

**
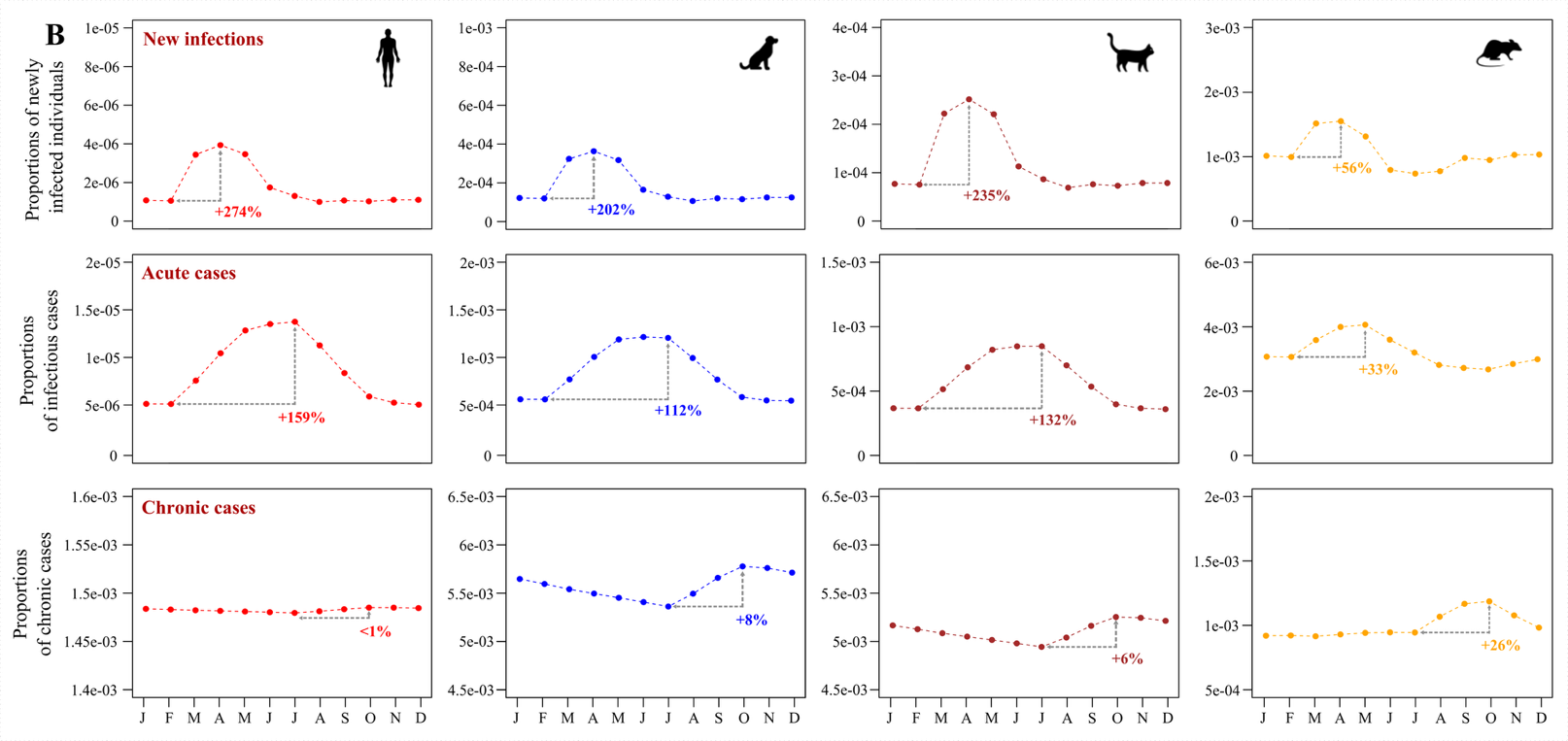
**

**
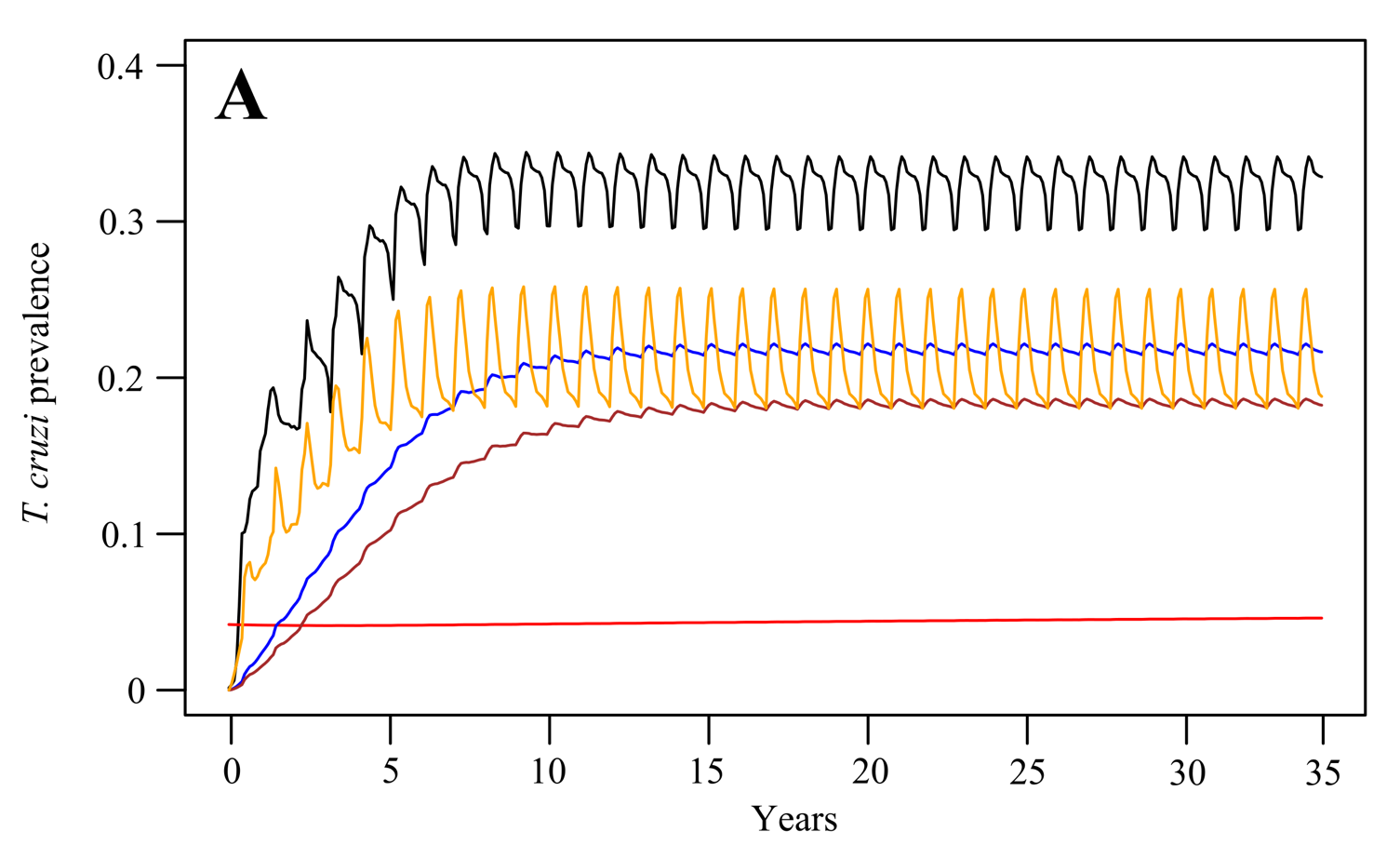

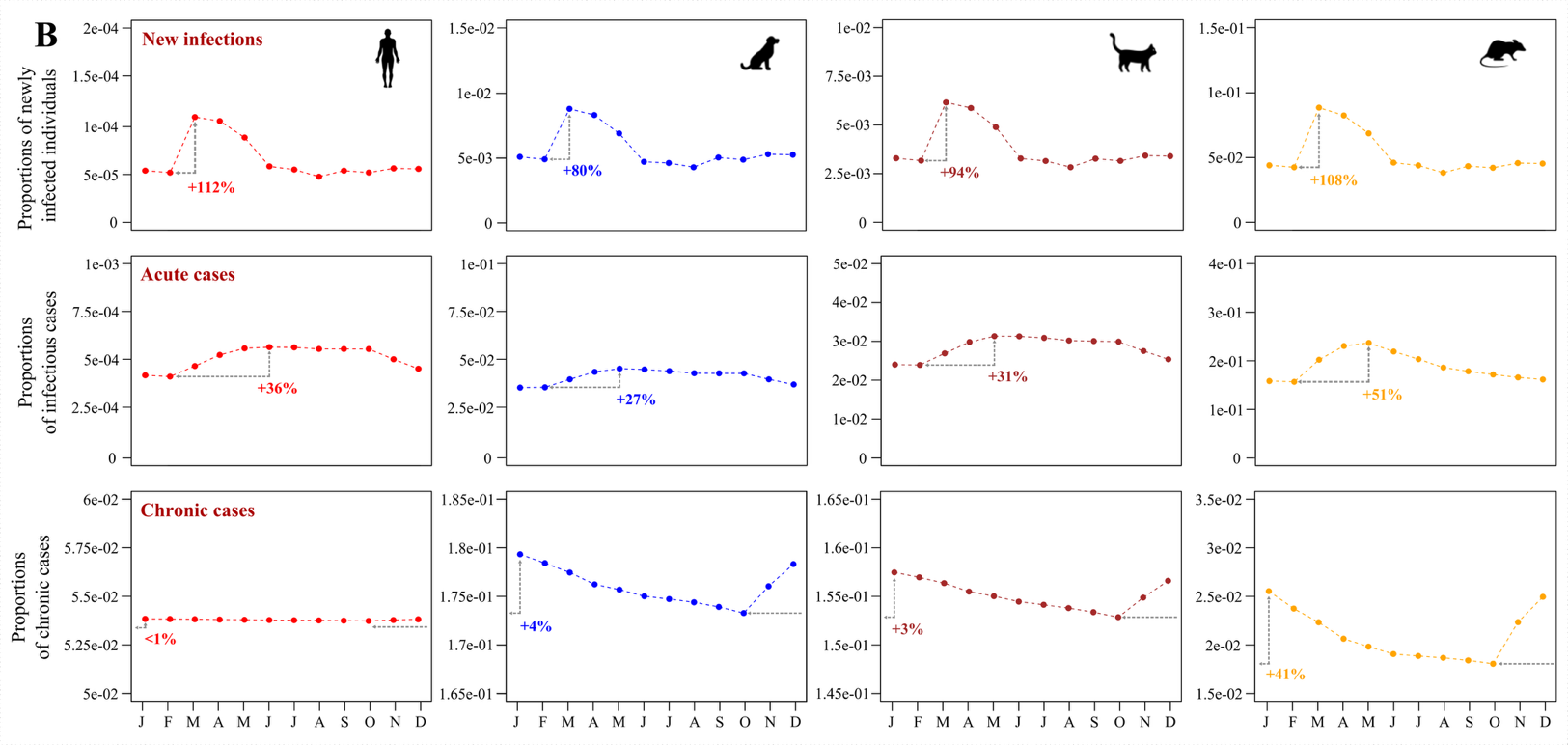
Fig D : Seasonal variations of *T. cruzi* vector prevalence and host prevalences (A), incidences and acute-chronic cases (B) for a duration of the acute (and infectious) phase of 8 months.** The seasonal variations in the prevalence of infection by *T. cruzi*, in the incidence of infection (number of newly infected individuals), and the number of infectious and chronic cases are represented for humans (red), dogs (blue), cats (brown) and rodents (orange) and for *T. dimidiata* adults (black) for a 35 years-period. Links to the sources of the black icons used to represent hosts species are available in S1 File.

**Fig E : Seasonal variations of *T. cruzi* vector prevalence and host prevalences (A), incidences and acute-chronic cases (B) for a duration of the acute (and infectious) phase of 12 months.** The seasonal variations in the prevalence of infection by *T. cruzi*, in the incidence of infection (number of newly infected individuals), and the number of infectious and chronic cases are represented for humans (red), dogs (blue), cats (brown) and rodents (orange) and for *T. dimidiata* adults (black) for a 35 years-period. Links to the sources of the black icons used to represent hosts species are available in S1 File.

**
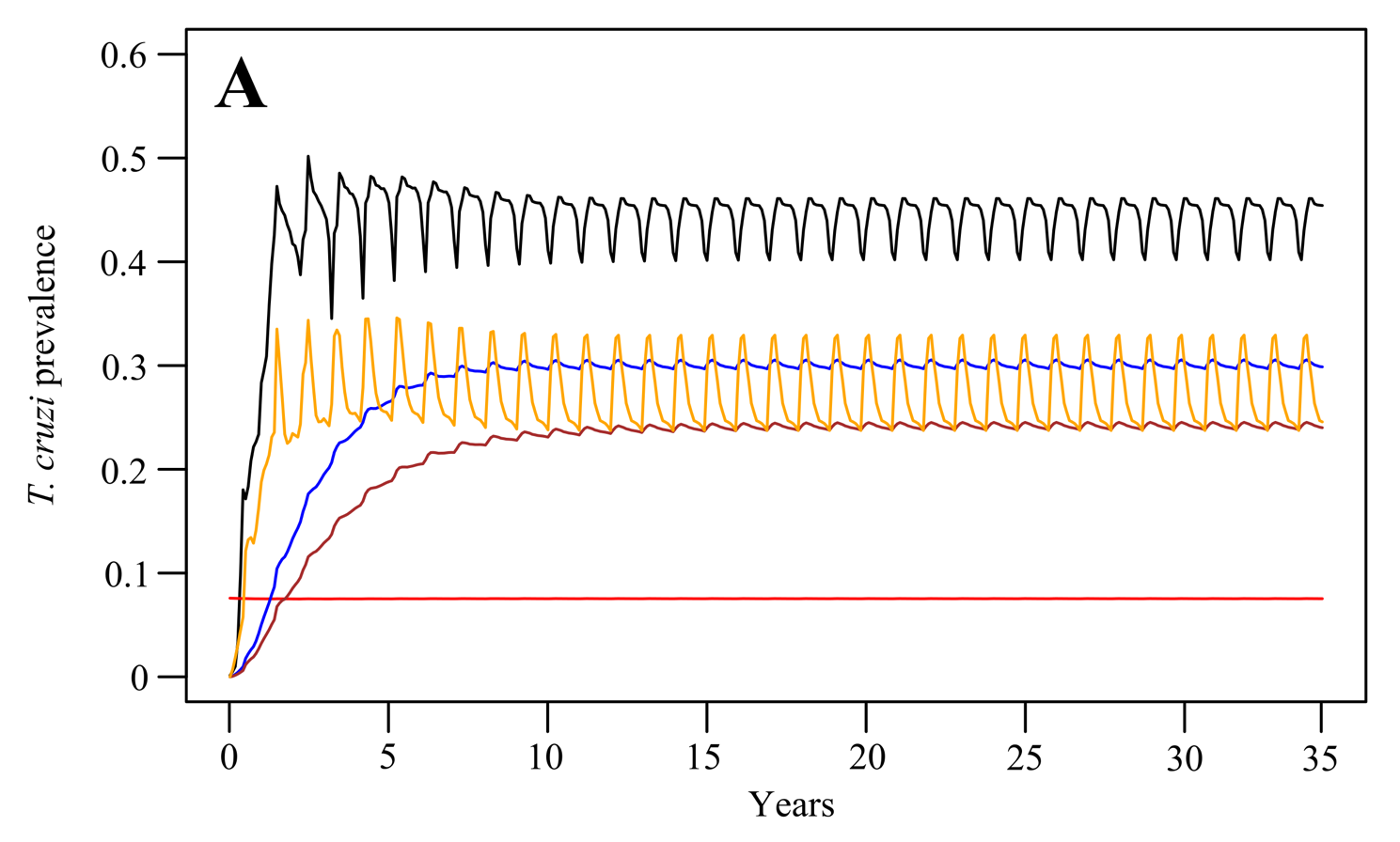
**

**
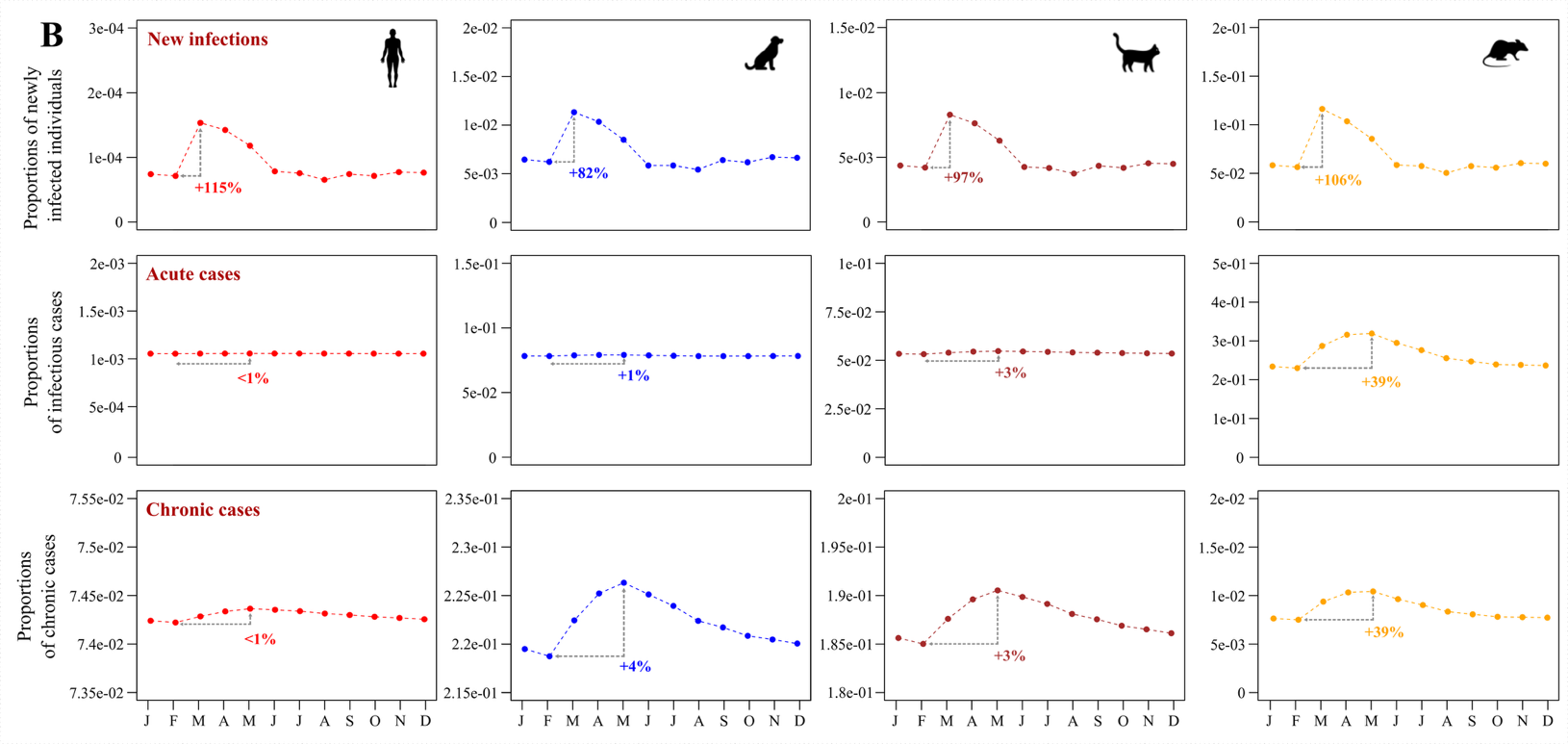
**

**Fig F : Seasonal variations of *T. cruzi* vector prevalence and host prevalences (A), incidences and acute-chronic cases (B) for a duration of the acute (and infectious) phase of 18 months.** The seasonal variations in the prevalence of infection by *T. cruzi*, in the incidence of infection (number of newly infected individuals), and the number of infectious and chronic cases are represented for humans (red), dogs (blue), cats (brown) and rodents (orange) and for *T. dimidiata* adults (black) for a 35 years-period. Links to the sources of the black icons used to represent hosts species are available in S1 File.

**
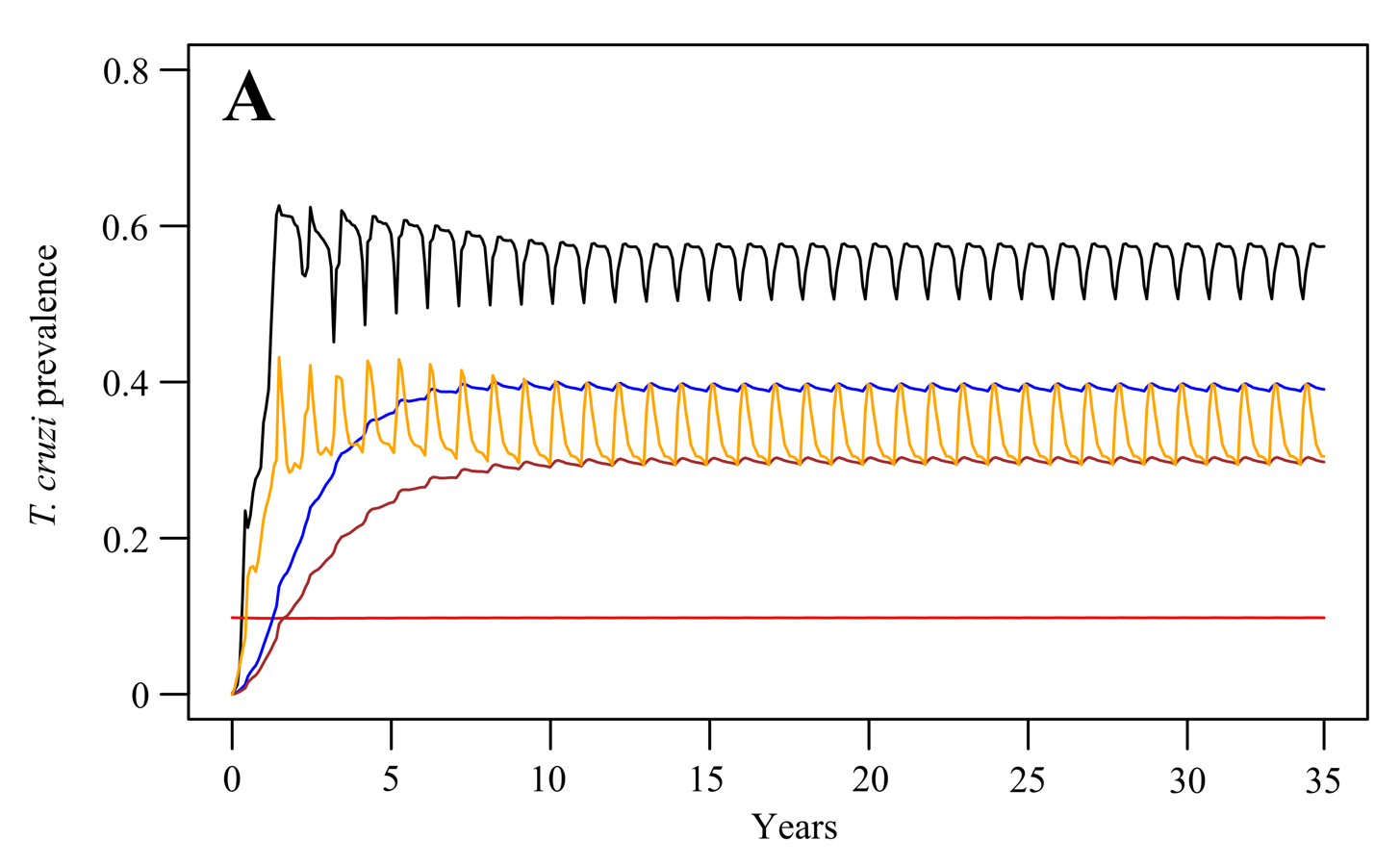
**

**
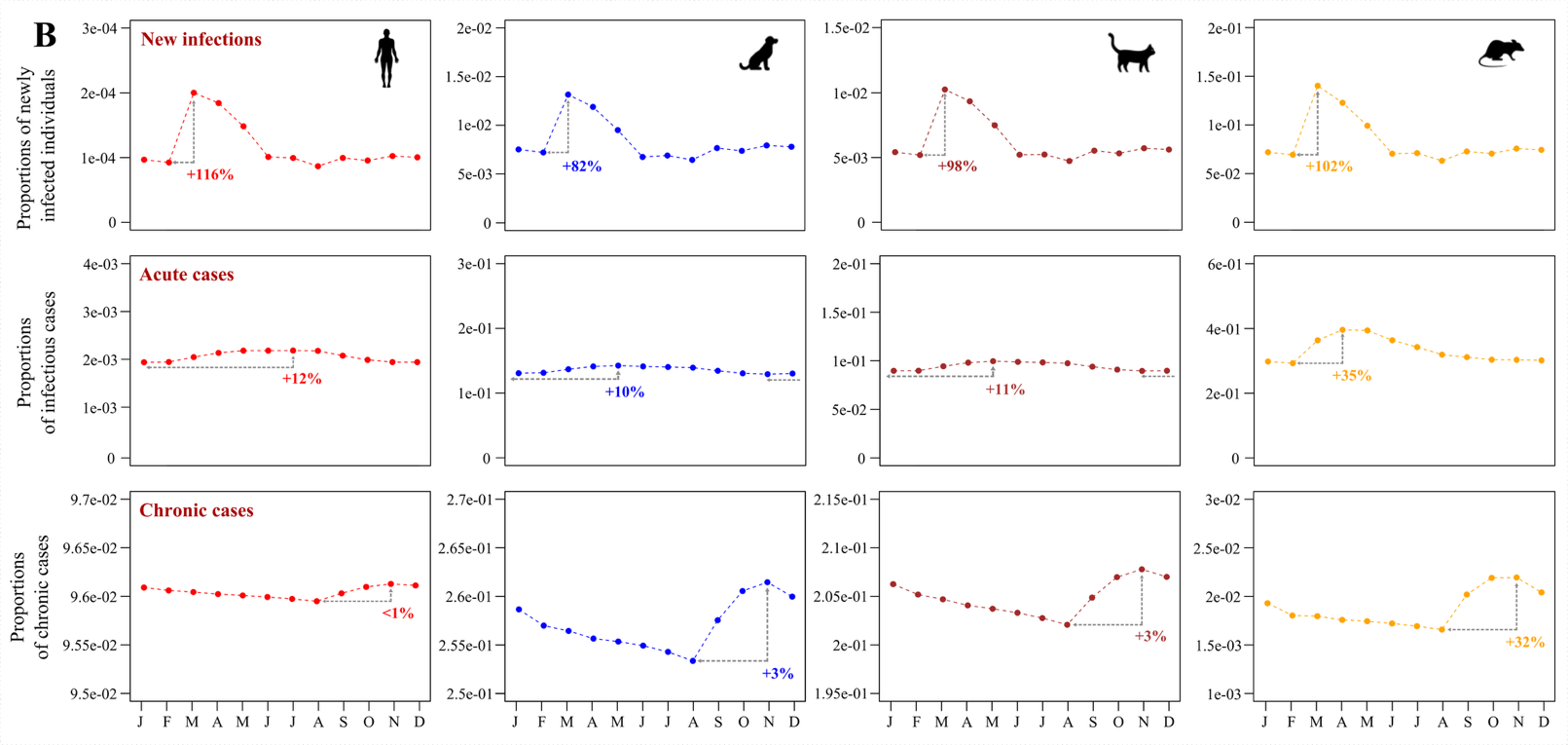
**

**Supporting Information References**

68. Smith JM, Slatkin M. The Stability of Predator‐Prey Systems. Ecology. 1973;54(2):384- 91. <https://doi.org/10.2307/1934346>

69.  Caswell H. Matrix Population Models. Sunderland, MA: Sinauer Associates. 2001.

**Icons**

**Human icon:** “https://www.flaticon.com/free-icons/human” title="human icons">Human icons created by Freepik - Flaticon

**Dog icon:** “https://www.flaticon.com/free-icons/dog” title="dog icons">Dog icons created by PLANBSTUDIO - Flaticon

**Cat icon:** “https://www.flaticon.com/free-icons/cat” title="cat icons">Cat icons created by Victoruler - Flaticon

**Rodent icon:** “https://www.flaticon.com/free-icons/rodent” title="Rodent icons" > Rodent icons created by Freepik - Flaticon

**Chicken icon:** “https://www.flaticon.com/free-icons/bird” title="bird icons" > Bird icons created by Freepik - Flaticon
