# Supplementary material for: Seasonal variations of Triatoma dimidiata demography and Trypanosoma cruzi transmission within its multi-host community in the Yucatan Peninsula, Mexico: Insights from an integrative SIR eco-epidemiological modelling
Source: PLoS Negl Trop Dis. 2026 Jul 15;20(7):e0014500. doi: 10.1371/journal.pntd.0014500 (PMC13384399; doi:10.1371/journal.pntd.0014500)
Supplement: S1 File — (DOCX) [file pntd.0014500.s002.docx]

**Human icon:** “https://www.flaticon.com/free-icons/human” title="human icons">Human icons created by Freepik - Flaticon

**Dog icon:** “https://www.flaticon.com/free-icons/dog” title="dog icons">Dog icons created by PLANBSTUDIO - Flaticon

**Cat icon:** “https://www.flaticon.com/free-icons/cat” title="cat icons">Cat icons created by Victoruler - Flaticon

**Rodent icon:** “https://www.flaticon.com/free-icons/rodent” title="Rodent icons" > Rodent icons created by Freepik - Flaticon

**Chicken icon:** “https://www.flaticon.com/free-icons/bird” title="bird icons" > Bird icons created by Freepik - Flaticon
