# Supplementary material for: Seasonal variations of Triatoma dimidiata demography and Trypanosoma cruzi transmission within its multi-host community in the Yucatan Peninsula, Mexico: Insights from an integrative SIR eco-epidemiological modelling
Source: PLoS Negl Trop Dis. 2026 Jul 15;20(7):e0014500. doi: 10.1371/journal.pntd.0014500 (PMC13384399; doi:10.1371/journal.pntd.0014500)
Supplement: Attachment — Submitted filename: PNTD-D-25-01227.pdf [file pntd.0014500.s003.pdf]

PLOS Neglected Tropical Diseases

Seasonal variations of Triatoma dimidiata demography and Trypanosoma cruzi transmission within its multi-host community in the Yucatan peninsula, Mexico: insights from an integrative SIR eco-epidemiological modelling

--Manuscript Draft--

**Manuscript Number:**	PNTD-D-25-01227
**Full Title:**	Seasonal variations of Triatoma dimidiata demography and Trypanosoma cruzi transmission within its multi-host community in the Yucatan peninsula, Mexico: insights from an integrative SIR eco-epidemiological modelling
**Short Title:**	Modelling seasonal variations of Chagas disease transmission
**Article Type:**	Research Article
**Keywords:**	SIR model; Integrative approach; Metabarcoding; Chagas disease; Triatoma dimidiata; Trypanosoma cruzi
**Abstract:**	Chagas disease is a zoonotic disease affecting 7 million people worldwide, mostly in Latin America. The causative agent, Trypanosoma cruzi, is transmitted by triatomine vectors feeding on a high diversity of vertebrate species. To understand its complex transmission dynamics is critical to assess disease risk and potential control strategies. We expanded a previous SI model of T. cruzi transmission in a typical synanthropic and domesticated host community into a SIR model to account for the effect of seasonal variations in the stage-structured vector demography and for the within-host parasite dynamics. The model was parameterized by using data on Triatoma dimidiata and hosts populations accumulated over the past 20 years in villages of the Yucatan peninsula, Mexico. Our modelling successfully reproduced 86.5% of the seasonal variations in vector abundance typically observed in those villages, with a peak of abundance during the warm and dry season (March to May), and it provided the first predictions of seasonal variations in the local T. cruzi transmission. Along with their abundance, vector infection was anticipated to rise by 23% from March to June, which closely matches a 19% increase in the field and led to a higher risk of T. cruzi transmission to all competent hosts. During this 3-months period, we predicted a two-fold increase in the incidence of T. cruzi, so that 39% of the yearly new infections occurred. Finally, extensive sensitivity analyses provided evidence that reducing by 50% the current abundance of dogs, rodents, and increasing the current abundance of avians by 50% could reduce T. cruzi yearly incidence by up to ~45%, ~150%, and ~50%, respectively, confirming the potential of zooprophylactic control strategies. These findings strongly support the implementation of educational and empowerment strategies embedded in One Health approach to sustainably interrupt Chagas disease intra-domiciliary transmission.
**Additional Information:**	
**Question**	**Response**
**Government Employee**<br><br>Are you or any of the contributing authors an employee of the United States government?<br><br>Manuscripts authored by one or more US Government employees are not copyrighted, but are licensed under a CC0 Public Domain Dedication, which allows unlimited distribution and reuse of the article for any lawful purpose. This is a	No - No authors are employees of the U.S. government.

Powered by Editorial Manager® and ProduXion Manager® from Aries Systems Corporation

legal requirement for US Government employees.<br><br>This will be typeset if the manuscript is accepted for publication.	
**Financial Disclosure**<br><br>Enter a financial disclosure statement that describes the sources of funding for the work included in this submission. Review the submission guidelines for detailed requirements. View published research articles from *PLOS Neglected Tropical Diseases* for specific examples.<br><br>This statement is required for submission and **will appear in the published article** if the submission is accepted. Please make sure it is accurate.<br><br>**Funded studies**<br>Enter a statement with the following details:<br>• Initials of the authors who received each award<br>• Grant numbers awarded to each author<br>• The full name of each funder<br>• URL of each funder website<br>• Did the sponsors or funders play any role in the study design, data collection and analysis, decision to publish, or preparation of the manuscript?<br><br>Did you receive funding for this work?	Yes
Please add funding details.<br>as follow-up to "**Financial Disclosure**<br><br>Enter a financial disclosure statement that describes the sources of funding for the work included in this submission. Review the submission guidelines for detailed requirements. View published research articles from *PLOS Neglected Tropical Diseases* for specific examples.<br><br>This statement is required for submission and **will appear in the published article** if	This work received financial support from CONACYT (National Council of Science and Technology, Mexico) – PCC (319746) and from the French National Research Institute for Sustainable Development (IRD, France). This study is set within the framework of the 'Laboratoires d'Excellences (LABEX)' TULIP (ANR-10-LABX-41) and of the 'École Universitaire de Recherche (EUR)' TULIP-GS (ANR-18-EURE-0019). Funders did not play any role in the study design, data collection and analysis, decision to publish, or preparation of the manuscript.

Powered by Editorial Manager® and ProduXion Manager® from Aries Systems Corporation

the submission is accepted. Please make sure it is accurate.<br><br>**Funded studies**<br>Enter a statement with the following details:<br>• Initials of the authors who received each award<br>• Grant numbers awarded to each author<br>• The full name of each funder<br>• URL of each funder website<br>• Did the sponsors or funders play any role in the study design, data collection and analysis, decision to publish, or preparation of the manuscript?<br><br>Did you receive funding for this work?"	
Please select the country of your main research funder (please select carefully as in some cases this is used in fee calculation).<br>as follow-up to "**Financial Disclosure**<br><br>Enter a financial disclosure statement that describes the sources of funding for the work included in this submission. Review the submission guidelines for detailed requirements. View published research articles from *PLOS Neglected Tropical Diseases* for specific examples.<br><br>This statement is required for submission and **will appear in the published article** if the submission is accepted. Please make sure it is accurate.<br><br>**Funded studies**<br>Enter a statement with the following details:<br>• Initials of the authors who received each award<br>• Grant numbers awarded to each author<br>• The full name of each funder<br>• URL of each funder website<br>• Did the sponsors or funders play any role in the study design, data collection and analysis, decision to publish, or preparation of the manuscript?	MEXICO - MX

Did you receive funding for this work?"	
**Competing Interests**<br><br>On behalf of all authors, disclose any competing interests that could be perceived to bias this work.<br><br>This statement will be typeset if the manuscript is accepted for publication.<br><br>Review the instructions link below and PLOS NTDs' competing interests policy to determine what information must be disclosed at submission.	The authors have declared that no competing interests exist.
**Data Availability**<br><br>Provide a **Data Availability Statement** in the box below. This statement should detail where the data used in this submission can be accessed. This statement will be typeset if the manuscript is accepted for publication.<br><br>Before publication, authors are required to make all data underlying their findings fully available, without restriction. Review our PLOS Data Policy page for detailed information on this policy. Instructions for writing your Data Availability statement can be accessed via the Instructions link below.	All relevant data are within the manuscript and its Supporting Information files.

Click here to access/download;Manuscript;Zitoun et al. Manuscript.pdf

Seasonal variations of *Triatoma dimidiata* demography and *Trypanosoma cruzi* transmission within its multi-host community in the Yucatan peninsula, Mexico: insights from an integrative SIR eco-epidemiological modelling

Jean-Loup Zitoun[1], Eric Dumonteil[2], Julio Vladimir Cruz-Chan[1], Etienne Waleckx[1,3], and Sébastien Gourbière[4,5*]

[1] Laboratorio de Parasitología, Centro de Investigaciones Regionales "Dr. Hideyo Noguchi", Universidad Autonoma de Yucatán, Mérida, Yucatán, México.

[2] Department of Tropical Medicine and Infectious Disease, Celia Scott Weatherhead, School of Public Health and Tropical Medicine, and Vector-Borne and Infectious Disease Research Center, Tulane University, New Orleans, Louisiana, USA

[3] Institut de Recherche pour le Développement, UMR INTERTRYP IRD, CIRAD, Université de Montpellier, Montpellier, France.

[4] UMR5096 'Laboratoire Génome et Développement des Plantes', Université de Perpignan Via Domitia, Perpignan, France.

[5] School of Life Sciences, University of Sussex, Falmer, Brighton, United Kingdom.

* Corresponding author: Sébastien Gourbière, PhD

Université de Perpignan Via Domitia

52 Av. Paul Alduy, 66100 Perpignan, France

Tel: 33 (0)44 30 19 81 18

Email: sebastien.gourbiere@univ-perp.fr

ABSTRACT

Chagas disease is a zoonotic disease affecting 7 million people worldwide, mostly in Latin America. The causative agent, *Trypanosoma cruzi*, is transmitted by triatomine vectors feeding on a high diversity of vertebrate species. To understand its complex transmission dynamics is critical to assess disease risk and potential control strategies. We expanded a previous SI model of *T. cruzi* transmission in a typical synanthropic and domesticated host community into a SIR model to account for the effect of seasonal variations in the stage-structured vector demography and for the within-host parasite dynamics. The model was parameterized by using data on *Triatoma dimidiata* and hosts populations accumulated over the past 20 years in villages of the Yucatan peninsula, Mexico. Our modelling successfully reproduced 86.5% of the seasonal variations in vector abundance typically observed in those villages, with a peak of abundance during the warm and dry season (March to May), and it provided the first predictions of seasonal variations in the local *T. cruzi* transmission. Along with their abundance, vector infection was anticipated to rise by 23% from March to June, which closely matches a 19% increase in the field and led to a higher risk of *T. cruzi* transmission to all competent hosts. During this 3-months period, we predicted a two-fold increase in the incidence of *T. cruzi*, so that 39% of the yearly new infections occurred. Finally, extensive sensitivity analyses provided evidence that reducing by 50% the current abundance of dogs, rodents, and increasing the current abundance of avians by 50% could reduce *T. cruzi* yearly incidence by up to ~45%, ~150%, and ~50%, respectively, confirming the potential of zooprophylactic control strategies. These findings strongly support the implementation of educational and empowerment strategies embedded in One Health approach to sustainably interrupt Chagas disease intra-domiciliary transmission.

AUTHOR SUMMARY

Chagas disease is caused by the parasite *Trypanosoma cruzi* that predominantly spreads through the faeces of infected triatomine hematophagous vectors. There currently are 7 millions people infected worldwide, mostly in Latin America and, in the absence of vaccine, the main strategy to prevent Chagas disease remains to limit contacts with vectors through insecticide spraying, house and backyard management and/or the use of screens. Epidemiological models are essential tools to understand the complex dynamics of *T. cruzi* transmission through the large network of species which the bugs feed on, and to assess the potential of putative control strategies. By combining such models and data collected over the last 20 years in the Yucatan peninsula in Mexico, we show that there are seasonal variations in vector abundance and infection by the parasite that result in a 3-months period with twice as much parasite transmission as during the rest of the year. We further show that the size of avian, rodent and dog populations in villages can substantially modify the level of *T. cruzi* transmission to humans, which confirms that managing the host community offers a complementary way of controlling Chagas disease.

INTRODUCTION

Vector-borne infectious diseases remain a critical public health challenge threatening over 80% of the world population [1] and accounting for 17% of the global estimated burden of all infectious diseases [2]. About half of the twenty Neglected Tropical Diseases (NTDs) are caused by vector-transmitted infectious agents [3] and, together with the directly- or environmentally-transmitted NTDs, are responsible for high levels of morbidity [4], affecting particularly the poorest populations living in the tropics and sub-tropics [5]. The lack of investments into NTDs research clearly impedes the development of an integrated knowledge of their biology, ecology and evolutionary potential, thereby limiting our ability to anticipate the efficacy of potential control strategies. Such a conundrum is especially difficult to overcome for vector-borne zoonotic NTDs whose spread, establishment and potential (re-)emergence entail complex interactions between pathogens, vectors, hosts, and their environments [6].

Chagas disease, also known as American trypanosomiasis, is a complex zoonotic NTD caused by the protozoan *Trypanosoma cruzi*. This NTD is transmitted to humans and to more than 150 species of domestic and wild mammals [7] through the contact with infected feces of hematophagous insects known as triatomines [8-9]. Three key features of *T. cruzi* transmission are known to shape the disease distribution and the success of its control. First, while triatomines feed on a vast diversity of vertebrate species, the susceptibility of those species to *T. cruzi* infection varies greatly. Some reservoir species, most notably dogs and rodents, increase the risk of human infections to such an extent that they can become the targets of control interventions [10-12], while an increase of the abundance of refractory avian species can result in a reduction of *T. cruzi* transmission to humans [13-14]. Second, the within-host dynamics of *T. cruzi* underlies individual heterogeneities in host infectiousness [15-16]. After a primary infection, the parasite load in host's blood increases until the infected individual becomes infectious for susceptible vectors during bloodmeals. This acute stage is typically followed by a long-term chronic phase characterized by a low parasitemia, whereby a host becomes much less infectious [17-18]. Third, the abundance and population structure of triatomine vectors can exhibit strong seasonal variations (e.g. [19-20]) that could interact with density-dependence mechanisms influencing vectorial transmission. Indeed, individual vector feeding frequency has been shown to decrease with vector density as a result of intraspecific interference competition associated with an increase in triatomine biting perception that trigger defensive behavioural responses from their hosts [14, 21-23]. Characterizing the interplay between the diversity of host species, the within-host dynamics of *T. cruzi*, and the density-dependent mechanisms influencing vector seasonal demography and transmission

remains a significant challenge. The integration of these key elements into well empirically parameterized eco-epidemiological models of *T. cruzi* transmission can undoubtedly provide valuable insights into their impacts on the temporal dynamics of the risk of human infection, and the efficiency of potential control interventions.

The Yucatan peninsula has experienced an increasing transmission of vector-borne and zoonotic diseases since the 1960s [24] under the influence of anthropogenic habitat modification and land use changes [25]. The transmission of *T. cruzi* has been extensively studied in this area, where it is known to cause a 1-5% prevalence of Chagas disease in local populations [26], and to display the three main features described above. A recent local comparative analysis indeed showed that *T. cruzi* is infecting the largest number of vertebrate species among all vector-borne pathogens circulating in the wild fauna [27], and diverse host species have been shown to affect vector house infestation [28] and/or to be able to amplify or dilute *T. cruzi* transmission within the host community typically found in a village [14]. Meanwhile, ecological field studies focused on the demography of the local vector species, i.e. *Triatoma dimidiata*, have documented strong seasonal variations in vector abundance with a marked peak during the warm and dry season (March to May) and a typically low intra-domiciliary presence during the rest of the year [19, 29-31]. Those empirical studies allowed to design *T. dimidiata* population dynamic models at the house [32] and village [33] scales, which provided key insights into the strategies to be implemented to control the corresponding entomological risks [34-35]. More recently, Flores-Ferrer et al. [14] designed the first eco-epidemiological model of *T. cruzi* transmission through its local host community that explicitly accounted for the impact of triatomines intraspecific competition on both vector demography and parasite transmission. While this model provided key quantitative assessments of the local role of various host species in *T. cruzi* transmission, and of the potential efficacy of zooprophylaxis strategies, it did not take into account the chronic infection status of the hosts and the seasonal variations in vector demography.

In this contribution, we strengthened this modelling of *T. cruzi* transmission in a multi-host community by relaxing those two simplifying assumptions to build an eco-epidemiological model accounting for the infectious status of the hosts and for the interaction between density-dependence mechanisms and seasonality throughout the stage-structured vector life cycle. Such an improved modelling is intended to i) provide the first quantitative description of the local seasonal patterns in *T. cruzi* transmission and its incidence in humans, and to ii) refine our assessment of the role of the various host species in the transmission of *T. cruzi* and of the potential of zooprophylaxis to mitigate disease risk.

MATERIAL AND METHODS

General approach

We extended the compartmental Susceptible (S) - Infectious (I) model developed by Flores-Ferrer et al. [14] into an eco-epidemiological model allowing to predict the seasonal variations in i) *T. dimidiata* stage-structured demography and ii) *T. cruzi* transmission through the host community typically encountered in villages of the Yucatan peninsula, Mexico (See 'Modelling *T. cruzi* transmission in structured hosts and vector populations of the Yucatan peninsula' below). We adapted our previous modelling by accounting for two additional structures of the vector and hosts populations. First, each infected host population was partitioned into 'infectious' and 'recovered' (R) individuals corresponding to hosts in the acute and chronic phases of the infection, in order to take into account that the low parasitemia during chronic infection typically reduces *T. cruzi* transmission to susceptible vectors [17-18, 36]. Second, the vector population was structured into 7 stages (eggs, nymphs 1 to 5 and adults) to provide a description of the relationship between *T. dimidiata* demography and its stage-dependent blood-feeding behaviour. We parameterized this SIR eco-epidemiological model by taking advantage of long-term field and experimental studies of the local host-vector-parasite system that included estimates of vector and hosts demographic rates and of their prevalence of infection by *T. cruzi*, along with a detailed metabarcoding description of the triatomine blood-feeding behavior (See 'Vector feeding preferences' below). We then used this parameterized modelling to make predictions about the seasonal variations in vector abundance and *T. cruzi* transmission within its local synanthropic and domesticated hosts community, and to assess the potential efficacy of zooprophylactic interventions based on the manipulation of this local host community.

Modelling *T. cruzi* transmission in structured hosts and vector populations

Following Flores-Ferrer et al. [14], our SIR model of *T. cruzi* transmission (Fig. 1) includes four competent hosts; humans (h=1), dogs (h=2), cats (h=3), rodents (h=4) and non-competent avian hosts (h=5) that, altogether, account for up to 89% of all bloodmeals typically made by *T. dimidiata* within villages of the Yucatan peninsula [37-38]. For each competent host species, individuals are either susceptible (S_h), infectious (I_h) or 'recovered' (R_h), where the latter two categories correspond to the acute and chronic phases of the infection (as in [17]). Refractory hosts (avians) (R_5) are then classified as recovered in the model as they are not competent to transmit *T. cruzi* to susceptible vectors. All hosts individuals are considered to die at a species-specific per-capita mortality rate d_h, and such deaths

are balanced by the recruitment of susceptible individuals at a constant rate B_h. The adult vector population is made of susceptible (S_A) and infectious (I_A) individuals that die at a natural per-capita mortality rate d_A, and their death is balanced by the immigration of M_S susceptible and M_I infected individuals dispersing from the sylvatic habitat and by their reproduction within the village. As in the original modelling [14], adult females produce susceptible eggs according to the amount of ingested blood (e.g. [29]). The fertility of an adult female was then modelled as the product of the fertility per blood meal b_A and the per adult vector blood-feeding rate $\beta_A(\boldsymbol{A}, \boldsymbol{H})$ that accounts for the intraspecific competition between the adult vectors ($\boldsymbol{A}$) aiming to feed on host individuals of all species ($\boldsymbol{H}$). After eggs hatch into first stage nymphs with a constant probability s_e, each nymphal stage develops into the next-one, and ultimately into adults, according to probabilities $s_i(\boldsymbol{N},\boldsymbol{H})$ (i=1…5) that decrease with respect to the competition between nymphs of all stages ($\boldsymbol{N}$) for blood-feeding onto all host species ($\boldsymbol{H}$). In such a stage-structured vector population, both nymphs and adults of *T. dimidiata* can get infected by and transmit *T. cruzi* to any of the competent host species. The *T. cruzi* transmission rates between nymphal and adult vectors and hosts were then set according to stage-specific (i.e. nymphal and adult) vector's i) blood-feeding rates $\beta_A(\boldsymbol{A}, \boldsymbol{H})$ and $\beta_i(\boldsymbol{N}, \boldsymbol{H})$ (i=1…5), and ii) proportions of bloodmeals made on the different competent host species ϕ_{Ah} and ϕ_{Nh} (h=1…5), while considering distinct (per potentially infectious contact) probabilities of *T. cruzi* transmission from any infectious host to a susceptible vector (p_V) and from an infectious vector to a susceptible host h (p_h). Following initial infection by *T. cruzi*, susceptible hosts develop an infectious acute phase which typically lasts for about 5 months, as observed from xenodiagnostic data collected on dogs and rodents [18,36] until the decrease of parasitemia to undetectable levels, characterizing the non-infectious chronic phase.

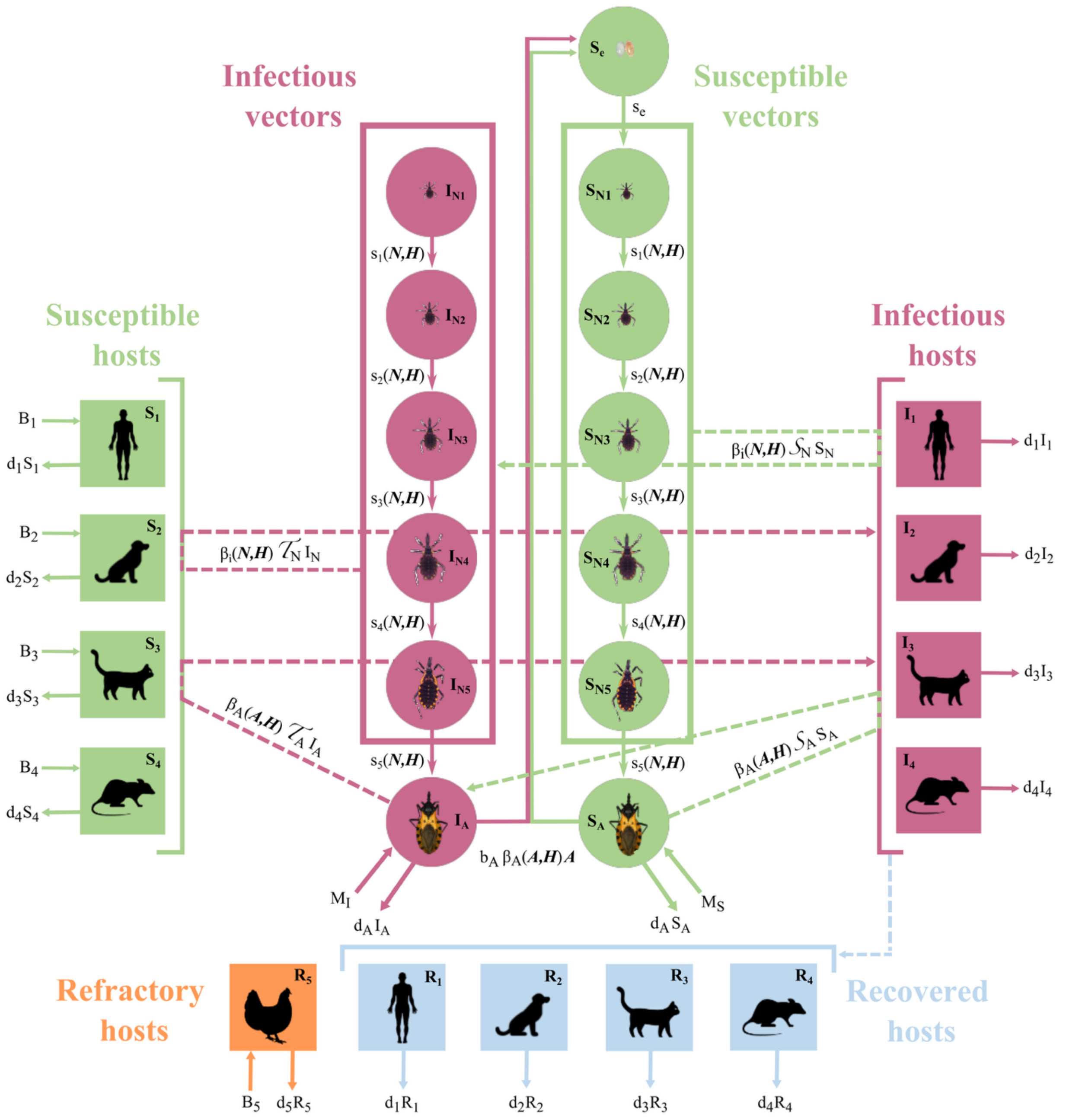

Fig 1. Schematic representation of the SIR model for *T. cruzi* transmission by *T. dimidiata* vectors in a multi-host community. Vectors infected (red) or not (green) by *T. cruzi* can consume blood from humans, dogs, cats, rodents and avian hosts that are susceptible (green), infectious (red), recovered (blue) or refractory (orange) to the parasite infection, with a transition from the infected to recovered status in hosts that takes 5 months. Full arrows then represent the demographic processes of hosts and vectors while dashed arrows correspond to the infection processes.

The details of mathematical equations describing all the demographic and transmission processes represented in Figure 1 are provided in Supplementary Text 1. The model parameters describing those processes are listed in table 1 along with their estimates that were derived according to the methodology and data described below.

Model parameterization to the *T. dimidiata* - *T. cruzi* - domesticated and synanthropic host community

Hosts demography

The average size of the human population (N_1) and number of households encountered in the villages of the Yucatan peninsula, Mexico, i.e. Sudzal, Teya and Bokoba, where the transmission of *T. cruzi* through its local vector and host species has been investigated over the last 20 years, were set to 2011 and 594 according to the last regional census [39]. The abundance of dogs (N_2), cats (N_3), rodents (N_4) and avian (N_5) hosts were estimated as the product of i) their average per household, that were previously found to be equal to 1.22, 1.01, 5.86 and 5.84 from a field survey performed on 308 houses [40] (Dumonteil, personal communication), and ii) the average number of households. The death rate of each host species h (d_h) was calculated as the inverse of its average lifetime expectancy [17], with values for human, dogs, cats, rodents and avian hosts set to 70 years, 3 years, 4 years, 4 months and 6 months, respectively. Such lifetime expectancies were all derived from Flores-Ferrer et al. [14], but for rodents as it was then tailored according to the study by Panti-May et al. [41] on mouse demography. The recruitment rate of host species h (B_h) was then estimated by assuming that the number of hosts of each species (N_h) correspond to its population dynamic equilibrium between births and deaths, which can be shown to be $N_h^* = \frac{B_h}{d_h}$, so that B_h is merely equals to $d_h * N_h$. All the N_h, B_h, and d_h estimated values are provided in the sub-section 'Host demography' of Table 1.

Vectors demography

The population dynamics of non-domiciliated *T. dimidiata* within the villages of the Yucatan peninsula is shaped by adult dispersal and stage-structured demography typical of hemimetabolous insects. We used its long-standing field and modelling studies to estimate the corresponding parameters as follow.

Adult dispersal and reproduction. The abundance of adult vectors dispersing into the village of the Yucatan peninsula shows a strong seasonal pattern with a peak from March to May [42] that has already been quantified [33] along with their *T. cruzi* prevalence [31, 37-38], which provided estimates for the number of susceptible (M_S) and infected (M_I) migrating adults. To model the reproduction of (migrating and non-immigrating) adults within the village, we accounted for their sex ratio and its monthly variations that were observed from March 2017 to July 2019 in the villages of Teya, Sudzal and Chumbec, by Moo-Millan [38]. Since the monthly proportions of females did not vary significantly from one year to another ($\chi^2 = 304.5$, df = 297, p = 0.3698) (Sup Fig. 1), we used their

average estimates that confirmed the previously identified female-biased sex-ratio [29], with a proportion of females in the population varying from 48% (± 20%) in January to a maximum of 77% (± 8%) in July. The egg production by *T. dimidiata* females was set as the product of the per adult blood-feeding rate $\beta_A(A, H)$ and the fertility per blood meal (b_A). The former was modelled as in Flores-Ferrer et al. [14] to allow for the maximum per adult blood feeding rate (β_{Amax}) to decrease according to the vector to host abundance ratio ($\boldsymbol{A}/\boldsymbol{H}$) and the intensity ($\gamma_A$) of the vector intraspecific competition for bloodmeals (See Appendix 1). The maximum per adult vector blood-feeding rate (β_{Amax}) was considered to correspond to the maximum number of different host DNA observed in metabarcoding studies of the adult vector gut content [37-38]. As a matter of fact, host DNA are known to remain detectable for 30 days in the digestive system of triatomine vectors [43], a duration which correspond to the time step of our model, so that the maximum number of detected meals provided a sensible estimate of the β_{Amax} equals to 5. This estimate was then adjusted to account for its expected monthly variations with respect to the seasonal patterns previously observed by Catalá [44]. Meanwhile, the intensity of intraspecific competition (γ_A) was set to 0.02 so that the adult blood feeding rate is halved for a vector to host abundance ratio ($\boldsymbol{A}/\boldsymbol{H}$) of 50:1, as previously shown by Pelosse et al. [23]. The fertility per blood meal (b_A) was calculated using data from Zeledón et al. [45] showing that *T. dimidiata* females lay on average 1088 eggs over a 726 days period. Considering the experimentally observed *T. dimidiata* maximum blood-feeding rate of ~5 meals per month [37-38], the fertility per blood meal can be estimated to be 1088/(5*(726/30)) = 9 eggs per meal under laboratory conditions. This value was further reduced according to a previous estimate of a 40% reduction in *T. dimidiata* fertility in the field as compared to its value observed in laboratory colonies, which was found to be highly consistent with a 41% reduction in blood volume consumed by insects collected in villages as compared to laboratory-raised females [29].

Stage-specific development and mortality rates. The duration of eggs and nymphal stages were set according to *T. dimidiata* cohort studies ([46], p.65), where the egg and first nymphal stages were found to last for ~1 month, while the second to fourth nymphal stages lasted ~2 months and the last stage took ~3 months before individuals become adults. The probability of successful development for each nymphal stage (s_i) were set to their maximum values derived in laboratory conditions by Nogueda-Torres et al. [47], which are reported in table 1, and adjusted with respect to their stage specific blood-feeding rate $\beta_i(\boldsymbol{N}, \boldsymbol{H})$ as those are known to influence survival and development of nymphs [46, 48]. The stage specific blood-feeding rates were then modeled using the same density-dependent function as we used for the adults. The value of the intensity of competitive interactions between nymphs for access to hosts (γ_N) was indirectly estimated for the modelled adult vector

population size to reach 800000 individuals, as predicted by Barbu et al. [33] for the village of Teya. This led to an estimate of γ_N = 0.0055 corresponding to a ~4 times lower intensity of competition than for adults, with a reduction by half of the maximal feeding rate for a vector to host abundance ratio of 182:1. The adult mortality was assessed by using insect field collection conducted over a 3-year study [37-38] that showed their monthly death rate to be higher from March to August, i.e. d_A=0.65, than from September to February, i.e. d_A=0.372. Such estimates correspond to an adult life expectancy of ~1.5 and ~3 months, respectively, which are very close to the previous 2.5 months estimate derived from the same vector populations of the Yucatan peninsula [32].

Vectors feeding preferences

We estimated the blood-feeding rates made by adults (α_{Ah}) and nymphs (α_{Nh}) on all host types h (h=1…5) using a meta-analysis of *T. dimidiata* bloodmeal metabarcoding studies achieved in villages of the Yucatan peninsula over the last 10 years [37-38], and the procedure developed and implemented in Flores-Ferrer et al. [14]. The meta-analysis provided an estimate of the proportion of bloodmeals taken by infected and non-infected adults and nymphs in both domiciles and backyards (Sup. Tab 1). Since ~95% of the 243 collected adults were found in domiciles where no significant feeding differences between infected and non-infected individuals were observed (χ^2 =3.443, df = 4, p =0.487), we ultimately pooled those data to estimate the average proportions φ_{Ah} of adult bloodmeals made on host types h (h=1…5). Adults vectors typically fed on humans, rodents and dogs, representing 54%, 21% and 18% of bloodmeals, while avian hosts and cats accounted for 4% and 3% of bloodmeals, respectively. The total number of collected nymphs was much lower, i.e. 27, with ~82% of them found in the backyards and none being infected. Therefore, we again pooled the data to estimate the average proportions φ_{Nh} of nymphal bloodmeals made on host types h (h=1…5). Nymphs fed less on humans, rodents, dogs and cats, representing only 37%, 16%, 14% and 2% of bloodmeals, while more than 30% of their bloodmeals were taken on avian hosts. Those differences between the composition of adults and nymphs bloodmeals (χ^2 =43.217, df = 4, p = 9e-09) are clearly associated with their spatial partition between houses and backyards. From those estimates of the proportions of bloodmeals taken by adult (ϕ_{Ah}) and nymphs (ϕ_{Nh}) on host types h (h=1…5), we derived the vector blood-feeding rates of adults (α_{Ah}) and nymphs (α_{Nh}) following Flores-Ferrer et al. [14] to account for the impact of the host populations sizes (N_h) on the observed composition of bloodmeals.

Transmission probabilities of *T. cruzi*

The probability of *T. cruzi* transmission from an infected host to a susceptible vector (p_V) was set according to its previous estimate by Rascalou et al. [17]. The probability of *T. cruzi* transmission (p_h) from an infected vector to the different host types h (h=1…5), were estimated by fitting the model predictions to the observed prevalence of *T. cruzi* in the host populations. The observed prevalences of infection were taken from previous field studies located in villages of the Yucatan peninsula, where they were found to be 2.3% in humans (9/390) [49], 9.8% in dogs (10/102) [50], 8.6% in cats (19/220) [51] and 9.9% in rodents (57/575) [52]. The corresponding values of the host to vector probabilities of transmission (*ph*) were found to be very similar with their previous estimates [14] and allowed to complete Table 1 and the parameterization of our model.

Symbol	Name	Units	Value	References
Host demography				
N_h	Number of host type h	ind		
	Humans, Dogs, Cats, Rodents, Avians		2011, 727, 601, 3481, 3471	[39-40]
B_h	Recruitment rate of host type h	ind. month^{-1}		
	Humans, Dogs, Cats, Rodents, Avians		2.394, 20.194, 12.521, 870.25, 578.5	[14,41], This study
d_h	Death rate of host type h (10-2)	month^{-1}		
	Humans, Dogs, Cats, Rodents, Avians		0.119 , 2.777, 2.083, 25, 16.666	[14,41], This study
Vector demography				
M_S, M_I	Migrating adult vectors	ind. month^{-1}		
	Susceptible, Infected		45060, 2876	[31,33,37-38]
$p_f(t)$	Proportion of female *T. dimidiata* adults	month^{-1}		
	January, February, March, April, May, June		0.48, 0.54, 0.62, 0.69, 0.70, 0.58	[38]
	July, August, September, October, November, December		0.77, 0.73, 0.70, 0.71, 0.58, 0.55	[38]
β_{Amax}	Maximal vector feeding rate of adults	month^{-1}		
	March, April, May, June, July, August, September - February		2.705, 5, 5, 3.853, 2.705, 1.558, 0.410	[37-38,43-44], This study
γ_A	Density-dependent regulation of adult vectors feeding		0.02	[23]
b_A	Vector fertility per bloodmeal		5.399	[29,45]
s_i	Survival rate of nymphs type i	month^{-1}		
	Nymph 1, Nymph 2, Nymph 3, Nymph 4, Nymph 5		0.863, 0.875, 0.869, 0.924, 0.957	[47]
β_{imax}	Maximal vector feeding rates of nymphs type i	month^{-1}		
	Nymph 1, Nymph 2, Nymph 3, Nymph 4, Nymph 5		1.525, 3.051, 3.313, 2.345, 1.885	[47]
γ_N	Density-dependent regulation of nymph vectors feeding		0.0055	This study
d_A	Adult vectors death rate	month^{-1}		
	March - August, September - February,		0.65, 0.372	[37-38], This study
Vector feeding preferences				
ϕ_{Ah}	Proportions of adult bloodmeals on host type h			
	Humans, Dogs, Cats, Rodents, Avians		0.537, 0.18, 0.029, 0.214, 0.04	[37-38]
ϕ_{Nh}	Proportions of nymph bloodmeals on host type h			
	Humans, Dogs, Cats, Rodents, Avians		0.372, 0.163, 0.023, 0.14, 0.302	[37-38]
α_{Ah}	Adult vector feeding rates on host type h			
	Humans, Dogs, Cats, Rodents, Avians		2.100, 1.947, 0.379, 0.483, 0.091	[14], This study
α_{Nh}	Nymph vector feeding rates on host type h			
	Humans, Dogs, Cats, Rodents, Avians		1.609, 1.951, 0.333, 0.350, 0.757	[14], This study
Transmission probabilities of T. cruzi				
p_v	Probability of vector infection from infected host		0.95	[17]
p_h	Probability of host h infection from vector (10-4)			
	Humans, Dogs, Cats, Rodents, Avians		0.00608, 0.595, 2.15, 35.7, 0	This study

Table 1. Definitions and estimates of the model parameters.

Analysis of the seasonal vector demography and *T. cruzi* transmission dynamics

We first used our well empirically parameterized model to predict the monthly variations in *T. dimidiata* abundance (Fig. 2) and *T. cruzi* transmission (Fig. 3) within the modelled village. Those former predictions were compared to the monthly variations in vector abundance observed in Teya and Sudzal [31] by calculating the coefficient of determination (R^2) between predicted and observed within year temporal distribution of vectors. We then investigated the impact of these monthly variations of *T. dimidiata* abundance on parasite transmission by predicting, for each host, the typical seasonal changes in the number of new infections, infected and recovered individuals (Fig. 4). We further used our parameterized modelling to simulate variations in the abundance of dogs, cats, rodents and avian hosts in the modelled village within a range of -50% to +50% around the estimated values shown in Table 1, and we assessed the resulting changes in *T. cruzi* prevalence of infection among the vector and competent hosts (Fig. 5). Finally, we tested the sensitivity of our predictions about the effects of the host community structure to the duration of the host infectious stage as it has been shown that, depending on *T. cruzi* strain, the parasite load can stay high in host blood for a duration exceeding 5 months [18]. We therefore increased the duration of the acute phase from 5 to 8, 12 and 18 months and re-assessed the role of each host species on *T. cruzi* transmission in all these putative epidemiological conditions (Fig. 6).

RESULTS

Seasonal dynamics of *T. dimidiata* in a village of the Yucatan peninsula

Our parameterized model integrating the observed within-year variations in adult reproduction and survival predicted a strong and stable seasonal pattern of *T. dimidiata* abundance (Fig 2A), with a peak of adult vectors in the March-May period, as repeatedly observed in the Yucatan peninsula [19, 29-31].

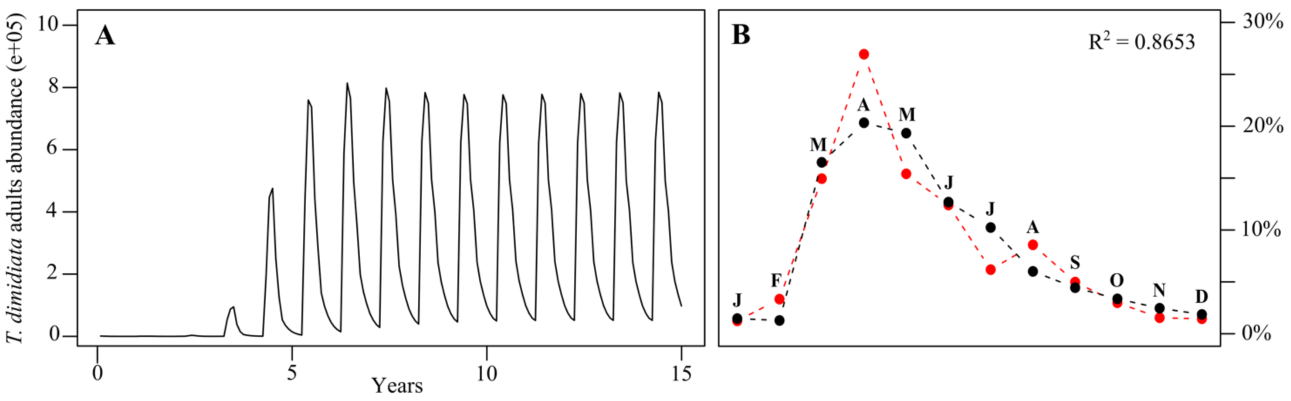

Fig 2. Seasonal variations of *T. dimidiata* abundance in a village of the Yucatan peninsula. The predicted temporal variations of the number of *T. dimidiata* adults are represented in black for a 15-years period (A). The monthly abundance of adults predicted by our modelling (black) and observed by Moo-Millan et al. [31] (red) were found to be highly correlated (B).

The predicted seasonal pattern consistently matched the observed monthly distribution of *T. dimidiata* adult captures in the villages of Teya and Sudzal (Fig. 2B) as it properly reproduced the peak of abundance associated with the warm and dry season (from March to May) as well as the observed decrease in vector abundance during the rainy and northerlies season (from June to February). Interestingly, these variations in vector abundance are expected to translate into changes in their (density-dependent) feeding frequency, which our model was also able to predict. The adult feeding frequency was shown to vary from 1.59 to 2.17 bloodmeals per month during the dry season (March to May) and then to slowly decrease until they consume only 0.25 to 0.33 bloodmeals per month during the rainy and northerlies season, with the latter roughly corresponding to the maximal amount of time between bloodmeals, i.e. 3 to 4 months, that is required for survival [46]. These temporal variations in the number of *T. dimidiata* adults and their frequency of bloodmeals are likely to affect *T. cruzi* transmission to competent hosts. The SIR model was then used to look at the potential seasonal variations in *T. cruzi* prevalence in its vector and host community.

Seasonal dynamics of *T. cruzi* transmission in a village of the Yucatan peninsula

The SIR model, parameterized to fit the average prevalence of *T. cruzi* infection observed in the *T. dimidiata* population and the host community, provided the first quantitative predictions of the existence of seasonal variations in the infection of vectors and competent hosts (Fig. 3A).

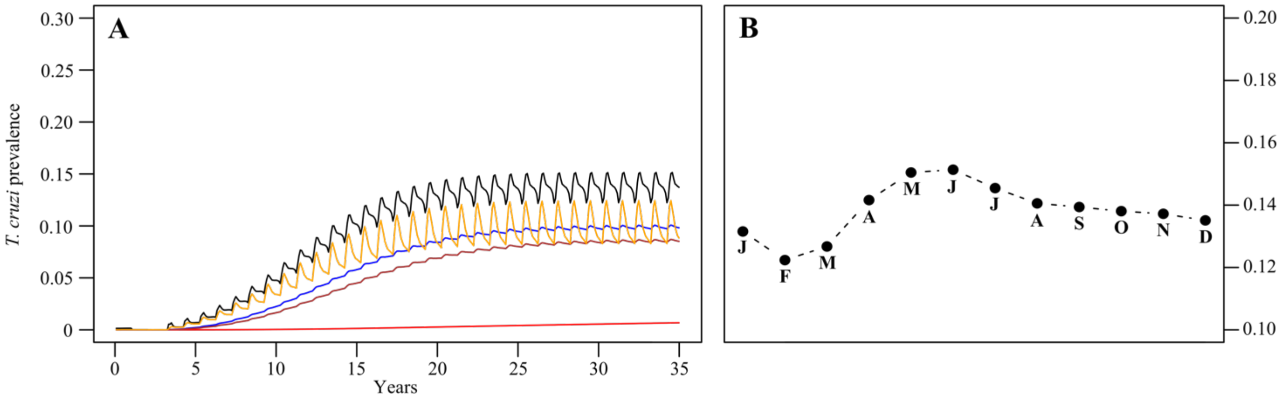

Fig 3. Seasonal variations of *T. cruzi* host and vector prevalences in a village of the Yucatan peninsula. The seasonal variations in the prevalence of infection by *T. cruzi* are represented for humans (red), dogs (blue), cats (brown) and rodents (orange) during a 35-year period (A), and for *T. dimidiata* adults (black) during a 35-year (A) and a 1-year period (B).

Interestingly, the *T. cruzi* infection prevalence in vectors was predicted to vary substantially throughout the year (Fig. 3A, B), with a 23% increase from March to June followed by a slow return to its initial level during the rainy and northerlies season. Although such variations of *T. cruzi* prevalence were also predicted in the different competent hosts (Fig. 3A), as expected, the corresponding oscillations were of smaller amplitude for hosts with typically long-life expectancies. In fact, rodent prevalence rose by 50% between February and May, while, during the same period, prevalence of infection only increased by 4%, 3% and <1% in dogs, cats and humans, respectively. The increase in triatomine abondance and the resulting variations in *T. cruzi* prevalences throughout the year, clearly called for additional predictions to identify periods with a high risk of transmission, i.e. months of the year when the incidence (number of new cases) is expected to be higher.

Seasonal variations in the incidence of *T. cruzi* infection, and in the number of acute and chronic cases.

Our SIR model predicted that the warm and dry season (March to May) represents a crucial period for *T. cruzi* transmission with a high risk of infection for all the competent hosts (Fig 4A-D) since 39% of the yearly new infections (across all hosts) occur during these 3 months.

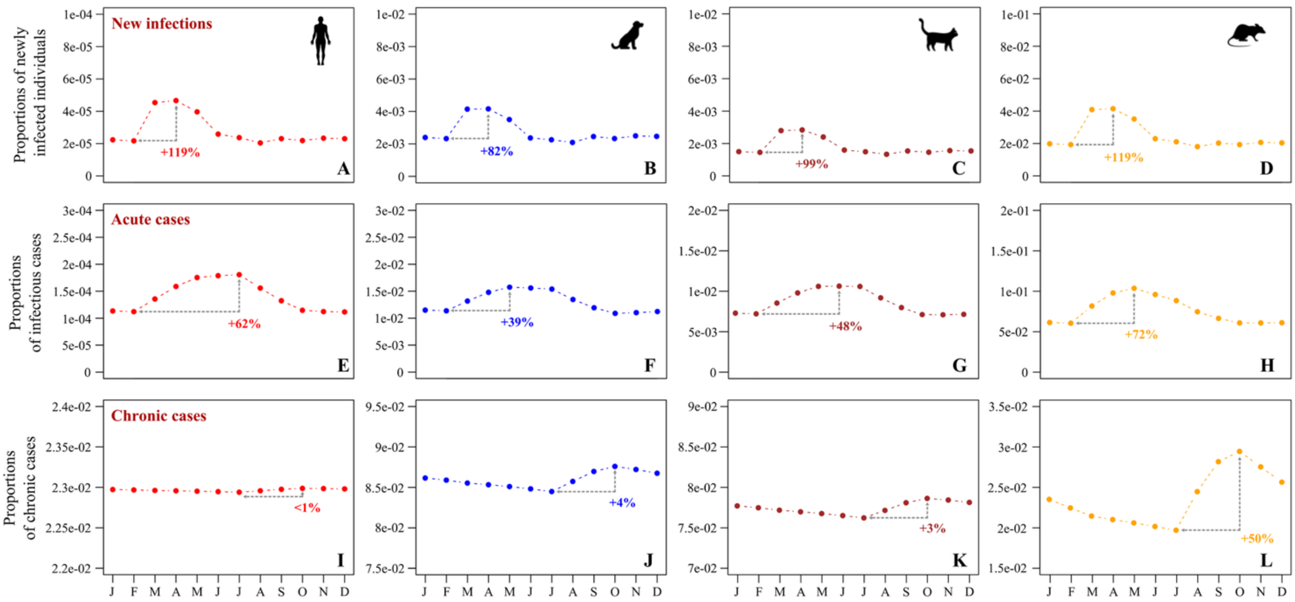

Fig 4. Seasonal variations of *T. cruzi* incidence and acute-chronic cases in a village of the Yucatan peninsula. The monthly variations in the proportions of newly infected individuals (A-D), infectious (E-H) and chronic (I-L) cases measured among the entire population of humans (red), dogs (blue), cats (brown), rodents (orange) are represented for a 1-year period.

The number of new infections varied importantly between hosts, with 0.68, 24, 13, 1042 cases per year for humans, dogs, cats and rodents, respectively, and their seasonal increase during the warm and dry season was predicted to be more pronounced for humans and rodents (+119%) than for cats (+99%) and dogs (+82%) (Fig. 4A-D). Following the transmission of *T. cruzi*, these new infected individuals enter an acute phase where the parasite load quickly increases in the host blood. As expected, given the 5-months duration of this acute phase, the peak of acute cases in each host type was broader and lasted until October, when the proportion of such cases returns to initials levels (Fig. 4E-H). The rise in the proportion of acute cases among hosts mimicked the seasonal pattern observed for new infections in humans (+62%), rodents (+72%), dogs (+39%) and cats (+48%) (Fig. 4E-H), albeit at slightly lower rates according to their broader seasonal distribution. Finally, as the density of *T. cruzi*

in their blood decreases, the hosts enter a non-infectious chronic phase. The monthly variations in the proportion of individuals in this chronic stage were even lower as they included infections of the previous years, but in the short-lived rodents where an increase of 50% of chronic cases was predicted between July and October (Fig. 4I-L).

The between-hosts differences in the incidence rate and in the seasonal variations of acute cases, which represents the main sources of infection for susceptible vectors, led us to investigate how variations in the abundance of each host type affect *T. cruzi* transmission.

Impact of the host community composition on *T. cruzi* transmission.

The analysis of the host community composition clearly shows that the abundance of the highly competent rodent hosts has a strong impact on the transmission of *T. cruzi* to the entire community (Fig. 5A). Increasing the rodent population size by 50%, made *T. cruzi* prevalence in *T. dimidiata* vector and the number of new infections in human to increase by 187%, while its incidence in rodents, dogs and cats were predicted to increase by 150%, 152% and 157%, respectively. Meanwhile, reducing the abundance of rodents present in the village by 50%, led the parasite prevalence in vectors and its incidence in all competent hosts to decreased by ~95%.

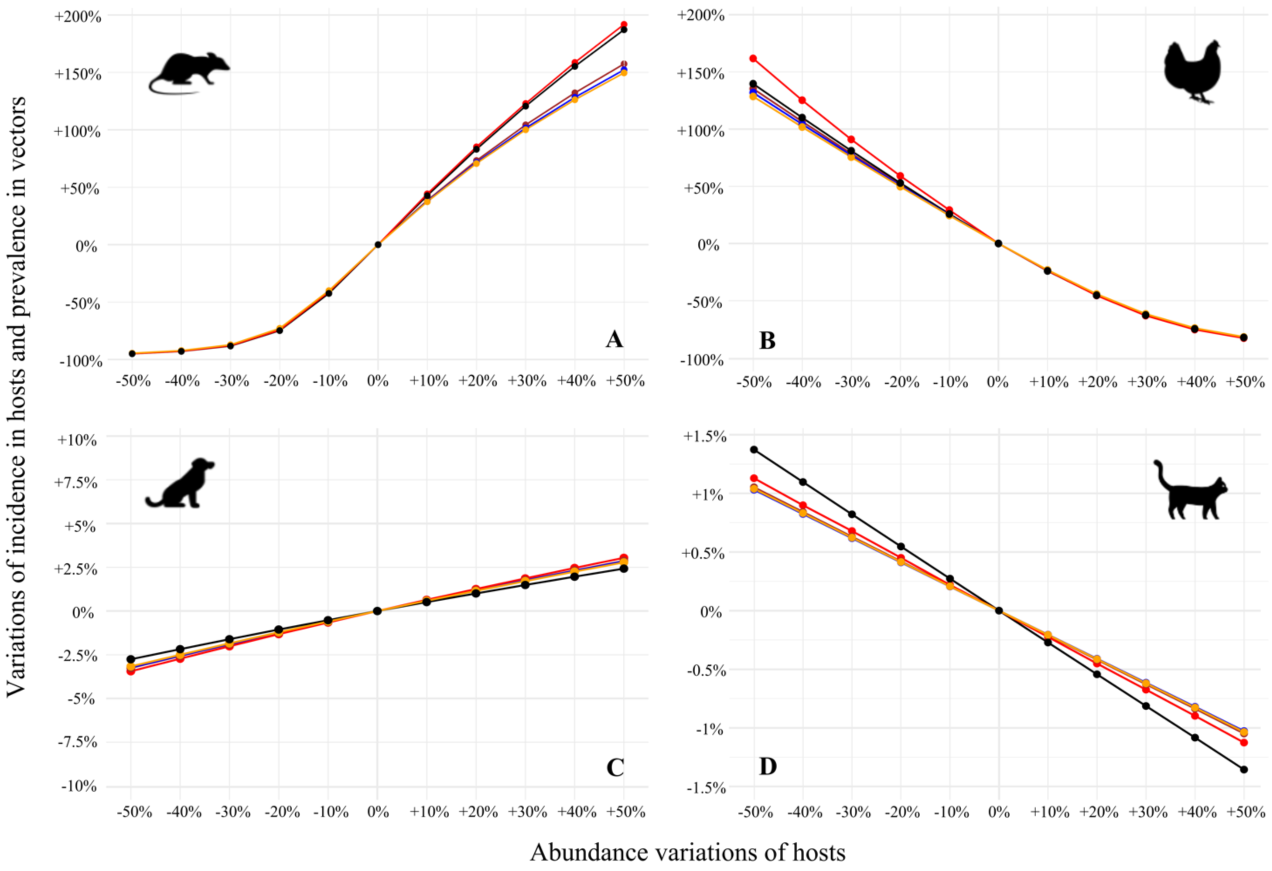

Fig 5. Predicted impact of host community composition on *T. cruzi* transmission. Variations of annual incidence in humans (red), dogs (blue), cats (brown), rodents (orange) and prevalence of infection in *T. dimidiata* (black) were predicted while varying the abundance of rodents (A), avian species (B), dogs (C) and cats (D) from -50% to +50%.

The variations in the abundance of non-competent avian hosts also had a significant, albeit opposite effect on *T. cruzi* transmission (Fig. 5B). An increase in the avian host population size of 50% induced a reduction in the *T. cruzi* prevalence in vectors of 82% and diminutions of its incidence in all hosts of ~83%. Meanwhile, a 50% reduction in the avian host population size led to a 140% larger *T. cruzi* prevalence in vectors and to increases of its incidence in humans, dogs, cats and rodents of 158%, 132%, 135% and 129%, respectively. Finally, the abundance of dogs and cats were shown to have much lower effects on *T. cruzi* transmission since varying their abundance by 50% only made its prevalence in vectors and incidence in hosts to change by ± 2.5-3% and ± 1-1.4%, respectively (Fig. 5C,D).

While the significant effects of rodent and avian populations on *T. cruzi* transmission indicate that strategies based on their control could be efficient, we aimed at testing the sensitivity of those predictions to the duration of the acute phase in hosts as variations have been observed between *T. cruzi* strains [18].

Sensitivity analysis to the duration of *T. cruzi* acute infection in hosts

The sensitivity analysis that was conducted shows that the duration of the acute infection phase of *T. cruzi* in rodent and avian hosts had little effect on their impact on the transmission of the parasite (Fig. 6A,B). The changes in the population size of rodent and avian hosts were indeed predicted to have similarly important effects on *T. cruzi* vector prevalence and annual incidence in hosts when the duration of the acute phase was progressively increased from 5 to 18 months (Fig. 6A,B).

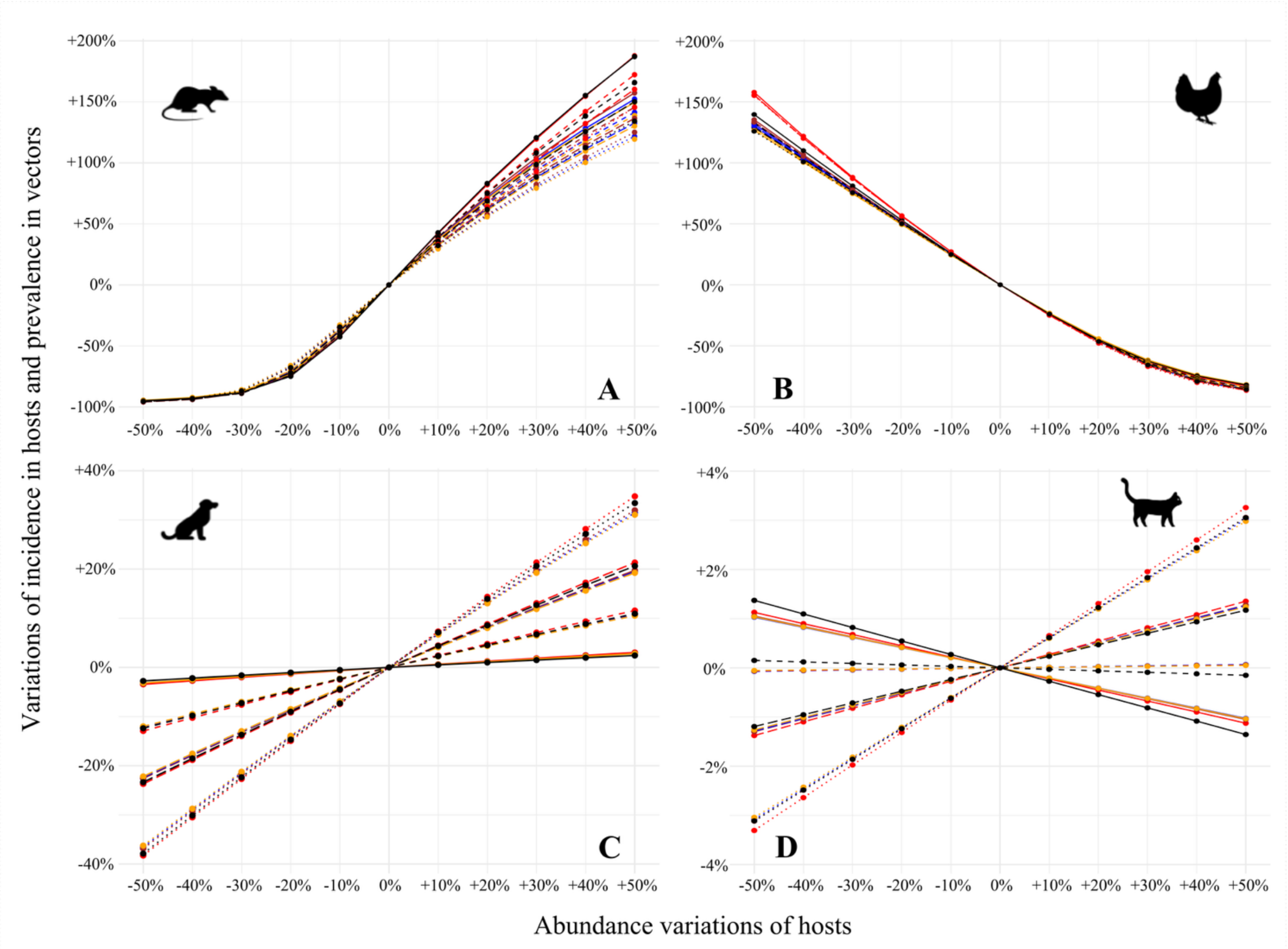

Fig 6. Predicted effect of the host community composition and the duration of *T. cruzi* acute infections on its transmission. Variations of annual incidence in humans (red), dogs (blue), cats (brown), rodents (orange) and in the prevalence of infection in *T. dimidiata* (black) were predicted by varying the abundance of rodents (A), avian species (B), dogs (C) and cats (D) from -50% to +50% and by increasing the duration of the acute phase from 5 (solid) to 8 (dashed), 12 (long-dash) and 18 (dotted) months.

Meanwhile, a longer acute and infectious phase in dogs significantly increased their impact on *T. cruzi* transmission as the effects of changing the dog population size on *T. cruzi* prevalence in vectors and incidence in hosts went up from ± 2.5-3% (for a 5-months acute phase) to variations in human

incidence that ranged from -38% to +35% (for a 18-months acute phase) (Fig. 6C). The impact of the duration of the acute phase in cats was much lower, although it had an unanticipated effect: increasing the duration of the infectious acute phase made this competent host contributing more efficiently to transmission to an extent that increasing its density went from having a diluting to an amplifying effect on *T. cruzi* transmission, which nonetheless remains lower than 3% in all hosts, even for an 18-months acute phase (Fig. 6D).

Discussion

The integration of the biological and ecological knowledge of the interactions between vectors, hosts, pathogens and their environment into eco-epidemiological models is an essential approach to assess the temporal dynamics of the risk of human infection, and anticipate the efficiency of putative control strategies [34, 53-54]. Such a challenge is especially worth tackling for zoonotic vector-borne NTDs as those complex interactions are undoubtedly deciphering their spread [6, 55-56] and our ability to reach control objectives set in the context of the Sustainable Development Goals [57-58]. In this contribution, we took advantage of the long-standing field and modelling studies of the transmission of *T. cruzi* in the Yucatan peninsula, Mexico, to build and parameterize an original SIR model of vector-borne transmission in a multi-host community that account for the within-year variations in *T. dimidiata* stage-structured demography and feeding behavior. This integrative eco-epidemiological modeling provides new quantitative insights into i) the seasonal dynamics of *T. dimidiata* abundance and blood-feeding frequency, ii) the seasonal pattern of *T. cruzi* incidence in humans and in its domesticated and synanthropic hosts, and into iii) the contribution of each of those host species to *T. cruzi* transmission within the entire community, thereby improving the key understanding required for the design of efficient control strategies.

The first key outcome of our study is that by accounting for the *T. dimidiata* stage-specific demography, feeding preferences and competitive interactions and by considering their within-year variations, our modelling successfully reproduced 86.5% of the seasonal variations in vector abundance typically observed in villages of the Yucatan peninsula. These predictions were derived under demographic and feeding conditions corresponding to our current understanding and estimates of the determinants of this non-domiciliated vector population dynamics. The model, parameterized from locally acquired demographic [28,31] and blood-feeding [37-38] data, showed that the percentage of adult insects in the village is expected to vary from 2% to 37% during the year with a peak of abundance in April, as already anticipated for the same villages of the Yucatan peninsula [32-35]. The

percentage of adult triatomines found in the village after that they migrated from the sylvatic habitat was then calculated to be on average 6.1% of insects, which is consistent with the outcomes of previous genetic and modeling studies where this proportion lied between 5% and 16% [14,59]. The model concomitantly predicted the triatomine (density-dependent) blood-feeding frequency to range from 1.58 to 2.16 meals per month during the warm and dry season, which was also found to be highly consistent with its observed value estimated to range between 1.27 and 1.67 bloodmeals from metabarcoding studies [37-38]. Overall, our stage-structured model parameterized by integrating the available data on *T. dimidiata* was therefore able to predict the local seasonal variations in vector abundance according to their (density-dependent) blood-feeding mediated development and reproduction, which allowed to look at their impact on *T. cruzi* transmission.

The second main outcome of our integrative modelling is that it allowed for the first quantitative predictions about the seasonal variations in *T. cruzi* transmission. The prevalence of *T. dimidiata* infection by *T. cruzi* was predicted to increase by 23% from March to June in the Yucatan peninsula, followed by a slow return to its initial level during the rainy and northerlies season. This predicted pattern was found to be robust to changes in both transmission and demographic parameters (Supplementary figures 2-6), and it was further confirmed by re-analyzing the field estimates of *T. dimidiata* infection prevalence that indeed show a 19% increase during the warm and dry season (March to May) [31]. Meanwhile, the prevalence of *T. dimidiata* infection by *T. cruzi* infection was predicted to vary between 12.5% and 15.4%, which belong to the 95% interval estimate derived from local field studies [31]. While these seasonal variations led to a similar pattern in rodents, with a 50% increase in their prevalence of infections by *T. cruzi*, such changes remained lower than 5% in dogs, cats, humans and rodents, which is merely explained by their longer lifetime. This, however, can be deceptive as we actually predicted a peak of new infections spread across a three-months period, from March to May, when *T. cruzi* incidence increased by 82% and 99% in dogs and cats, and by 119% in humans and rodents. This first quantification of a period of higher risk of *T. cruzi* transmission in the Yucatan peninsula, which led to 39% of all the annual new infections by *T. cruzi*, closely matched the warm and dry season, as already observed elsewhere from human's incidence epidemiological data [60-61]. Interestingly, this 3-months increase in the incidence of new cases turned into a 7-months period, from March to September, characterized by a significant increase in the proportion of hosts in the 5-months long acute phase of the disease. Overall, the number of new infections predicted for our typical village varied significantly between hosts with 0.68, 24, 13 and 1042 annual new cases among ~2000 humans, ~730 dogs, ~600 cats and ~3480 rodents, respectively, with the predicted incidence in humans being relatively closed to the epidemiological records of 0.7 new infection per year in humans

([49], Cruz-Chan, personal communication). Interestingly, while the model predicted significant seasonal variations in the incidence of *T. cruzi* and in the number of acute cases in every host species, the number of chronic cases remained roughly constant, except in short-lived rodents. This lack of temporal changes then does not allow the current epidemiological monitoring, classically implemented through the detection of antibodies, to detect the predicted seasonal pattern of infection in humans, domestic and synanthropic hosts. These new results clearly states the interest of focusing the prevention and detection of acute cases on risky warm and dry season, which could help both significantly reducing *T. cruzi* transmission and providing a better patient care through adapted treatment that are thought to considerably reduce the subsequent development of chronic disease [62].

The third key outcome of our SIR modeling is to provide further evidence that avian, rodents and dogs all play a significant role in *T. cruzi* transmission through the host community typically encountered in villages of the Yucatan peninsula, so that their management could be part of zooprophylactic control strategies. An increase in avian species abundance indeed led to a nearly linear reduction of the infection risk in the village, with an about ~80% reduction of *T. cruzi* prevalence in vectors and incidence in all competent host species when the abundance of avians was increased by up to 50%. Such predictions confirm that, while avian hosts are important blood source for triatomines, which led to their identification as a key risk factor of house infestation by the studied *T. dimidiata* populations [28], they actually are refractory hosts that overall contribute to dilute the transmission of *T. cruzi* in the Yucatan peninsula [14]. Although a similar dilution effect was also observed for *T. infestans* as avian hosts were shown to lower *T. cruzi* prevalence in humans [63] while increasing household's infestation [64-65], the management of their (peri-)domestic abundance to design cost-effective vector management remains to be tested in the field. According to our predictions, any 1% increase in the number of avian hosts could induce a ~1.6% reduction in incidence which is slightly higher, but remains consistent with the 0.45% reduction in prevalence predicted in our previous modelling [14]. Meanwhile, rodent species were also predicted to play a significant role in the local transmission of *T. cruzi*, with a decrease of 50% of their abundance in the village reducing the parasite prevalence in vectors and its incidence in competent hosts by ~95%. While such effects are consistent with empirical studies that have provided evidence of a correlation between the presence of rodents and the transmission of the parasite [12, 66-67], they turned out to be higher than previously anticipated [14]. Such an increase in the predicted effect of rodents is readily linked to the outcomes of recent metabarcoding analyses that provided a better knowledge about the host species used by *T. dimidiata* as bloodmeal sources in the studied villages [38]. The latter studies ran on over 243 vector individuals indeed showed that, among bloodmeals made on rodents, most were taken on *Mus musculus*, that

represented 88% of adult and 80% of nymph bloodmeals, and on *Rattus rattus*, that were found to account for 20% of the nymph bloodmeals. The tailoring of our model to these two important rodent species, led to consider a shorter rodent life-expectancy and a higher feeding preference by *T. dimidiata* (4 .vs. 24 months, 0.21 .vs. 0.07 in [14,17]). While the former reduced the prevalence of *T. cruzi* in those hosts and their role in transmission, the latter had an opposite and prominent effect (See Supplementary figures 2,3), which led to the overall increase in the predicted role of rodents. The prevalence of infection in rodents was indeed estimated to be ~9.9% [52] and the effects of those hosts were shown to increase as compared to previous local modelling, which actually made them more consistent with alternative assessment led on places where *T. dimidiata* is the main vector [12,66] or not [67-68]. Interestingly, in such a context, a community-based intervention conducted in Guatemala showed that a 20% reduction in rodent abundance could be achieved at a village scale and provided a 78% reduction in vector infection prevalence [12], which closely matches the 75% reduction that our model predicted when a similar control of the host community was intended. Lastly, the role of dog abundance was found to be strongly dependent upon the duration of the acute and infectious stage of the disease. Remarkably, the effect of dogs on *T. cruzi* prevalence in vectors and incidence in other hosts went from ± 2.5-3% (for a 5-months acute phase) to ± 35-38% (for a 18-months acute phase) when their abundance was increased by 50%. Such outcomes are both different and consistent with models that have been previously designed to investigate the role of those hosts in *T. cruzi* transmission and that usually rely on a simpler partition of their population into two categories; susceptible and infectious individuals. Such SI models typically predict that changing the abundance of dogs is likely to have a significant impact on *T. cruzi* prevalence in vectors and hosts [14,69]. By modelling both the acute and the chronic stage of the infection, we showed that the corresponding reduction of the duration of the infectious period can induce a significant reduction in the transmission dynamics of *T. cruzi* and the role of dogs. Meanwhile, the sensitivity analysis that we conducted provided evidence that increasing the duration of the infectious period, effectively making our SIR model converges back to an SI model, allowed to reproduce the important role of dogs identified in our previous modeling [14] while it had not impact on the predictions of the significant contributions of avian and rodent hosts to the overall transmission dynamics of *T. cruzi* in the studied villages. The latter outcome clearly unravels that by artificially expanding the duration of the infectious stage to the host lifetime, SI models are likely to have over-estimated the contributions of hosts whose life expectancy significantly exceed the duration of the acute stage, typically dogs, while we expect less (or no) effects for short-lived hosts, such as rodents. Since it has been shown that various *T. cruzi* strains can exhibit different durations of the acute infectious phase in hosts [18,70], our results suggest that predicting the role of long-lived host species, and the outcome of managing their abundance through zooprophylactic

strategies, could be sensitive to the circulating strains of parasites. Overall, the outcomes of this integrative study show that the size of avian, rodent and dog populations in villages can all have significant impact on *T. cruzi* transmission, thereby confirming that the management of the host community provides key opportunities to control Chagas disease in the Yucatan peninsula, Mexico. This strongly supports the implementation of educational and empowerment strategies embedded in One Health approach to sustainably interrupt the ongoing intra-domiciliary vector transmission of *T. cruzi* by *T. dimidiata*.

References

1. Wilson, A. L., Courtenay, O., Kelly-Hope, L. A., Scott, T. W., Takken, W., Torr, S. J., & Lindsay, S. W. (2020). The importance of vector control for the control and elimination of vector-borne diseases. *PLoS neglected tropical diseases*, *14*(1), e0007831.

2. WHO. Vector-borne diseases 2024. https://www.who.int/news-room/fact-sheets/detail/vector-borne-diseases (accessed Jan 18, 2025).

3. WHO. Neglected tropical diseases 2025. https://www.who.int/news-room/questions-and-answers/item/neglected-tropical-diseases (accessed Jan 18, 2025).

4. Lin, Y., Fang, K., Zheng, Y., Wang, H. L., & Wu, J. (2022). Global burden and trends of neglected tropical diseases from 1990 to 2019. *Journal of Travel Medicine*, *29*(3), taac031.

5. WHO. Ending NTDs: Together towards 2030. New road map for neglected tropical diseases 2021-2030. 2025. https://www.who.int/teams/control-of-neglected-tropical-diseases/ending-ntds-together-towards-2030. (accessed Jan 18, 2025).

6. Chala, B., & Hamde, F. (2021). Emerging and re-emerging vector-borne infectious diseases and the challenges for control: a review. *Frontiers in public health*, *9*, 715759.

7. Rassi, A., & Marin-Neto, J. A. (2010). Chagas disease. *The Lancet*, *375*(9723), 1388-1402.

8. Coura, J. R. (2014). The main sceneries of Chagas disease transmission. The vectors, blood and oral transmissions-A comprehensive review. *Memórias do Instituto Oswaldo Cruz*, *110*(3), 277-282.

9. Prata, A. (2001). Clinical and epidemiological aspects of Chagas disease. *The Lancet infectious diseases*, *1*(2), 92-100.

10. Reithinger, R., Ceballos, L., Stariolo, R., Davies, C. R., & Gürtler, R. E. (2005). Chagas disease control: deltamethrin-treated collars reduce Triatoma infestans feeding success on dogs. *Transactions of the Royal Society of Tropical Medicine and Hygiene*, *99*(7), 502-508.

11. Arce-Fonseca, M., Carbajal-Hernández, A. C., Lozano-Camacho, M., Carrillo-Sánchez, S. D. C., Roldán, F. J., Aranda-Fraustro, A., ... & Rodríguez-Morales, O. (2020). DNA vaccine treatment in dogs experimentally infected with Trypanosoma cruzi. *Journal of Immunology Research*, *2020*(1), 9794575.

12. De Urioste-Stone, S. M., Pennington, P. M., Pellecer, E., Aguilar, T. M., Samayoa, G., Perdomo, H. D., ... & Juárez, J. G. (2015). Development of a community-based intervention for the control of Chagas disease based on peridomestic animal management: an eco-bio-social perspective. *Transactions of the Royal Society of Tropical Medicine and Hygiene*, *109*(2), 159-167.

13. Minter-Goedbloed, E., & Croon, J. J. A. B. (1981). The insusceptibility of chickens to Trypanosoma (Schizotrypanum) cruzi. *Transactions of the Royal Society of Tropical Medicine and Hygiene*, *75*(3), 350-353.

14. Flores-Ferrer, A., Waleckx, E., Rascalou, G., Dumonteil, E., & Gourbière, S. (2019). Trypanosoma cruzi transmission dynamics in a synanthropic and domesticated host community. *PLoS neglected tropical diseases*, *13*(12), e0007902.

15. Saavedra, M., Zulantay, I., Apt, W., Castillo, J., Araya, E., Martínez, G., Rodríguez, J., 2016. Quantification by real-time PCR of Trypanosoma cruzi DNA in samples of Triatoma infestans used in xenodiagnosis of chronic Chagas disease patients. Parasit. Vectors 9, 382. doi:10.1186/s13071-016-1664-5

16. Ramírez, J. C., Cura, C. I., da Cruz Moreira, O., Lages-Silva, E., Juiz, N., Velázquez, E., ... & Schijman, A. G. (2015). Analytical validation of quantitative real-time PCR methods for quantification of Trypanosoma cruzi DNA in blood samples from Chagas disease patients. *The Journal of Molecular Diagnostics*, *17*(5), 605-615.

17. Rascalou, G., Pontier, D., Menu, F., & Gourbière, S. (2012). Emergence and prevalence of human vector-borne diseases in sink vector populations. *PloS one*, *7*(5), e36858.

18. Machado, E. M., Fernandes, A. J., Murta, S. M., Vitor, R. W., Camilo, D. J., Pinheiro, S. W., ... & Dias, J. P. (2001). A study of experimental reinfection by Trypanosoma cruzi in dogs. *The American journal of tropical medicine and hygiene*, *65*(6), 958-965.

19. Dumonteil, E., Gourbière, S., Barrera-Pérez, M., Rodriguez-Félix, E., Ruiz-Pina, H., Banos-Lopez, O., ... & Rabinovich, J. E. (2002). Geographic distribution of Triatoma dimidiata and transmission dynamics of Trypanosoma cruzi in the Yucatan peninsula of Mexico. *The American journal of tropical medicine and hygiene*, *67*(2), 176-183.

20. Péneau, J., Nguyen, A., Flores-Ferrer, A., Blanchet, D., & Gourbière, S. (2016). Amazonian Triatomine biodiversity and the transmission of Chagas disease in French Guiana: in medio stat sanitas. *PLOS Neglected Tropical Diseases*, *10*(2), e0004427.

21. Schofield, C. J., Williams, N. G., & Marshall, T. D. C. (1986). Density-dependent perception of triatomine bug bites. *Annals of Tropical Medicine & Parasitology*, *80*(3), 351-358.

22. Oscherov, E. B., Damborsky, M. P., Bar, M. E., & Gorla, D. E. (2004). Competition between vectors of Chagas disease, Triatoma infestans and T. sordida: effects on fecundity and

mortality. *Medical and Veterinary Entomology*, *18*(4), 323-328.

23. Pelosse, P., Kribs-Zaleta, C. M., Ginoux, M., Rabinovich, J. E., Gourbiere, S., & Menu, F. (2013). Influence of vectors' risk-spreading strategies and environmental stochasticity on the epidemiology and evolution of vector-borne diseases: the example of Chagas' disease. *PloS one*, *8*(8), e70830.

24. Reyes-Novelo, E., Ruíz-Piña, H., Escobedo-Ortegón, J., Rodríguez-Vivas, I., Bolio-González, M., Polanco-Rodríguez, Á., & Manrique-Saide, P. (2011). Situación actual y perspectivas para el estudio de las enfermedades zoonóticas emergentes, reemergentes y olvidadas en la Península de Yucatán, México. *Tropical and Subtropical Agroecosystems*, *14*(1), 35-54.

25. Ellis, E. A., Gomez, U. H., & Romero-Montero, J. A. (2017). Los procesos y causas del cambio en la cobertura forestal de la Península Yucatán, México. *Ecosistemas*, *26*(1), 101-111.

26. Rosecrans, K., Cruz-Martin, G., King, A., & Dumonteil, E. (2014). Opportunities for improved Chagas disease vector control based on knowledge, attitudes and practices of communities in the Yucatan Peninsula, Mexico. *PLoS neglected tropical diseases*, *8*(3), e2763.

27. Sánchez-Soto, Ma Fernanda, et al. (2024). Prevalence and transmission of the most relevant zoonotic and vector-borne pathogens in the Yucatan peninsula: A review. *PLOS Neglected Tropical Diseases* 18(7), e0012286.

28. Dumonteil, E., Nouvellet, P., Rosecrans, K., Ramirez-Sierra, M. J., Gamboa-Leon, R., Cruz-Chan, V., ... & Gourbière, S. (2013). Eco-bio-social determinants for house infestation by non-domiciliated Triatoma dimidiata in the Yucatan Peninsula, Mexico. *PLoS neglected tropical diseases*, *7*(9), e2466.

29. Payet, V., Ramirez-Sierra, M. J., Rabinovich, J., Menu, F., & Dumonteil, E. (2009). Variations in sex ratio, feeding, and fecundity of Triatoma dimidiata (Hemiptera: Reduviidae) among habitats in the Yucatan Peninsula, Mexico. *Vector-Borne and Zoonotic Diseases*, *9*(3), 243-251.

30. Reyes-Novelo, E., Ruiz-Pina, H., Escobedo-Ortegon, J., Barrera-Perez, M., Manrique-Saide, P., & Rodríguez-Vivas, R. I. (2013). Triatoma dimidiata (Latreille) abundance and infection with Trypanosoma cruzi in a rural community of Yucatan, Mexico. *Neotropical entomology*, *42*, 317-324.

31. Moo-Millan, J. I., Hernández-Andrade, A., May-Concha, I. J., de Jesús Montalvo-Balam, T., Arnal, A., Talavera-Escalante, M. J., ... & Waleckx, E. (2023). Temporal variation of Triatoma dimidiata abundance and infection with Trypanosoma cruzi in domestic and sylvatic habitats of rural Yucatan, Mexico. *Acta Tropica*, *248*, 107038.

32. Gourbière, S., Dumonteil, E., Rabinovich, J. E., Minkoue, R., & Menu, F. (2008). Demographic and dispersal constraints for domestic infestation by non-domicilated Chagas disease vectors in the Yucatan Peninsula, Mexico. *The American journal of tropical medicine and hygiene*, *78*(1), 133-139.

33. Barbu, C., Dumonteil, E., & Gourbiere, S. (2010). Characterization of the dispersal of non-domiciliated Triatoma dimidiata through the selection of spatially explicit models. *PLoS*

neglected tropical diseases, *4*(8), e777.

34. Barbu, C., Dumonteil, E., & Gourbière, S. (2009). Optimization of control strategies for non-domiciliated Triatoma dimidiata, Chagas disease vector in the Yucatán peninsula, Mexico. *PLoS neglected tropical diseases*, *3*(4), e416.

35. Barbu, C., Dumonteil, E., & Gourbière, S. (2011). Evaluation of spatially targeted strategies to control non-domiciliated Triatoma dimidiata vector of Chagas disease. *PLoS neglected tropical diseases*, *5*(5), e1045.

36. Castillo-Neyra, R., Mayorí, K. B., Sánchez, R. S., Suarez, J. A., Xie, S., Velarde, C. N., & Levy, M. Z. (2016). Heterogeneous infectiousness in guinea pigs experimentally infected with Trypanosoma cruzi. *Parasitology International*, *65*(1), 50-54.

37. Hernandez-Andrade, A. (2020). Master's thesis : Descripción de los ciclos de transmisión de *Trypanosoma cruzi* mediante la identificación de las interacciones hospedero/vector/parásito/ecotopos en localidades rurales de Yucatán, Universidad Autonoma de Yucatán.

38. Moo-Millan, J. (2024). PhD thesis : Estudio longitudinal de las interacciones hospederos/ vector/parásito/microbioma/habitat que definen los ciclos de transmision de Trypanosoma cruzi en Yucatán, Universidad Autonoma de Yucatán.

39. Instituto Nacional de Estadıstica y Geografıa. (2015). Encuesta Intercensal 2015. Retrieved 8 January 2025, from Mexico en Cifras website: https://www.inegi.org.mx

40. Dumonteil, E., Nouvellet, P., Rosecrans, K., Ramirez-Sierra, M. J., Gamboa-Leon, R., Cruz-Chan, V., ... & Gourbière, S. (2013). Eco-bio-social determinants for house infestation by non-domiciliated Triatoma dimidiata in the Yucatan Peninsula, Mexico. *PLoS neglected tropical diseases*, *7*(9), e2466.

41. Panti-May, J. A., Hernández-Betancourt, S., Ruíz-Piña, H., & Medina-Peralta, S. (2012). Abundance and population parameters of commensal rodents present in rural households in Yucatan, Mexico. *International Biodeterioration & Biodegradation*, *66*(1), 77-81.

42. Dumonteil, E., Ruiz-Piña, H., Rodriguez-Félix, E., Barrera-Pérez, M., Ramirez-Sierra, M. J., Rabinovich, J. E., & Menu, F. (2004). Re-infestation of houses by Triatoma dimidiata after intra-domicile insecticide application in the Yucatan peninsula, Mexico. *Memórias do Instituto Oswaldo Cruz*, *99*, 253-256.

43. Bosseno, M. F., García, L. S., Baunaure, F., Gastelúm, E. M., Gutierrez, M. S., Kasten, F. L., ... & Brenière, S. F. (2006). Identification in triatomine vectors of feeding sources and Trypanosoma cruzi variants by heteroduplex assay and a multiplex miniexon polymerase chain reaction. *The American journal of tropical medicine and hygiene*, *74*(2), 303-305.

44. Catalá, S. I. L. V. I. A. (1991). The biting rate of Triatoma infestans in Argentina. *Medical and Veterinary Entomology*, *5*(3), 325-333.

45. Zeledón, R., Guardia, V. M., Zúñiga, A., & Swartzwelder, J. C. (1970). Biology and ethology of Triatoma dimidiata (Latreille, 1811): I. Life cycle, amount of blood ingested, resistance to

starvation, and size of adults. *Journal of medical entomology*, *7*(3), 313-319.

46. Zeledon R. (1981). *El Triatoma Dimidiata (Latreille, 1811) y su Relacion con la Enfermedad de Chagas*. San Jose,Costa Rica: UNED.

47. Nogueda-Torres, B., Montañez-Valdez, O. D., Michel-Parra, J. G., Martínez-Grant, D. M., & Martínez-Ibarra, J. A. (2021). Biological parameters of three populations of Triatoma dimidiata ss (Hemiptera: Reduviidae) from Western Mexico. *Journal of Medical Entomology*, *58*(6), 2114-2123.

48. Marín-Ortiz, J. C., Parra-Henao, G., Altamiranda-Saavedra, M., & Jaramillo-O, N. (2022). Characterization of Feeding Behavior and its Relationship With the Longevity of Wild and Peridomestic Triatoma dimidiata, Latreille 1811 (Hemiptera, Reduviidae) Under Laboratory Conditions. *Journal of Medical Entomology*, *59*(6), 1911-1920.

49. Gamboa-León, R., Ramirez-Gonzalez, C., Pacheco-Tucuch, F. S., O'Shea, M., Rosecrans, K., Pippitt, J., ... & Buekens, P. (2014). Seroprevalence of Trypanosoma cruzi among mothers and children in rural Mayan communities and associated reproductive outcomes. *The American Journal of Tropical Medicine and Hygiene*, *91*(2), 348.

50. Jimenez-Coello, M., Poot-Cob, M., Ortega-Pacheco, A., Guzman-Marin, E., Ramos-Ligonio, A., Sauri-Arceo, C. H., & Acosta-Viana, K. Y. (2008). American trypanosomiasis in dogs from an urban and rural area of Yucatan, Mexico. *Vector-Borne and Zoonotic Diseases*, *8*(6), 755-762.

51. Jiménez-Coello, M., Acosta-Viana, K. Y., Guzman-Marin, E., Gomez-Rios, A., & Ortega-Pacheco, A. (2012). Epidemiological survey of Trypanosoma cruzi infection in domestic owned cats from the tropical southeast of Mexico. *Zoonoses and public health*, *59*, 102-109.

52. Izeta-Alberdi, A., Pech-May, A., Tun-Ku, E., Mazariegos-Hidalgo, C. J., López-Cancino, S. A., Gutiérrez, S., ... & Ramsey, J. (2023). Trypanosoma cruzi in Mexican Neotropical vectors and mammals: wildlife, livestock, pets, and human population. *salud pública de méxico*, *65*(2 mar-abr), 114-126.

53. Nouvellet, P., Dumonteil, E., & Gourbière, S. (2013). The improbable transmission of Trypanosoma cruzi to human: the missing link in the dynamics and control of Chagas disease. *PLoS neglected tropical diseases*, *7*(11), e2505.

54. Nouvellet, P., Cucunubá, Z. M., & Gourbiere, S. (2015). Ecology, evolution and control of Chagas disease: a century of neglected modelling and a promising future. *Advances in parasitology*, *87*, 135-191.

55. Webster, J. P., Gower, C. M., Knowles, S. C., Molyneux, D. H., & Fenton, A. (2016). One health–an ecological and evolutionary framework for tackling Neglected Zoonotic Diseases. *Evolutionary applications*, *9*(2), 313-333.

56. Rees, E. M., Minter, A., Edmunds, W. J., Lau, C. L., Kucharski, A. J., & Lowe, R. (2021). Transmission modelling of environmentally persistent zoonotic diseases: a systematic review. *The Lancet Planetary Health*, *5*(7), e466-e478.

57. Fitzpatrick, C., & Engels, D. (2016). Leaving no one behind: a neglected tropical disease indicator and tracers for the Sustainable Development Goals. *International health*, *8*(suppl_1), i15-i18.

58. World Health Organization. (2020). Ending the neglect to attain the Sustainable Development Goals: a road map for neglected tropical diseases 2021–2030. In *Ending the neglect to attain the sustainable development goals: a road map for neglected tropical diseases 2021–2030.*

59. Dumonteil, E., Tripet, F., Ramirez-Sierra, M. J., Payet, V., Lanzaro, G., & Menu, F. (2007). Assessment of Triatoma dimidiata dispersal in the Yucatan Peninsula of Mexico by morphometry and microsatellite markers. *The American journal of tropical medicine and hygiene*, *76*(5), 930-937.

60. Giojalas, L. C., Catalá, S. S., Asin, S. N., & Gorla, D. E. (1990). Seasonal changes in infectivity of domestic populations of Triatoma infestans. *Transactions of the Royal Society of Tropical Medicine and Hygiene*, *84*(3), 439-442.

61. Ibáñez-Cervantes, G., León-García, G., Castro-Escarpulli, G., Mancilla-Ramírez, J., Victoria-Acosta, G., Cureño-Díaz, M. A., ... & Bello-López, J. M. (2019). Evolution of incidence and geographical distribution of Chagas disease in Mexico during a decade (2007–2016). *Epidemiology & Infection*, *147*, e41.

62. Sartor, P., Colaianni, I., Cardinal, M. V., Bua, J., Freilij, H., & Gürtler, R. E. (2017). Improving access to Chagas disease diagnosis and etiologic treatment in remote rural communities of the Argentine Chaco through strengthened primary health care and broad social participation. *PLoS neglected tropical diseases*, *11*(2), e0005336.

63. Cohen, J. E., & Gurtler, R. E. (2001). Modeling household transmission of American trypanosomiasis. *Science*, *293*(5530), 694-698.

64. Gurtler, R. E., Cohen, J. E., Cecere, M. C., Lauricella, M. A., Chuit, R., & Segura, E. L. (1998). Influence of humans and domestic animals on the household prevalence of Trypanosoma cruzi in Triatoma infestans populations in northwest Argentina. *American Journal of Tropical Medicine and Hygiene*, *58*(6), 748-758.

65. Gurevitz, J. M., Ceballos, L. A., Gaspe, M. S., Alvarado-Otegui, J. A., Enríquez, G. F., Kitron, U., & Gürtler, R. E. (2011). Factors affecting infestation by Triatoma infestans in a rural area of the humid Chaco in Argentina: a multi-model inference approach. *PLoS neglected tropical diseases*, *5*(10), e1349.

66. Bustamante, D. M., De Urioste-Stone, S. M., Juárez, J. G., & Pennington, P. M. (2014). Ecological, social and biological risk factors for continued Trypanosoma cruzi transmission by Triatoma dimidiata in Guatemala. *PLoS One*, *9*(8), e104599.

67. Rosal, G. G., Nogueda-Torres, B., Villagrán, M. E., de Diego-Cabrera, J. A., Montañez-Valdez, O. D., & Martínez-Ibarra, J. A. (2018). Chagas disease: Importance of rats as reservoir hosts of Trypanosoma cruzi (Chagas, 1909) in western Mexico. *Journal of Infection and Public Health*, *11*(2), 230-233.

68. Valença-Barbosa, C., Lima, M. M., Sarquis, O., Bezerra, C. M., & Abad-Franch, F. (2014). Modeling disease vector occurrence when detection is imperfect II: drivers of site-occupancy by synanthropic Triatoma brasiliensis in the Brazilian northeast. *PLoS neglected tropical diseases*, *8*(5), e2861.

69. Fiatsonu, E., Deka, A., & Ndeffo-Mbah, M. L. (2023). Effectiveness of Systemic Insecticide Dog Treatment for the Control of Chagas Disease in the Tropics. *Biology*, *12*(9), 1235.

70. De Oliveira, M. T., Branquinho, R. T., Alessio, G. D., Mello, C. G. C., Nogueira-de-Paiva, N. C., Carneiro, C. M., ... & de Lana, M. (2017). TcI, TcII and TcVI Trypanosoma cruzi samples from Chagas disease patients with distinct clinical forms and critical analysis of in vitro and in vivo behavior, response to treatment and infection evolution in murine model. *Acta Tropica*, *167*, 108-120.

71. Maynard Smith J., & Slatkin M. (1973). The Stability of Predator-Prey Systems. Ecology, 54(2), 384-391.

72. Caswell H, 2001. *Matrix Population Models*. Sunderland, MA: Sinauer Associates.

Click here to access/download
Supporting Information
Zitoun et al. Supplementary Materials.pdf
