# Supplementary material for: Seasonal variations of Triatoma dimidiata demography and Trypanosoma cruzi transmission within its multi-host community in the Yucatan Peninsula, Mexico: Insights from an integrative SIR eco-epidemiological modelling
Source: PLoS Negl Trop Dis. 2026 Jul 15;20(7):e0014500. doi: 10.1371/journal.pntd.0014500 (PMC13384399; doi:10.1371/journal.pntd.0014500)
Supplement: Attachment — Submitted filename: Response to reviewers.docx [file pntd.0014500.s005.docx]

**Response to reviewers**

**Reviewer #1:** The authors have expanded a previously published model of T. cruzi transmission in synanthropic and domesticated host communities (an SI model introduced in 2019 by some of the same authors) into a more detailed SIR framework. This extension allows them to incorporate seasonal variation in stage-structured vector demography, as well as to explicitly distinguish between acute and chronic phases of infection in relation to parasite transmission. The new model adds a considerable degree of complexity compared to the earlier version. Overall, I appreciate the development of such modelling approaches, as they hold significant potential for advancing our understanding of Chagas disease transmission dynamics. I am therefore inclined to recommend publication of this contribution in PNTD, provided that some important points are addressed or at least discussed:

**Authors:** We thank reviewer 1 for the in depth review of our manuscript and his/her positive comments. As mentioned by the reviewer, we do believe that integrative modelling approaches hold a significant potential for advancing our understanding of Chagas disease transmission dynamics. We did our best to address all reviewer’s comments and to clarify the modelling choices and details that were not stated in a sufficiently explicit manner. All the changes made are underlined in red in the revised version of the manuscript.

**Reviewer #1 – 1 :**  While deterministic SIR-type models can be very powerful tools to explore transmission dynamics, they inherently rely on simplifying assumptions that may overlook critical sources of heterogeneity. In particular, they do not usually account for variability in human populations, such as demographic or spatial heterogeneity, nor for differences in household structure or in the two main ecological contexts where transmission occurs (intradomicile vs. peridomicile). Field evidence from Argentina illustrates how the role of reservoirs, especially dogs, can differ substantially depending on whether they are kept indoors or outdoors, which has important consequences for transmission cycles. It is, of course, acknowledged that modeling can never fully reproduce the complexity of real-world systems, and this does not diminish the value of the present contribution. However, it is important that readers are clearly aware of the limitations of the chosen framework, and that the potential impact of these assumptions on model outputs is at least considered. I therefore suggest including a short section in the Methods explicitly listing the model assumptions, and—when feasible—exploring or discussing how relaxing these assumptions might influence the results.

**Authors:** We fully agree that no model will ever reproduce reality, which would actually make it useless to help understanding the biological system at hands as it would provide an equally complex (and this time abstract) system to analyse. We also obviously agree that deterministic SIR-type models rely on discarding various sources of heterogeneity, such as the ones evocated above; natural variability between hosts, differences in household structure or uneven hosts distribution between domicile and peridomicile areas.

Although it is practically difficult to list every potential source of (individual or sub-population, temporal and spatial) heterogeneity that has been discarded in our modelling, we have added the following sentences in the ‘Material and Methods’ to be more explicit about the most common ones - that indeed typically include those mentioned by reviewer 1 - and we refer to a published review that illustrates how such components have been added in Chagas disease epidemiological models and the main outcomes of the corresponding studies:

L222 to L233: “Overall, the above model expands the SI model of *T. cruzi* transmission into a more detailed SIR framework that still obviously relies on simplifying assumptions. Those assumptions typically allow discarding some sources of heterogeneity (within human or other host species, vectors and parasites) to focus on the description of those that are being investigated. In our village scale modelling, we aimed to look at the transmission of *T. cruzi* by stage structured vector *T. dimidata* populations to typical host species while distinguishing between susceptible individuals and those in the acute and chronic phases of infection. Meanwhile, our model does not explicitly account for differences between i) habitats and households (that could potentially lead to heterogeneous infection rates at a smaller scales), ii) vectors and human or non-human hosts behaviour (that can induce heterogeneous rates of encounters) and between iii) strains of parasites (that might exhibit various levels of infectivity), that undoubtedly provide additional source of complexity to *T. cruzi* dynamics at various spatial and temporal scales (Nouvellet et al. 2015).”

**Reviewer #1 - 2:** In this sense, a further limitation relates to the deterministic nature of the model. At the community level, the choice of a deterministic SIR framework is appropriate, as it captures average trends across the entire host–vector system. I am not familiar with the specific situation in Yucatán, but in most endemic regions real communities are highly heterogeneous: households differ in construction type, human and animal host composition, and proximity to peridomestic structures. In such contexts, stochastic variation can strongly modulate transmission dynamics at the sub-community scale. For instance, chance introduction of infected vectors into only a subset of households, or random fluctuations in reservoir presence (e.g., dogs moving indoors vs. outdoors, seasonal turnover of peridomestic hosts), may result in highly localized differences in infection risk. These micro-scale variations may not be visible in a deterministic model but can critically shape community-wide prevalence over time. While I do not consider this a flaw of the modeling approach, it would be important for the authors to clarify that their results represent average expected outcomes and may not capture the range of trajectories arising from stochastic heterogeneity within the community. A brief discussion of how local variability and chance events might alter the broader epidemiological picture would strengthen the manuscript.

**Authors:** We agree that stochasticity is likely to induce microscale variations in *T. cruzi* transmission dynamics and that those might diffuse at a larger scale to influence, e.g. village scale dynamics, although we are not aware of any field evidence of such local interactions shaping a more ‘global’ epidemiological pattern of Chagas disease transmission. We would actually be very interested to read about such studies as this phenomenon is likely to require appropriate levels of vector and/or host dispersal that are presumably hard to demonstrate in the field. Still, we agree that it is worst mentioning that such a spread could potentially happen and that, in any case, the outcomes of our deterministic model represent average dynamics that do not allow capturing the variety of trajectories generated by stochastic heterogeneities.

We have therefore added the following pair of sentences in the concluding part of the ‘Material and Methods’, right after we listed the source of heterogeneities that we did not include in the model to address the previous comment of reviewer 1:

L233 to L238: “In addition, such heterogeneities are usually associated with stochastic variations generating localized microvariations in transmission dynamics that can potentially spread and affect the broader scale epidemiological patterns. It is therefore worth mentioning that outcomes of our deterministic SIR model typically represent average dynamics that do not allow capturing the entire range of trajectories arising from such fine scale stochastic variations in *T. cruzi* transmission and their potential spread through the village transmission community”.

**Reviewer #1 – 3 :**  Another important issue concerns the estimation of parameters. The manuscript states that “parameters were estimated by fitting the model predictions to the observed prevalence of T. cruzi in the host populations,” but no further details are provided. It is not clear whether a maximum likelihood framework was used for fitting, whether different prevalences were weighted differently, or which specific optimization or statistical method was applied. This lack of methodological detail limits the reproducibility and interpretability of the results, and it prevents the reader from assessing the robustness of the parameter estimates. In addition, the model appears to rely on fixed point estimates for most parameters. This is problematic because many biological and ecological parameters are expected to vary over time and across settings, or at least fall within plausible ranges rather than being static. Even if some parameters were truly constant, their estimates may still be biased or subject to error. By considering only fixed values, the model may underestimate uncertainty and potentially overstate the precision of its predictions. A more informative approach would involve either: (i) explicitly reporting plausible parameter ranges based on field or experimental evidence, (ii) exploring parameter uncertainty through sensitivity or uncertainty analyses, or (iii) discussing the implications of fixing parameters to single values.

**Authors:** We agree that the method to estimate *T. cruzi* transmission rates deserved to be presented more explicitly. These transmission rates from infectious vectors to the various host species were estimated to match the mean prevalences observed in those species in the Yucatan peninsula. To do so, the (per infectious vector contact) transmission probabilities of *T. cruzi* to each of the 4 competent hosts were varied systematically (from 0 to 1). For each tested set of 4 probability values (p_1_, p_2_, p_3_, p_4_), the mean absolute deviation between the observed and predicted prevalences was calculated over a yearly time window after the asymptotic dynamics has been reached. The quadruplet of values minimizing the mean absolute deviation provided the estimates of the per contact probability of *T. cruzi* transmission from an infectious vector to each of the 4 competent host species.

This has now been clearly specified in the Material and Methods section as follows:

L357 to L365: “The probabilities of transmission from infectious vectors to each of the competent host species were estimated to match the mean prevalences observed in those species in the Yucatan peninsula. To do so, these per (infectious vector) contact transmission probabilities of *T. cruzi* to each of the 4 competent hosts were varied systematically (from 0 to 1). For each tested set of 4 probability values (p_1_, p_2_, p_3_, p_4_), the mean absolute deviation between the observed and predicted prevalences was calculated over a yearly time window after the asymptotic dynamics has been reached. The quadruplet of values minimizing the mean absolute deviation provided the estimates of the per contact probabilities of transmission of *T. cruzi* from an infectious vector to each of the 4 competent host species.”

We obviously agree that all biological and ecological parameters are expected to vary over space and time, and that their estimates are subject to uncertainty. However, as acknowledged in the first comment of reviewer 1; no model will ever reproduce reality, and we also firmly believe that it would make such model totally useless to help understanding the biological system at hands. Accordingly, modelling is simplifying by focusing on what is needed to address some specific questions through a theoretical perspective. Our strategy in this contribution was indeed to account for seasonal variations in model parameters by focusing on those documented in the villages of the Yucatan peninsula (i.e. the seasonal pattern in the migration of adult vectors ($M_{S}, M_{I}$), in the proportions of female among *T. dimidiata* adults ($p_{f}(t)$), in the vector feeding rates of adults ($\beta_{Amax}$) and in the adult vectors death rate ($d_{A}$)), while relying the available point estimates for other parameters where no such pattern was shown (and yet could potential exist). We would like to humbly and respectfully point out that including seasonal patterns in 4 parameters in an epidemiological model accounting for the vector stage-structured demography and 5 different host species provides quite an original contribution as compared to what the literature offers in modelling CD and other vector-borne diseases.

Meanwhile, we do agree that when considering point estimates, one can expect the precision of the model predictions to be potentially overstated. We have therefore, as suggested by reviewer 1, added the following statement in the discussion to mention the potential implications of using point estimates for some of the model parameters;

L550 to L555: “Overall, even if we relied on fixed point estimates for parameters whose seasonal variations were not documented in the field, which could potentially impact the precision of the model predictions, our vector stage-structured model was still able to predict the local seasonal variations in their abundance according to their (density-dependent) blood-feeding mediated development and reproduction, which allowed us to further look at their impact on *T. cruzi* transmission.”

**Reviewer #1 - 4:**  Another concern is about the model’s projections, i.e. suggesting that a 50% reduction in dogs or rodents, or a 50% increase in avians, could reduce T. cruzi incidence by ~45%, ~150%, and ~50%, respectively. They are thought-provoking but warrant cautious interpretation. First, the model has been calibrated on a single community and lacks external validation; its predictive capacity in other settings or even in the same community but in the next years remains untested and speculative. Second, field studies from Argentina, notably Cecere et al. (1997), reported that the domiciliary density of T. infestans (not the triatomine studied here) increased when bugs fed on chickens or when hens nested indoors, suggesting that chickens may act as zoopotentiators rather than protectors of human health (doi: [https://doi.org/10.1111/j.1365-2915.1997.tb00426.x](https://emea01.safelinks.protection.outlook.com/?url=https%3A%2F%2Fdoi.org%2F10.1111%2Fj.1365-2915.1997.tb00426.x&data=05%7C02%7C%7C12876e21f5574bbce78708dea212c9dc%7C84df9e7fe9f640afb435aaaaaaaaaaaa%7C1%7C0%7C639126398741357856%7CUnknown%7CTWFpbGZsb3d8eyJFbXB0eU1hcGkiOnRydWUsIlYiOiIwLjAuMDAwMCIsIlAiOiJXaW4zMiIsIkFOIjoiTWFpbCIsIldUIjoyfQ%3D%3D%7C0%7C%7C%7C&sdata=spTV6TbjGoR9axwXoIfKM%2F5gBHzHztPPeIU0XM34ydc%3D&reserved=0)). Further, Gürtler et al. (2014) found that although chickens and dogs reduced human-bug contact rates, the relative infectivity of dogs and seasonal dynamics may still enhance transmission—consistently pointing toward a zoopotentiation effect (doi: 10.1371/journal.pntd.0002894). Considering such previous results in a related triatomine, the authors should be more cautious when mentioning these findings in the abstract, and they should ensure that the potential implications are thoroughly and critically discussed in the main text. While recommending reduction of infectious reservoirs like dogs is reasonable, advocating increases in avian populations as a control measure should be delayed until model predictions are validated against independent data and empirically supported.

**Authors:** We absolutely agree that the model is calibrated on a single local community and lacks external validation, so that its predictive capacity remains uncertain in other settings – especially if one considers triatomine species with (much) higher levels of domiciliation than *T. dimidiata* in the Yucatan peninsula. Meanwhile, in this specific system, although it has been shown that the presence of chickens increase the risk of house infestation (Dumonteil et al 2013), our modelling study confirms the previous modelling predictions that chickens could actually decrease the incidence of *T. cruzi* infection as such avians are dead-end hosts that contribute to lower the proportion of infected vectors and dilute transmission despite their positive effect on vector abundance (Flores-ferrer et al. 2019). Regarding the impact of dogs, our results concur with those evocated above has they also point towards a zoopotentiation effect.

As suggested by the reviewer, we revised the Abstract and the Discussion section to be more cautious when mentioning the potential implications of these findings for the control of Chagas disease. Those changes are as follows :

L44 to L48: “Finally, sensitivity analyses provided evidence that reducing by 50% the current abundance of dogs, rodents, and increasing the current abundance of avians by 50% could reduce *T. cruzi* yearly incidence by up to ~38%, ~95%, and ~83%, respectively, supporting a promising potential for zooprophylactic control strategies that remain to be validated by empirical field studies.”

L594 to L609: “Under the model settings, an increase in avian species abundance indeed led to a nearly linear reduction of the infection risk in the village, with an about ~80% reduction of *T. cruzi* prevalence in vectors and incidence in all competent host species when the abundance of avians was increased by up to 50%. Furthermore, any 1% increase in the number of avian hosts could induce a ~1.6% reduction in incidence which is slightly higher but remains consistent with the 0.45% reduction in prevalence predicted in our previous modelling [14]. Such predictions support that, while avian hosts are important blood source for triatomines, and therefore constitute a key risk factor of house infestation by the studied *T. dimidiata* populations [28], they actually are refractory hosts that overall contribute to dilute the transmission of *T. cruzi* in the Yucatan peninsula [14]. Interestingly, a similar dilution effect was also observed for *T. infestans* as avian hosts were shown to lower *T. cruzi* prevalence in humans [63] while increasing household’s infestation [64-65]. Finally, it is worth pointing out that our model has been calibrated on a local community and that the generality of its outcomes obviously remains to be tested. Meanwhile, the support that it provides to a promising potential for the management of (peri-)domestic abundance of avian species as part of a cost-effective vector control in the Yucatan peninsula is an encouraging perspective that will need to be validated by empirical field studies before the implementation of such zooprophylactic strategies.”

**Reviewer #1 - 5:** The manuscript states that an extensive sensitivity analysis was performed. However, the supplementary figures (S3–S6) only explore point changes in a few parameters, such as rodent life expectancy (24 months), the proportion of *T. dimidiata* adult blood meals on rodents (7%), and the duration of the acute phase. Lower acute phase values (e.g. 1-2months) were not considered, and even other key parameters appear not to have been considered. Moreover, if I understand correctly, the approach consisted of altering parameter values while keeping all other aspects of the model fixed, without re-fitting the model under the new parameter values. This is problematic because some parameters (e.g., the probability of host infection from vectors) were estimated based on the original parameter set (e.g., a six-month lifespan for rodents). Thus, when a fixed parameter is modified to assess its effect, the derived (estimated) parameters should also be re-estimated. I think a more rigorous approach would involve conducting a global sensitivity analysis (e.g., via Latin Hypercube Sampling) over plausible parameter ranges, while re-fitting the model for each parameter set. This would allow assessing parameter importance through Pearson r between parameter value and estimated incidence rate o through a variance-based sensitivity analysis, such as the total effect index (STi), which quantifies the contribution of each parameter to the variance in incidence.

**Authors:** While we perfectly understand the interest of global sensitivity analyses, we do think that the standard local sensitivity analyses we performed (e.g. Caswell, 2001) are perfectly appropriate to reach the objective of our study. We are indeed truly interested in understanding the individual effect of variations in each of the selected (not all) parameters around a ‘standard’ set of values (some of which being estimated from the model, some being estimated elsewhere and integrated as such) as they provide a good quantitative representation of the biological system. This is a very common approach to parameterize and analyse ecological models that has been repeatedly used to assess how the system works, i.e. in our case how the transmission dynamics depends on specific parameters, or how it could work if the value of those parameters were to change. Re-adjusting the values of all other parameters would help to keep on fitting but would actually make it harder to single out the effect of the targeted parameter (as we do not fully understand the individual effect of each of the others and their potential interactions). No approach has all virtues, and our strategy is based on local sensitivity analyses that are thought to complement the similar analyses intended in Flores-Ferrer et al (2019). Accordingly, we prefer not to alter the initial and, once again, very standard approach that we originally intended here as well as in Flores-Ferrer et al (2019) and in other previous contributions addressing *T. dimidiata* population dynamics in villages of the Yucatan peninsula.

Meanwhile, it is correct that we have restricted ourselves to present the sensitivity analysis to a restricted set of parameters, mostly to avoid restating results published by Flores-Ferrer et al. (2019). We naturally choose to focus on the impact of parameters that were included in our more complex SIR modelling to address the questions raised in this contribution. Accordingly, we have removed the term ‘extensive’ all throughout the manuscript as it might have suggested a more systematic approach.

**Reviewer #1 - 6:** The model assumes an acute phase duration of five months and neglects any potential transmission during the chronic phase across all hosts. This is an oversimplification that should be declared. In humans, the acute phase typically lasts only four to eight weeks (≈1–2 months) far shorter than the model assumes. Moreover, although parasitemia is much lower during the chronic phase, shedding and low-level transmission may still occur and should not be categorically excluded. The authors justify their assumption of a five-month acute phase based on xenodiagnosis data from Machado et al. (2001; doi: 10.4269/ajtmh.2001.65.958). However, this interpretation appears misleading. In that study, the acute phase was approximately one month in duration. Although xenodiagnosis yielded positive results at five months post-infection, these cases corresponded to about 10% of tests, each involving 40 nymphs (only one positive is required), which reflects residual transmission potential during the chronic phase rather than an extended acute phase. By contrast, the present model assumes that host infectivity to vectors remains as high as 0.95 for the entire five-month period. This clearly overestimates vector infection risk, as empirical evidence indicates that infectivity declines sharply after the first weeks of infection. Maintaining near-maximal transmission probability over such an extended period exaggerates the role of acute hosts and may bias the model’s predictions. It would be more appropriate to (i) parameterize the acute phase as closer to one month, in line with Machado et al. (2001), and (ii) consider lower, declining infectivity values for the subsequent chronic phase rather than assuming no transmission at all. At minimum, the authors should explicitly discuss how their chosen assumptions depart from empirical observations and how these choices might affect their results.

**Authors:** As clearly underlined by the reviewer, we neglected any potential transmission during the chronic phase to simplify the model, which is a very standard assumption used in most others Chagas disease modelling (Nouvellet et al. 2015). In a rare previous attempt at including transmission during a chronic stage of CD, the per contact probability of transmission from hosts in chronic phase to susceptible vectors was estimated to be in the range 4.2e-3 – 6.2e-3 (Rascalou et al. 2012). This represents one successful *T. cruzi* transmission per ~200 contacts, which indeed was predicted to be one the parameter with the lowest impact on the spread of *T. cruzi* (Rascalou et al. 2012) and supported the idea that the typically very low potential of transmission during the chronic phase is negligible as compared to the near-maximal transmission probability during the acute phase.

We used the xenodiagnostic data from Machado et al. (2001) to estimate the duration of the acute phase, because we think those data are the most informative ones to do so as they reproduce the actual transmission process to the vector. Indeed, although various studies, including Machado et al. (2001), showed that the parasite is detectable with significant abundance in the host blood for a 1-2 months period (Figure 1), the xenodiagnostic experiment provided evidences that transmission is possible up to 8 months after infection (Figure 2A, Machado et al. 2001), with a 50% infection rate at 5 months. Accordingly, and to avoid increasing further the complexify of the model, we chose a 5 months duration for the infectious phase of hosts, which actually corresponds to the minimum observed among the two *T. cruzi* strains studied in Machado et al. (2001). Meanwhile, we performed and discussed the outcomes of sensitivity analysis to an increase of such an estimate as this allowed for comparisons with previous epidemiological models dedicated to Chagas disease that typically are SI models assuming that hosts always remain infectious, i.e. by far more than 5 months. A part of the discussion (lines 639-655) is therefore indeed dedicated to the importance of assumptions about the within-host dynamics in CD epidemiological models.

Interestingly, we have now performed additional sensibility analysis by, this time, lowering the duration of the infectious phase in hosts (to up to 1 or 2 months), and we showed that such a reduction does not allow to obtain a *T. cruzi* prevalence in vectors consistent with field observations. Indeed, an acute phase duration of 2 months results in a 8.1% prevalence of *T. cruzi* in vectors, while the 5 months duration of acute phase that we used in the model led to a 14.1% *T. cruzi* prevalence in vectors, in line with field observations.

Overall, we do think that our simplifying assumption about the chronic stage and our estimate of the duration of the acute stage are both appropriate and an improvement as compared to previous SI models (including Flores-Ferrer et al 2019), we do agree, as suggested by reviewer 1, that it is important to make such choice totally explicit. We have thus added the following lines to the ‘Material and Methods’ section ;

L238 to L245: “Finally, the model assumes an infectious phase (I) while it neglects any potential transmission during the chronic phase (R), which is a very standard implicit assumption of most others SI Chagas disease modelling (Nouvellet et al. 2015). In a rare attempt at including transmission during a chronic stage of CD, the per contact probability of transmission from hosts in chronic phase to susceptible vectors was estimated to be as low as 4.10^-3^ to 6.10^-3^ (Rascalou et al. 2012). This represents one successful *T. cruzi* transmission per ~200 contacts, which indeed was predicted to be one the parameter with the lowest impact on the spread of *T. cruzi* (Rascalou et al. 2012) and supported the idea that such very low potential of transmission could be neglected.”

We also agree that it is worth mentioning how a lower duration of the acute stage could affect our results. We have therefore added the following statement in the part of the discussion dedicated to the importance of assumptions about the within-host dynamics in CD epidemiological models :

L647 to L651: “By modelling both the acute and the chronic stage of the infection, we showed that the corresponding reduction of the duration of the infectious period can induce a significant reduction in the transmission dynamics of *T. cruzi*, to such an extent that when the duration of the acute stage is reduced to 1-2 months the *T. cruzi* prevalence in *T. dimidiata* is predicted to be 43% lower than observed in the Yucatan peninsula.”

**Reviewer #1 - 7:** The model assumes fixed vector feeding rates on each host species and fixed recruitment and death rates for rodents. However, in real transmission systems these rates are unlikely to remain constant over time. For example, rodent populations often fluctuate seasonally, with higher abundance during the dry season in Yucatan (<https://doi.org/10.1016/j.ibiod.2011.10.006>). My concern is if such changes are expected to alter host availability and, consequently, vector feeding patterns. Ignoring these temporal variations may oversimplify the dynamics and misrepresent the contribution of different host species to transmission across seasons. A more realistic approach would involve incorporating seasonal variation in host abundance into the feeding rate estimates, or at least discussing how these fluctuations could alter the model’s predictions.

**Authors:** We agree with the reviewer that fluctuations in host populations are expected to alter host availability and vector feeding patterns, which is precisely why we intended to account for the outcome of metabarcoding studies of local vector feeding. Interestingly, the corresponding metabarcoding data used to parameterize the model actually showed no significant differences in the composition of the 270 *T. dimidiata* blood meals analysed over the year. In the absence of empirically supported seasonal patterns to account for in our modelling, we therefore considered fixed vector feeding rates. More detailed metabarcoding data are being collected to improve our power to sort out and quantify such patterns, and we will undoubtedly address this question in a subsequent contribution if such field data provide us with quantitative evidence.

We still believe that it is a very good point that deserves to be mentioned in the manuscript and we have therefore added a paragraph discussing how potential fluctuations in vector feeding patterns, related to host availability, could alter the model’s predictions.

L631 to L639: “The metabarcoding data used to parameterize the model showed no significant within-year variation in the composition of *T. dimidiata* bloodmeals despite of rodent populations of the Yucatan peninsula being shown to fluctuate seasonally with higher abundance observed during the dry season [42]. While, accordingly, we did not account for temporal changes in the vector rate of *T. cruzi* transmission to each host species, additional assessments of the seasonality in host abundances and related vector feeding patterns would undoubtedly benefit our understanding of Chagas disease transmission risk. We cannot indeed rule out that an increase in the abundance of rodents, and therefore of their presence in *T. dimidiata* blood meals during the dry season in Yucatan, would result in a higher incidence of *T. cruzi* infection.”

**Reviewer #1 – 8:** The authors estimated the maximum per adult vector blood-feeding rate (BAmax) as five meals per month, based on the maximum number of different host DNA sequences detected in metabarcoding studies of triatomine gut contents. However, this estimate may underestimate the true feeding rate. If I understood in the right way, the metabarcoding approach provides information on the maximum number of distinct host species detected over a 30-day period, but it does not account for multiple blood meals taken from the same host species. Given that triatomines may feed repeatedly on the same host individual or species within a month, the actual feeding rate could be substantially higher than the value inferred solely from the number of host species detected. If my interpretation is correct, this effect should be explicitly considered in the model, or at least discussed as a potential source of bias. Otherwise, BAmax = 5 represents only a conservative lower bound rather than the true maximum feeding frequency. Addressing this limitation would help clarify how sensitive the model outcomes are to assumptions about vector feeding intensity.

**Authors:** This interpretation of our methodological approach is not entirely correct. The metabarcoding experiment that was conducted actually allows to discriminate all distinct genotype (from the same or from different host species) detected over a 30-day period. Thus, the maximum feeding rate could be underestimated only if triatomines feed repeatedly on the exact same host individual within a month. While this could indeed happen with highly domesticated vector species, it is much more unlikely with non-domiciliated *T. dimidata* populations that do not set-up colonies inside houses and consistently tend to disperse from one house to another (Ramirez‐Sierra, 2010). Still, it cannot be entirely discarded, and we agree that the maximum per adult vector blood-feeding rate (*βAmax*) could then be slightly underestimated. Meanwhile, our estimate is consistent with its value used in a previous study accounting for the reduction of triatomine’s feeding frequency associated with intraspecific competition (Pelosse et al. 2013) and it actually leads to estimates of the actual frequency of feeding (1.58 to 2.16 meals predicted per month during the warm and dry season) that are highly consistent with values observed in metabarcoding studies (i.e. between 1.27 and 1.67 bloodmeals per month [37-38]). Finally, even if the maximum per adult vector blood-feeding rate (*βAmax*) had been estimated to be (slightly) higher, this would not have affected the model predictions about *T. cruzi* transmission since the intensity of intraspecific competition between nymphs is concomitantly set to reach the estimate of the *T. dimidiata*  adult population size in the studied village. Furthermore, given that the transmission rates of *T. cruzi* from infected vectors to healthy hosts have been estimated to fit the prevalences observed in the hosts, a larger *βAmax* would not have affected our predictions of *T. cruzi* prevalence levels in hosts either.

We have now added the following paragraph in the ‘Material and Methods’ section to clarify the outcome of the metabarcoding methodology and the lack of impact that the estimate of *βAmax* could have on our model predictions;

L541 to L550: Although our estimate of the maximum per adult vector blood-feeding rate (*βAmax*) is consistent with previous studies (Pelosse et al. 2013), the metabarcoding assessment allows to discriminate distinct genotypes (from the same or from different host species) but not to quantify the number of blood meals taken on the same host individual over a 30-day period. Although this limitation could potentially lead to underestimate the maximal feeding frequency of highly domesticated vector species, it is much less likely to bias such an estimate for non-domiciliated *T. dimidata* populations that consistently tend to disperse from one house to another in the Yucatan peninsula (Ramirez‐Sierra, 2010). Still, a larger estimate of *βAmax* would not affect our model predictions as other parameters ($\gamma_{N},p_{h}$) were concomitantly set to allow for vector abundance and *T. cruzi* prevalence in hosts to reach their observed values.

**Reviewer #1 - 9:** In the model equations, there appears to be no competition between adult and nymph vectors for blood meals. Is that correct? What is the rationale for this assumption?

**Authors:** The competition for blood meals was indeed considered to occur separately between adult and between nymph vectors. We made such a simplifying assumption as vectors’ collection showed a strong spatial segregation between those two stages (shown in Supplementary Table 1 and described in the subsection of ‘Materials and Methods’ entitled ‘Vectors feeding preferences’) that is highly consistent with the well-established ‘non-domiciliated’ status of *T. dimidata* in the studied area. Indeed, 95% of adults were found in domiciles while 82% of collected nymphs were found in backyards. This spatial partition was further supported by the observed differences in the composition of their blood meals.

**Reviewer #1 - 10:** I could not address minor points because line numbers were not provided in the PDF. For instance, the sentence “The sensitivity analysis that was conducted shows that the duration of the acute infection phase of T. cruzi in rodent and avian hosts” should be rephrased, since in the model there is no acute phase defined for avian hosts. I can comment on further minor mistakes in another round of revision.

**Authors:**  Line numbers have been provided in the revised version of the manuscript and we apologize for inconvenience in the reading of the originally submitted version. The sentence pointed out has been rephrased as:

L491 to L493: “The sensitivity analysis on the duration of the acute *T. cruzi* infection phase in competent hosts had little effect on the contribution of rodent and avian hosts on the overall transmission of the parasite (Fig. 6A,B).”

**Reviewer #1 - 11:** In the abstract it is stated that yearly incidence could be reduced by up to 150%. I understand incidence rate as the number of new cases per year, which by definition cannot be reduced below zero. Could the authors clarify what they mean by a reduction greater than 100%? As written, this statement is confusing and may mislead readers. Perhaps the authors intended to refer to the net number of cases per year or to a relative reduction compared to baseline values, but this should be explicitly clarified.

**Authors:** We do agree and we thank reviewer 1 for highlighting this erroneous and confusing report of the modelling outcomes in the abstract. An increase of 50% of the current rodents’ abundance is actually predicted to increase *T. cruzi* yearly incidence by up to ~150%. Meanwhile, reducing the abundance of rodents present in the village by 50% led the parasite prevalence in vectors and its incidence in all competent hosts to decreased by ~95%.

This part of the abstract was then corrected as:

L44 to L48: “Finally, sensitivity analyses provided evidence that reducing by 50% the current abundance of dogs, rodents, and increasing the current abundance of avians by 50% could reduce *T. cruzi* yearly incidence by up to ~38%, ~95%, and ~83%, respectively. This supports a promising potential for zooprophylactic control strategies that remain to be validated by empirical field studies.”

**References**

Caswell, H. (2001). Matrix population models: construction, analysis, and interpretation. In *Matrix population models: construction, analysis, and interpretation* (pp. 722-722).

Nouvellet, P., Cucunubá, Z. M., & Gourbiere, S. (2015). Ecology, evolution and control of Chagas disease: a century of neglected modelling and a promising future. *Advances in parasitology*, *87*, 135-191.

Pelosse P, Kribs-Zaleta CM, Ginoux M, Rabinovich JE, et al. (2013) Influence of Vectors’ Risk-Spreading Strategies and Environmental Stochasticity on the Epidemiology and Evolution of Vector-Borne Diseases: The Example of Chagas’ Disease. PLoS ONE 8(8): e70830. doi:10.1371/journal.pone.0070830

Ramirez‐Sierra, M. J., Herrera‐Aguilar, M., Gourbière, S., & Dumonteil, E. (2010). Patterns of house infestation dynamics by non‐domiciliated Triatoma dimidiata reveal a spatial gradient of infestation in rural villages and potential insect manipulation by Trypanosoma cruzi. *Tropical Medicine & International Health*, *15*(1), 77-86.

Rascalou, G., Pontier, D., Menu, F., & Gourbière, S. (2012). Emergence and prevalence of human vector-borne diseases in sink vector populations. *PloS one*, *7*(5), e36858.

**Reviewer #2:** This manuscript presents an integrative SIR eco-epidemiological model that incorporates seasonal vector demography, host community structure, and within-host infection dynamics to better understand the transmission of Trypanosoma cruzi in the Yucatán Peninsula. By building on long-term empirical data and extending previous SI approaches, the authors provide novel quantitative insights into seasonal risk, host contributions, and potential control strategies. I have greatly enjoyed reading this paper and I believe it represents an important and timely contribution to the field of Chagas disease ecology. The study nicely illustrates how mathematical modeling can be applied to neglected tropical diseases in a biologically realistic and empirically grounded way. In particular, the integration of long-term ecological, demographic, and metabarcoding data into a unified modeling framework is a major strength of the work and highlights the value of sustained field research for advancing quantitative epidemiology. Overall, I believe that this manuscript merits publication. The study is scientifically solid, methodologically innovative, and makes a valuable contribution to our understanding of Trypanosoma cruzi transmission from an eco-epidemiological perspective. My comments are mainly minor and aimed at improving clarity, structure, and the connection between modeling results and their practical implications. These are detailed in the attached annotated PDF of the manuscript.

**Authors:** We are very grateful to the reviewer for its thorough review of our manuscript and his/her very positive comments. Indeed, we firmly believe that mathematical modelling approaches integrating long-term empirical data can contribute meaningfully to our eco-epidemiological understanding of *Trypanosoma cruzi* transmission and its control. We did our best to clarify all the points raised by the reviewer, and to specify the practical implications of our modelling results, as described below. All the changes made to address the reviewer’s comments are underlined in red in the revised version of the manuscript.

**Reviewer #2: Minor Comments**

**Reviewer #2:** L67 to L69: “**...** in the absence of vaccine, the main strategy to prevent Chagas disease remains to limit contacts with vectors through insecticide spraying, house and backyard management and/or the use of screens.**”** To prevent vectorial transmission, and in terms of measures that have daily, nationwide application in Mexico, the main strategy is the systematic screening of blood in blood banks.

**Authors:** This is correct and our writing was indeed confusing as it mentioned the ‘main strategy to prevent Chagas disease’, while our contribution is clearly focused on vectorial transmission. This sentence of the abstract has been rephrased accordingly :

L67 to L69: “.., and the main strategy to prevent Chagas disease’ vectorial transmission remains to limit contacts with vectors through insecticide spraying, house and backyard management and/or the use of screens.**”**

**Reviewer #2:** L112 to L114: “This NTD is transmitted to humans and to more than 150 species of domestic and wild mammals [7] through the contact with infected feces of hematophagous insects known as triatomines [8-9].**”** Infected feces and/or oral transmission.

**Authors:** This is a good point, and the sentence has been rephrased as:

L112 to L114: “This NTD is transmitted to humans and to more than 150 species of domestic and wild mammals [7] through the contact with infected feces of hematophagous insects known as triatomines and/or by oral transmission [8-9].”

**Reviewer #2:** L117 to L120: “Some reservoir species, most notably dogs and rodents, increase the risk of human infections to such an extent that they can become the targets of control interventions [10-12], while an increase of the abundance of refractory avian species can result in a reduction of *T. cruzi* transmission to humans [13-14].**”** This fragment refers to a single feature, but it actually mentions two: one that amplifies transmission and another that dilutes transmission. I suggest revising the text to state that vector–host interactions play a key role in regulating transmission, and that the balance between competent and incompetent hosts can result in either an increase or a decrease in transmission.

**Authors:** It is correct that ‘amplification’ and ‘dilution’ effects were initially defined and are often measured with respect to the diversity *per se* of host species, rather than the abundance of specific competent or non-competent hosts. Meanwhile, in the context of controlling transmission to human it makes more sense to identify how each species of the restricted local (domiciliated and peri-domiciliated) community contributes to amplify or dilute the risk of transmission – as exemplified in our previous contribution (Flores-Ferrer et al. 2019).

We have nonetheless modified the introduction to account for both perspectives and to clarify this point as follows:

L115 to L121: “First, while triatomines feed on a vast diversity of vertebrate species, the susceptibility of these species to *T. cruzi* infection varies greatly. Among competent hosts, some reservoir species, most notably dogs and rodents, increase the risk of human infections to such an extent that they can become the targets of control interventions [10-12]. On the other hand, avian species have been shown to be refractory to *T. cruzi* transmission [13-14]. The balance between competent and non-competent hosts can therefore play a role in regulating *T. cruzi* transmission.”

**Reviewer #2:** L121 to L126: “Second, the within-host dynamics of *T. cruzi* underlies individual heterogeneities in host infectiousness [15-16]. After a primary infection, the parasite load in host’s blood increases until the infected individual becomes infectious for susceptible vectors during bloodmeals. This acute stage is typically followed by a long-term chronic phase characterized by a low parasitemia, whereby a host becomes much less infectious [17-18].**”** To maintain consistency, this second feature implies that, even among competent hosts, competence can vary temporally as a result of infection timing. I suggest that the authors retain this wording.

**Authors:** We agree that individual infectiousness varies according to the timing of infection within each competent host *species*, which we have now stated explicitly.

L122 to L126: “Second, the within-host dynamics of *T. cruzi* underlies individual heterogeneities in host infectiousness [15-16], with temporal variations as a result of infection timing among competent hosts. After a primary infection, the parasite load in host’s blood increases until the infected individual becomes infectious for susceptible vectors during bloodmeals. This acute stage is typically followed by a long-term chronic phase characterized by a low parasitemia, whereby a host becomes much less infectious [17-18].”

However, we prefer to stick to the usual terminology ‘susceptible’, ‘infectious’ and ‘recovered’ hosts when referring to their individual status since it i) truly is the common wording that directly matches the so-called ‘SIR’ models, and it ii) allows for consistency with the previous modelling by Flores-Ferrer et al. 2019, which we elaborated from in this contribution.

**Reviewer #2:** L129 to L131: “Indeed, individual vector feeding frequency has been shown to decrease with vector density as a result of intraspecific interference competition associated with an increase in triatomine biting perception that trigger defensive behavioural responses from their hosts [14, 21-23].**”** Moreover, this can trigger oral transmission through predation, increasing host infection rates and potentially influencing the next generation or season of infection in vectors.

**Authors:** This is correct. Although we are not addressing oral transmission in our modelling, we have mentioned this possibility by modifying the quoted sentence as follows;

L128 to L 132: “Indeed, individual vector feeding frequency has been shown to decrease with vector density as a result of intraspecific interference competition associated with an increase in triatomine biting perception that trigger defensive behavioral responses from their host [14, 21-23] that can potentially lead to oral transmission through predation.”

**Reviewer #2:** L140 to L141: “The Yucatan peninsula has experienced an increasing transmission of vector-borne and zoonotic diseases since the 1960s [24]” Although a positive temporal trend in incidence is likely, the cited reference does not address this issue. I suggest finding an alternative reference to support this statement.

**Authors:** The reference used to support this statement has been changed to:

Sánchez-Soto, M. F., Gaona, O., Vigueras-Galván, A. L., Suzán, G., Falcón, L. I., & Vázquez-Domínguez, E. (2024). Prevalence and transmission of the most relevant zoonotic and vector-borne pathogens in the Yucatan peninsula: A review. *PLOS Neglected Tropical Diseases*, *18*(7), e0012286.

**Reviewer #2:** L237: “Hosts demography”. Why does the model consider an age structure only for vectors but not for hosts? It is reasonable to expect host susceptibility to be age-dependent, and there is published evidence supporting this (e.g., Maloney et al. 2010; <https://doi.org/10.1645/GE-2312.1>). Age can influence key processes such as vector access to juveniles or the dispersal capacity of adults, which in turn may affect transmission dynamics. In addition, host sex is another important factor that is not considered. There is extensive literature showing that sex is a major determinant of parasite infection rates in individuals (e.g., the sex-bias hypothesis; Zuk & McKean 1996, <https://doi.org/10.1016/S0020-7519(96)80001-4>). Incorporating host age and sex structure could therefore improve the biological realism and explanatory power of the model.

**Authors:** We do agree that host age and sex structure are factors that can affect transmission dynamics. Meanwhile, the main objective of this study was to investigate how density-dependence mechanisms and seasonality acting throughout the stage-structured vector life cycle affect the *T. cruzi* transmission and we intended to do so while accounting for all the potential infectious status of the hosts, resulting in the extension of the previously developed SI approaches into SIR models. Those key features already represent significative changes to the initial modelling framework provided in Flores-Ferrer et al 2019, so that we could not add even more complexity in a single step. This would have made the comparison of the new results with the previously published ones difficult, and it would surely have weakened our ability to identify the influence of the new determinants accounted for in this new modelling. Last but not least, although we thank the reviewer for this valuable suggestion as we genuinely agree that incorporating host age and sex structure could further improve the biological realism and explanatory power of the model, we shall recognize that we do lack data on their impacts on *T. cruzi* infection in the different hosts species to be able to properly include them into our modelling.

**Reviewer #2:** L270 to L271: “The egg production by *T. dimidiata* females was set as the product of the per adult blood-feeding rate 𝛽A(𝐴, 𝐻) and the fertility per blood meal (b_A_).” For egg production, values can be obtained from Montes de Oca-Aguilar et al. (2023; <https://doi.org/10.3389/fevo.2022.805040>). In that study, the authors quantified egg production of Triatoma dimidiata across several landscapes in Yucatán.

**Authors:** We are very grateful to the referee for pointing this work to our attention, and we have indeed added this reference as described in the following comment.

**Reviewer #2:** L284 to L287: “Considering the experimentally observed *T. dimidiata* maximum blood-feeding rate of ~5 meals per month [37-38], the fertility per blood meal can be estimated to be 1088/(5*(726/30)) = 9 eggs per meal under laboratory conditions.” This is actually roughly similar to what was reported by Montes de Oca-Aguilar et al. (2023) for T. dimidiata populations in Yucatán.

**Authors:** We added this reference by complementing the above sentence as follows :

L307 to L311: **“**Considering the experimentally observed *T. dimidiata* maximum blood-feeding rate of ~5 meals per month [37-38], the fertility per blood meal can be estimated to be 1088/(5*(726/30)) = 9 eggs per meal under laboratory conditions, in lines with estimates from Montes de Oca-Aguilar et al. [47] for various *T. dimidiata* populations in Yucatán.**”**

**Reviewer #2:** L367: “... as repeatedly observed in the Yucatan peninsula [19, 29-31].” This would be more appropriate for the Discussion section.

**Authors:** This sentence has been moved to the Discussion section as :

L530 to L531: “..with a peak of abundance in April, as already anticipated for the same villages of the Yucatan peninsula [32-35].”

**Reviewer #2:** L381 to L382: “with the latter roughly corresponding to the maximal amount of time between bloodmeals, i.e. 3 to 4 months, that is required for survival [48].” This would be more appropriate for the Discussion section.

**Authors:** This sentence has been moved to the Discussion section as :

L538 to L541: “Triatomine blood-feeding frequency is then predicted to slowly decrease until they consume only 0.25 to 0.33 bloodmeals per month during the rainy and northerlies season, which roughly corresponds to the maximal amount of time between bloodmeals, i.e. 3 to 4 months, that is required for survival [48].”

**Reviewer #2:** L381 to L382: “These temporal variations in the number of *T. dimidiata* adults and their frequency of bloodmeals are likely to affect *T. cruzi* transmission to competent hosts.” This would be more appropriate for the Discussion section.

**Authors:** We would prefer not to remove this sentence from the Results section as we think it helps smoothing the transition from the presentation of the predictions about the seasonal dynamics of *T. dimidiata* in villages to the description of the results about the seasonal dynamics of *T. cruzi* transmission.

**Reviewer #2:** L387 to L388: “... provided the first quantitative predictions of the existence of seasonal variations in the infection of vectors and competent hosts.” This would be more appropriate for the Discussion section.

**Authors:** This sentence was modified to better fit the result section as:

L414 to L416: “The SIR model, parameterized to fit the average prevalence of *T. cruzi* infection observed in the *T. dimidiata* population and the host community, then provided quantitative predictions of the existence of seasonal variations in the infection of vectors and competent hosts (Fig. 3A).”

**Reviewer #2:** L486 to L497: “The integration of the biological and ecological knowledge of the interactions between vectors, hosts, pathogens and their environment into eco-epidemiological models is an essential approach to assess the temporal dynamics of the risk of human infection, and anticipate the efficiency of putative control strategies [34, 53-54]. Such a challenge is especially worth tackling for zoonotic vector-borne NTDs as those complex interactions are undoubtedly deciphering their spread [6, 55-56] and our ability to reach control objectives set in the context of the Sustainable Development Goals [57-58]. In this contribution, we took advantage of the long-standing field and modelling studies of the transmission of *T. cruzi* in the Yucatan peninsula, Mexico, to build and parameterize an original SIR model of vector-borne transmission in a multi-host community that account for the within-year variations in *T. dimidiata* stage-structured demography and feeding behaviour. This integrative eco-epidemiological modeling provides new quantitative insights into i) the seasonal dynamics of *T. dimidiata* abundance and blood-feeding frequency, ii) the seasonal pattern of *T. cruzi* incidence in humans and in its domesticated and synanthropic hosts, and into iii) the contribution of each of those host species to *T. cruzi* transmission within the entire community, thereby improving the key understanding required for the design of efficient control strategies.” This paragraph reads more like the final paragraph of the Introduction. I suggest starting the Discussion by highlighting the most relevant contribution of the article in the context of recent literature.

**Authors:** We agree that some of the points presented in this first paragraph were already stated in the Introduction and could then be removed from the Discussion. Following reviewer’ comment, we have significantly shortened the first paragraph so that it now mostly highlights the originality of the proposed approach and results, while the key outcomes are discussed with respect to the literature in the following paragraphs, as it was already done in the first version of this manuscript. The first paragraph now reads :

L515 to L522: “In this contribution, we took advantage of the long-standing field and modelling studies of the transmission of *T. cruzi* in the Yucatan peninsula, Mexico, to build and parameterize an original SIR model of vector-borne transmission in a multi-host community that account for the within-year variations in *T. dimidiata* stage-structured demography and feeding behaviour. This integrative eco-epidemiological modelling provides the first quantitative insights into the seasonal dynamics of *T. dimidiata* abundance and blood-feeding frequency, and their impact on the seasonal pattern of *T. cruzi* incidence in humans and in its domesticated and synanthropic hosts, thereby improving the key understanding required for the design of efficient control strategies.

**Reviewer #2:** L587 to L590: “Such outcomes are both different and consistent with models that have been previously designed ...” This is confusing.

**Authors:** This statement was indeed confusing and has been changed as:

L643 to L647: Although most of our predictions are consistent with previous models that have been designed to investigate the role of these hosts in *T. cruzi* transmission, those typically rely on a simpler partition of their population into two categories; susceptible and infectious individuals. However, such SI models predict that changing the abundance of dogs is likely to have a significant impact on *T. cruzi* prevalence in vectors and hosts [14,66].
