# Supplementary material for: Seasonal variations of Triatoma dimidiata demography and Trypanosoma cruzi transmission within its multi-host community in the Yucatan Peninsula, Mexico: Insights from an integrative SIR eco-epidemiological modelling
Source: PLoS Negl Trop Dis. 2026 Jul 15;20(7):e0014500. doi: 10.1371/journal.pntd.0014500 (PMC13384399; doi:10.1371/journal.pntd.0014500)
Supplement: Peer review history

# Peer review: Decision Letter 0

**DOI:** 10.1371/journal.pntd.0014500.r001

**Authors:** Paul O. Mireji

26 Jan 2026

PNTD-D-25-01227

Seasonal variations of Triatoma dimidiata demography and Trypanosoma cruzi transmission within its multi-host community in the Yucatan peninsula, Mexico: insights from an integrative SIR eco-epidemiological modelling

PLOS Neglected Tropical Diseases

Dear Dr. Zitoun,

Thank you for submitting your manuscript to PLOS Neglected Tropical Diseases. After careful consideration, we feel that it has merit but does not fully meet PLOS Neglected Tropical Diseases's publication criteria as it currently stands. Therefore, we invite you to submit a revised version of the manuscript that addresses the points raised during the review process.

Please submit your revised manuscript within by Mar 27 2026 11:59PM. If you will need more time than this to complete your revisions, please reply to this message or contact the journal office at plosntds@plos.org. When you're ready to submit your revision, log on to https://www.editorialmanager.com/pntd/ and select the 'Submissions Needing Revision' folder to locate your manuscript file.

Please include the following items when submitting your revised manuscript:

* A letter that responds to each point raised by the editor and reviewer(s). You should upload this letter as a separate file labeled 'Response to Reviewers'. This file does not need to include responses to any formatting updates and technical items listed in the 'Journal Requirements' section below.

* A marked-up copy of your manuscript that highlights changes made to the original version. You should upload this as a separate file labeled 'Revised Manuscript with Track Changes'.

* An unmarked version of your revised paper without tracked changes. You should upload this as a separate file labeled 'Manuscript'.

If you would like to make changes to your financial disclosure, competing interests statement, or data availability statement, please make these updates within the submission form at the time of resubmission. Guidelines for resubmitting your figure files are available below the reviewer comments at the end of this letter.

We look forward to receiving your revised manuscript.

Kind regards,

Paul O. Mireji, PhD

Section Editor

PLOS Neglected Tropical Diseases

Paul Mireji

Section Editor

PLOS Neglected Tropical Diseases

Shaden Kamhawi

co-Editor-in-Chief

PLOS Neglected Tropical Diseases

orcid.org/0000-0003-4304-636XX

Paul Brindley

co-Editor-in-Chief

PLOS Neglected Tropical Diseases

orcid.org/0000-0003-1765-0002

**Journal Requirements:**

1) We ask that a manuscript source file is provided at Revision. Please upload your manuscript file as a .doc, .docx, .rtf or .tex. If you are providing a .tex file, please upload it under the item type u2018LaTeX Source Fileu2019 and leave your .pdf version as the item type u2018Manuscriptu2019.

2) Please upload all main figures as separate Figure files in .tif or .eps format. For more information about how to convert and format your figure files please see our guidelines:

https://journals.plos.org/plosntds/s/figures

3) We have noticed that you have uploaded Supporting Information files, but you have not included a list of legends. Please add a full list of legends for your Supporting Information files after the references list.

4) Some material included in your submission may be copyrighted. According to PLOSu2019s copyright policy, authors who use figures or other material (e.g., graphics, clipart, maps) from another author or copyright holder must demonstrate or obtain permission to publish this material under the Creative Commons Attribution 4.0 International (CC BY 4.0) License used by PLOS journals. Please closely review the details of PLOSu2019s copyright requirements here: PLOS Licenses and Copyright. If you need to request permissions from a copyright holder, you may use PLOS's Copyright Content Permission form.

Please respond directly to this email and provide any known details concerning your material's license terms and permissions required for reuse, even if you have not yet obtained copyright permissions or are unsure of your material's copyright compatibility. Once you have responded and addressed all other outstanding technical requirements, you may resubmit your manuscript within Editorial Manager.

Potential Copyright Issues:

- Figures 1, 4, 5, 6, S3, S4, S5, and S6. Please confirm whether you drew the images / clip-art within the figure panels by hand. If you did not draw the images, please provide (a) a link to the source of the images or icons and their license / terms of use; or (b) written permission from the copyright holder to publish the images or icons under our CC BY 4.0 license. Alternatively, you may replace the images with open source alternatives. See these open source resources you may use to replace images / clip-art:

- https://commons.wikimedia.org

- https://openclipart.org/.

**Reviewers' Comments:**

Reviewer's Responses to Questions

**Key Review Criteria Required for Acceptance?**

As you describe the new analyses required for acceptance, please consider the following:

**Methods**

-Are the objectives of the study clearly articulated with a clear testable hypothesis stated?

-Is the study design appropriate to address the stated objectives?

-Is the population clearly described and appropriate for the hypothesis being tested?

-Is the sample size sufficient to ensure adequate power to address the hypothesis being tested?

-Were correct statistical analysis used to support conclusions?

-Are there concerns about ethical or regulatory requirements being met?

Reviewer #1: (No Response)

Reviewer #2: Overall, I find that the methods are sound, well justified, and appropriate to address the objectives of the study. The authors implement an original and robust eco-epidemiological SIR framework to model Trypanosoma cruzi transmission in a multi-host community, which represents a substantial methodological advance over previous SI approaches used for this system. The objectives are clearly stated: to integrate seasonal vector demography and within-host infection dynamics into an SIR framework in order to predict seasonal variation in T. cruzi transmission and to evaluate the contribution of different host species to overall transmission. While the manuscript is more predictive and exploratory than hypothesis-driven in the strict experimental sense, the modeling framework generates explicit, testable predictions (e.g., seasonal peaks in incidence, differential roles of hosts, and effects of community composition). No major concerns about ethical issues. The study is based on previously published field data and long-term monitoring programs that were presumably conducted under appropriate ethical and regulatory approvals. There is no indication of ethical shortcomings in the modeling or in the use of empirical data.

**********

**Results**

-Does the analysis presented match the analysis plan?

-Are the results clearly and completely presented?

-Are the figures (Tables, Images) of sufficient quality for clarity?

Reviewer #1: (No Response)

Reviewer #2: The Results section is well constructed and closely follows the objectives laid out in the Methods. The analyses are presented in a logical sequence, starting with the validation of seasonal patterns in vector abundance and then moving toward predictions of transmission dynamics, host-specific contributions, and the influence of key epidemiological parameters, which makes the results easy to follow and scientifically coherent. The findings show how seasonal changes in vector populations, together with differences among host species and infection stages, shape the dynamics of Trypanosoma cruzi transmission. The inclusion of sensitivity analyses is particularly valuable, as it demonstrates that the main conclusions are robust and not overly dependent on specific parameter choices. The figures and tables are generally clear and effective in translating complex model outputs into intuitive patterns, and the supplementary material provides important additional support without overloading the main text. I have, however, included specific comments in the attached annotated PDF of the manuscript indicating that some sentences in the Results section read more like interpretation or broader contextualization and would be more appropriate in the Discussion. These are minor issues of structure rather than content, and addressing them would further strengthen the clarity and focus of the Results section. Overall, the Results are clear, complete, and well aligned with the study’s objectives, and they provide strong support for the authors’ conclusions.

**********

**Conclusions**

-Are the conclusions supported by the data presented?

-Are the limitations of analysis clearly described?

-Do the authors discuss how these data can be helpful to advance our understanding of the topic under study?

-Is public health relevance addressed?

Reviewer #1: (No Response)

Reviewer #2: Overall, the conclusions are well structured and clearly supported by the data and analyses presented in the manuscript. The main claims regarding the seasonal concentration of transmission risk, the strong influence of host community composition, and the relevance of avian, rodent, and dog populations in shaping Trypanosoma cruzi dynamics are fully consistent with the model outputs and sensitivity analyses. The conclusions accurately reflect the results and appropriately emphasize the originality and value of using an SIR framework to capture both seasonal vector demography and within-host infection dynamics.

However, while the conclusions are scientifically solid, they could be further strengthened by slightly improving the transition from modeling outcomes to practical implications. The public health relevance is clearly present, particularly in the context of One Health and zooprophylactic strategies, but it remains mostly conceptual. A more explicit discussion of how these predictions could realistically inform control actions would enhance the applied value of the study. For instance, reducing dog populations, although effective in the model, may be socially and culturally difficult in many communities where dogs play important roles. It may therefore be useful to briefly discuss alternative strategies aimed at reducing or interrupting contact between dogs and vectors (e.g., housing improvements, dog management practices, use of insecticide-treated collars, or spatial separation of resting areas). Similarly, the strong role of rodents highlighted by the model opens the opportunity to discuss experiences from other regions where rodent management or environmental sanitation has been used as part of integrated vector control strategies. Although detailed implementation guidelines are beyond the scope of the work, acknowledging these practical dimensions would increase the public health impact of the conclusions.

Finally, while limitations are partly addressed through sensitivity analyses, a more explicit mention of the main modeling assumptions and uncertainties in the Conclusions would further strengthen this section.

**********

**Editorial and Data Presentation Modifications?**

Use this section for editorial suggestions as well as relatively minor modifications of existing data that would enhance clarity. If the only modifications needed are minor and/or editorial, you may wish to recommend “Minor Revision” or “Accept”.

Reviewer #1: (No Response)

Reviewer #2: Minor Revision

**********

**Summary and General Comments**

Use this section to provide overall comments, discuss strengths/weaknesses of the study, novelty, significance, general execution and scholarship. You may also include additional comments for the author, including concerns about dual publication, research ethics, or publication ethics. If requesting major revision, please articulate the new experiments that are needed.

Reviewer #1: Review on PNTD-D-25-01227 by Zitoun et al.

The authors have expanded a previously published model of T. cruzi transmission in synanthropic and domesticated host communities (an SI model introduced in 2019 by some of the same authors) into a more detailed SIR framework. This extension allows them to incorporate seasonal variation in stage-structured vector demography, as well as to explicitly distinguish between acute and chronic phases of infection in relation to parasite transmission.

The new model adds a considerable degree of complexity compared to the earlier version. Overall, I appreciate the development of such modeling approaches, as they hold significant potential for advancing our understanding of Chagas disease transmission dynamics. I am therefore inclined to recommend publication of this contribution in PNTD, provided that some important points are addressed or at least discussed:

1.While deterministic SIR-type models can be very powerful tools to explore transmission dynamics, they inherently rely on simplifying assumptions that may overlook critical sources of heterogeneity. In particular, they do not usually account for variability in human populations, such as demographic or spatial heterogeneity, nor for differences in household structure or in the two main ecological contexts where transmission occurs (intradomicile vs. peridomicile). Field evidence from Argentina illustrates how the role of reservoirs, especially dogs, can differ substantially depending on whether they are kept indoors or outdoors, which has important consequences for transmission cycles. It is, of course, acknowledged that modeling can never fully reproduce the complexity of real-world systems, and this does not diminish the value of the present contribution. However, it is important that readers are clearly aware of the limitations of the chosen framework, and that the potential impact of these assumptions on model outputs is at least considered. I therefore suggest including a short section in the Methods explicitly listing the model assumptions, and—when feasible—exploring or discussing how relaxing these assumptions might influence the results.

2.In this sense, a further limitation relates to the deterministic nature of the model. At the community level, the choice of a deterministic SIR framework is appropriate, as it captures average trends across the entire host–vector system. I am not familiar with the specific situation in Yucatán, but in most endemic regions real communities are highly heterogeneous: households differ in construction type, human and animal host composition, and proximity to peridomestic structures. In such contexts, stochastic variation can strongly modulate transmission dynamics at the sub-community scale. For instance, chance introduction of infected vectors into only a subset of households, or random fluctuations in reservoir presence (e.g., dogs moving indoors vs. outdoors, seasonal turnover of peridomestic hosts), may result in highly localized differences in infection risk. These micro-scale variations may not be visible in a deterministic model but can critically shape community-wide prevalence over time. While I do not consider this a flaw of the modeling approach, it would be important for the authors to clarify that their results represent average expected outcomes and may not capture the range of trajectories arising from stochastic heterogeneity within the community. A brief discussion of how local variability and chance events might alter the broader epidemiological picture would strengthen the manuscript.

3.Another important issue concerns the estimation of parameters. The manuscript states that “parameters were estimated by fitting the model predictions to the observed prevalence of T. cruzi in the host populations,” but no further details are provided. It is not clear whether a maximum likelihood framework was used for fitting, whether different prevalences were weighted differently, or which specific optimization or statistical method was applied. This lack of methodological detail limits the reproducibility and interpretability of the results, and it prevents the reader from assessing the robustness of the parameter estimates. In addition, the model appears to rely on fixed point estimates for most parameters. This is problematic because many biological and ecological parameters are expected to vary over time and across settings, or at least fall within plausible ranges rather than being static. Even if some parameters were truly constant, their estimates may still be biased or subject to error. By considering only fixed values, the model may underestimate uncertainty and potentially overstate the precision of its predictions. A more informative approach would involve either: (i) explicitly reporting plausible parameter ranges based on field or experimental evidence, (ii) exploring parameter uncertainty through sensitivity or uncertainty analyses, or (iii) discussing the implications of fixing parameters to single values.

4.Another concern is about the model’s projections, i.e. suggesting that a 50% reduction in dogs or rodents, or a 50% increase in avians, could reduce T. cruzi incidence by ~45%, ~150%, and ~50%, respectively. They are thought-provoking but warrant cautious interpretation. First, the model has been calibrated on a single community and lacks external validation; its predictive capacity in other settings or even in the same community but in the next years remains untested and speculative. Second, field studies from Argentina, notably Cecere et al. (1997), reported that the domiciliary density of T. infestans (not the triatomine studied here) increased when bugs fed on chickens or when hens nested indoors, suggesting that chickens may act as zoopotentiators rather than protectors of human health (doi: https://doi.org/10.1111/j.1365-2915.1997.tb00426.x). Further, Gürtler et al. (2014) found that although chickens and dogs reduced human-bug contact rates, the relative infectivity of dogs and seasonal dynamics may still enhance transmission—consistently pointing toward a zoopotentiation effect (doi: 10.1371/journal.pntd.0002894). Considering such previous results in a related triatomine, the authors should be more cautious when mentioning these findings in the abstract, and they should ensure that the potential implications are thoroughly and critically discussed in the main text. While recommending reduction of infectious reservoirs like dogs is reasonable, advocating increases in avian populations as a control measure should be delayed until model predictions are validated against independent data and empirically supported.

5.The manuscript states that an extensive sensitivity analysis was performed. However, the supplementary figures (S3–S6) only explore point changes in a few parameters, such as rodent life expectancy (24 months), the proportion of T. dimidiata adult blood meals on rodents (7%), and the duration of the acute phase. Lower acute phase values (e.g. 1-2months) were not considered and even other key parameters appear not to have been considered. Moreover, if I understand correctly, the approach consisted of altering parameter values while keeping all other aspects of the model fixed, without re-fitting the model under the new parameter values. This is problematic because some parameters (e.g., the probability of host infection from vectors) were estimated based on the original parameter set (e.g., a six-month lifespan for rodents). Thus, when a fixed parameter is modified to assess its effect, the derived (estimated) parameters should also be re-estimated. I think a more rigorous approach would involve conducting a global sensitivity analysis (e.g., via Latin Hypercube Sampling) over plausible parameter ranges, while re-fitting the model for each parameter set. This would allow assessing parameter importance through Pearson r between parameter value and estimated incidence rate o through a variance-based sensitivity analysis, such as the total effect index (STi), which quantifies the contribution of each parameter to the variance in incidence.

6.The model assumes an acute phase duration of five months and neglects any potential transmission during the chronic phase across all hosts. This is an oversimplification that should be declared. In humans, the acute phase typically lasts only four to eight weeks (≈1–2 months) far shorter than the model assumes. Moreover, although parasitemia is much lower during the chronic phase, shedding and low-level transmission may still occur and should not be categorically excluded. The authors justify their assumption of a five-month acute phase based on xenodiagnosis data from Machado et al. (2001; doi: 10.4269/ajtmh.2001.65.958). However, this interpretation appears misleading. In that study, the acute phase was approximately one month in duration. Although xenodiagnosis yielded positive results at five months post-infection, these cases corresponded to about 10% of tests, each involving 40 nymphs (only one positive is required), which reflects residual transmission potential during the chronic phase rather than an extended acute phase. By contrast, the present model assumes that host infectivity to vectors remains as high as 0.95 for the entire five-month period. This clearly overestimates vector infection risk, as empirical evidence indicates that infectivity declines sharply after the first weeks of infection. Maintaining near-maximal transmission probability over such an extended period exaggerates the role of acute hosts and may bias the model’s predictions. It would be more appropriate to (i) parameterize the acute phase as closer to one month, in line with Machado et al. (2001), and (ii) consider lower, declining infectivity values for the subsequent chronic phase rather than assuming no transmission at all. At minimum, the authors should explicitly discuss how their chosen assumptions depart from empirical observations and how these choices might affect their results.

7.The model assumes fixed vector feeding rates on each host species and fixed recruitment and death rates for rodents. However, in real transmission systems these rates are unlikely to remain constant over time. For example, rodent populations often fluctuate seasonally, with higher abundance during the dry season in Yucatan (https://doi.org/10.1016/j.ibiod.2011.10.006). My concern is if such changes are expected to alter host availability and, consequently, vector feeding patterns. Ignoring these temporal variations may oversimplify the dynamics and misrepresent the contribution of different host species to transmission across seasons. A more realistic approach would involve incorporating seasonal variation in host abundance into the feeding rate estimates, or at least discussing how these fluctuations could alter the model’s predictions.

8.The authors estimated the maximum per adult vector blood-feeding rate (BAmax) as five meals per month, based on the maximum number of different host DNA sequences detected in metabarcoding studies of triatomine gut contents. However, this estimate may underestimate the true feeding rate. If I understood in the right way, the metabarcoding approach provides information on the maximum number of distinct host species detected over a 30-day period, but it does not account for multiple blood meals taken from the same host species. Given that triatomines may feed repeatedly on the same host individual or species within a month, the actual feeding rate could be substantially higher than the value inferred solely from the number of host species detected. If my interpretation is correct, this effect should be explicitly considered in the model, or at least discussed as a potential source of bias. Otherwise, BAmax = 5 represents only a conservative lower bound rather than the true maximum feeding frequency. Addressing this limitation would help clarify how sensitive the model outcomes are to assumptions about vector feeding intensity.

9.In the model equations, there appears to be no competition between adult and nymph vectors for blood meals. Is that correct? What is the rationale for this assumption?

10.I could not address minor points because line numbers were not provided in the PDF. For instance, the sentence “The sensitivity analysis that was conducted shows that the duration of the acute infection phase of T. cruzi in rodent and avian hosts” should be rephrased, since in the model there is no acute phase defined for avian hosts. I can comment on further minor mistakes in another round of revision.

11.In the abstract it is stated that yearly incidence could be reduced by up to 150%. I understand incidence rate as the number of new cases per year, which by definition cannot be reduced below zero. Could the authors clarify what they mean by a reduction greater than 100%? As written, this statement is confusing and may mislead readers. Perhaps the authors intended to refer to the net number of cases per year or to a relative reduction compared to baseline values, but this should be explicitly clarified.

Reviewer #2: This manuscript presents an integrative SIR eco-epidemiological model that incorporates seasonal vector demography, host community structure, and within-host infection dynamics to better understand the transmission of Trypanosoma cruzi in the Yucatán Peninsula. By building on long-term empirical data and extending previous SI approaches, the authors provide novel quantitative insights into seasonal risk, host contributions, and potential control strategies.

I have greatly enjoyed reading this paper and I believe it represents an important and timely contribution to the field of Chagas disease ecology. The study nicely illustrates how mathematical modeling can be applied to neglected tropical diseases in a biologically realistic and empirically grounded way. In particular, the integration of long-term ecological, demographic, and metabarcoding data into a unified modeling framework is a major strength of the work and highlights the value of sustained field research for advancing quantitative epidemiology.

Overall, I believe that this manuscript merits publication. The study is scientifically solid, methodologically innovative, and makes a valuable contribution to our understanding of Trypanosoma cruzi transmission from an eco-epidemiological perspective. My comments are mainly minor and aimed at improving clarity, structure, and the connection between modeling results and their practical implications. These are detailed in the attached annotated PDF of the manuscript.

**********

PLOS authors have the option to publish the peer review history of their article (what does this mean?). If published, this will include your full peer review and any attached files.

If you choose “no”, your identity will remain anonymous but your review may still be made public.

**Do you want your identity to be public for this peer review?**  For information about this choice, including consent withdrawal, please see our Privacy Policy.

Reviewer #1: No

Reviewer #2: No

[NOTE: If reviewer comments were submitted as an attachment file, they will be attached to this email and accessible via the submission site. Please log into your account, locate the manuscript record, and check for the action link "View Attachments". If this link does not appear, there are no attachment files.]

**Figure resubmission:**  While revising your submission, we strongly recommend that you use PLOS’s NAAS tool (https://ngplosjournals.pagemajik.ai/artanalysis) to test your figure files. NAAS can convert your figure files to the TIFF file type and meet basic requirements (such as print size, resolution), or provide you with a report on issues that do not meet our requirements and that NAAS cannot fix.

After uploading your figures to PLOS’s NAAS tool - https://ngplosjournals.pagemajik.ai/artanalysis, NAAS will process the files provided and display the results in the "Uploaded Files" section of the page as the processing is complete. If the uploaded figures meet our requirements (or NAAS is able to fix the files to meet our requirements), the figure will be marked as "fixed" above. If NAAS is unable to fix the files, a red "failed" label will appear above. When NAAS has confirmed that the figure files meet our requirements, please download the file via the download option, and include these NAAS processed figure files when submitting your revised manuscript.

**Reproducibility:**

To enhance the reproducibility of your results, we recommend that authors of applicable studies deposit laboratory protocols in protocols.io, where a protocol can be assigned its own identifier (DOI) such that it can be cited independently in the future. Additionally, PLOS ONE offers an option to publish peer-reviewed clinical study protocols. Read more information on sharing protocols at https://plos.org/protocols?utm_medium=editorial-email&utm_source=authorletters&utm_campaign=protocols

Attachment Submitted filename: PNTD-D-25-01227.pdf

# Author comment: Author response to Decision Letter 1

**DOI:** 10.1371/journal.pntd.0014500.r002

9 May 2026

Attachment Submitted filename: Response to reviewers.docx

# Peer review: Decision Letter 1

**DOI:** 10.1371/journal.pntd.0014500.r003

**Authors:** Paul O. Mireji

23 Jun 2026

Dear Dr. Zitoun,

We are pleased to inform you that your manuscript 'Seasonal variations of Triatoma dimidiata demography and Trypanosoma cruzi transmission within its multi-host community in the Yucatan peninsula, Mexico: insights from an integrative SIR eco-epidemiological modelling' has been provisionally accepted for publication in PLOS Neglected Tropical Diseases.

Before your manuscript can be formally accepted you will need to complete some formatting changes, which you will receive in a follow up email. A member of our team will be in touch with a set of requests.

Please note that your manuscript will not be scheduled for publication until you have made the required changes, so a swift response is appreciated.

IMPORTANT: The editorial review process is now complete. PLOS will only permit corrections to spelling, formatting or significant scientific errors from this point onwards. Requests for major changes, or any which affect the scientific understanding of your work, will cause delays to the publication date of your manuscript.

Should you, your institution's press office or the journal office choose to press release your paper, you will automatically be opted out of early publication. We ask that you notify us now if you or your institution is planning to press release the article. All press must be co-ordinated with PLOS.

Thank you again for supporting Open Access publishing; we are looking forward to publishing your work in PLOS Neglected Tropical Diseases.

Best regards,

Paul O. Mireji, PhD

Section Editor

PLOS Neglected Tropical Diseases

Paul Mireji

Section Editor

PLOS Neglected Tropical Diseases

Shaden Kamhawi

co-Editor-in-Chief

PLOS Neglected Tropical Diseases

orcid.org/0000-0003-4304-636XX

Paul Brindley

co-Editor-in-Chief

PLOS Neglected Tropical Diseases

orcid.org/0000-0003-1765-0002

***********************************************************

Reviewer's Responses to Questions

**Key Review Criteria Required for Acceptance?**

As you describe the new analyses required for acceptance, please consider the following:

**Methods**

-Are the objectives of the study clearly articulated with a clear testable hypothesis stated?

-Is the study design appropriate to address the stated objectives?

-Is the population clearly described and appropriate for the hypothesis being tested?

-Is the sample size sufficient to ensure adequate power to address the hypothesis being tested?

-Were correct statistical analysis used to support conclusions?

-Are there concerns about ethical or regulatory requirements being met?

Reviewer #1: (No Response)

Reviewer #2: Mthods are appropriate to address the stated objectives

**********

**Results**

-Does the analysis presented match the analysis plan?

-Are the results clearly and completely presented?

-Are the figures (Tables, Images) of sufficient quality for clarity?

Reviewer #1: (No Response)

Reviewer #2: Results are appropriately presented.

**********

**Conclusions**

-Are the conclusions supported by the data presented?

-Are the limitations of analysis clearly described?

-Do the authors discuss how these data can be helpful to advance our understanding of the topic under study?

-Is public health relevance addressed?

Reviewer #1: (No Response)

Reviewer #2: The conclusions are clearly supported by the data presented.

**********

**Editorial and Data Presentation Modifications?**

Use this section for editorial suggestions as well as relatively minor modifications of existing data that would enhance clarity. If the only modifications needed are minor and/or editorial, you may wish to recommend “Minor Revision” or “Accept”.

Reviewer #1: (No Response)

Reviewer #2: (No Response)

**********

**Summary and General Comments**

Use this section to provide overall comments, discuss strengths/weaknesses of the study, novelty, significance, general execution and scholarship. You may also include additional comments for the author, including concerns about dual publication, research ethics, or publication ethics. If requesting major revision, please articulate the new experiments that are needed.

Reviewer #1: The authors have addressed my previous concerns, mainly by clarifying model assumptions, parameter estimation procedures, stochastic limitations, and the cautious interpretation of zooprophylactic predictions.

Some minor English and style adjustments could further improve the manuscript, particularly if the authors aim for a more polished final version. Nevertheless, these issues are minor, the text is sufficiently clear, and I do not consider them an obstacle to publication. I would also like to congratulate the authors for this interesting and valuable work.

Reviewer #2: The authors addressed all my comments and suggestions, providing a revised manuscript that merits full consideration for publication. I believe this contribution will be a valuable reference not only for the control of Chagas disease, but also for the study of other complex infectious diseases.

**********

PLOS authors have the option to publish the peer review history of their article (what does this mean?). If published, this will include your full peer review and any attached files.

If you choose “no”, your identity will remain anonymous but your review may still be made public.

**Do you want your identity to be public for this peer review?**  For information about this choice, including consent withdrawal, please see our Privacy Policy.

Reviewer #1: No

Reviewer #2: **Yes:** Carlos N. Ibarra-Cerdeña

# Editor report: Acceptance letter

**DOI:** 10.1371/journal.pntd.0014500.r004

**Authors:** Paul O. Mireji

Dear Dr. Zitoun,

We are delighted to inform you that your manuscript, "

Seasonal variations of Triatoma dimidiata demography and Trypanosoma cruzi transmission within its multi-host community in the Yucatan peninsula, Mexico: insights from an integrative SIR eco-epidemiological modelling  ," has been formally accepted for publication in PLOS Neglected Tropical Diseases.

We have now passed your article onto the PLOS Production Department who will complete the rest of the publication process. All authors will receive a confirmation email upon publication.

The corresponding author will soon be receiving a typeset proof for review, to ensure errors have not been introduced during production. Please review the PDF proof of your manuscript carefully, as this is the last chance to correct any scientific or type-setting errors. Please note that major changes, or those which affect the scientific understanding of the work, will likely cause delays to the publication date of your manuscript. Note: Proofs for Front Matter articles (Editorial, Viewpoint, Symposium, Review, etc...) are generated on a different schedule and may not be made available as quickly.

Soon after your final files are uploaded, the early version of your manuscript will be published online unless you opted out of this process. The date of the early version will be your article's publication date. The final article will be published to the same URL, and all versions of the paper will be accessible to readers.

For Research Articles, you will receive an invoice from PLOS for your publication fee after your manuscript has reached the completed accept phase. If you receive an email requesting payment before acceptance or for any other service, this may be a phishing scheme. Learn how to identify phishing emails and protect your accounts at https://explore.plos.org/phishing.

Thank you again for supporting open-access publishing; we are looking forward to publishing your work in PLOS Neglected Tropical Diseases.

Best regards,

Shaden Kamhawi

co-Editor-in-Chief

PLOS Neglected Tropical Diseases

Paul Brindley

co-Editor-in-Chief

PLOS Neglected Tropical Diseases
